# Rational design of an artificial tethered enzyme for non-templated post-transcriptional mRNA polyadenylation by the second generation of the C3P3 system

**DOI:** 10.1038/s41598-024-55947-0

**Published:** 2024-03-02

**Authors:** Marine Le Boulch, Eric Jacquet, Naïma Nhiri, Maya Shmulevitz, Philippe H. Jaïs

**Affiliations:** 1 Eukarÿs SAS, Pépinière Genopole, 4 rue Pierre Fontaine, Genopole Entreprises Campus 3, 4 Rue Pierre Fontaine, 91000 Evry-Courcouronnes, France; 2 Institut de Chimie des Substances Naturelles, CNRS UPR2301, Université Paris-Saclay, Avenue de la Terrasse, 91198 Gif-Sur-Yvette, France; 3 Medical Microbiology and Immunology, Li Ka Shing Institute of Virology, University of Alberta, 6-142J Katz Group Centre for Pharmacy and Health Research, 114 Street NW, Edmonton, AB T6G 2E1 Canada

**Keywords:** C3P3, mRNA, Polyadenylation, Poly(A) polymerase, Artificial expression system, Expression systems, RNA, RNA modification, Transcriptional regulatory elements

## Abstract

We have previously introduced the first generation of C3P3, an artificial system that allows the autonomous in-vivo production of mRNA with ^m7^GpppN-cap. While C3P3-G1 synthesized much larger amounts of capped mRNA in human cells than conventional nuclear expression systems, it produced a proportionately much smaller amount of the corresponding proteins, indicating a clear defect of mRNA translatability. A possible mechanism for this poor translatability could be the rudimentary polyadenylation of the mRNA produced by the C3P3-G1 system. We therefore sought to develop the C3P3-G2 system using an artificial enzyme to post-transcriptionally lengthen the poly(A) tail. This system is based on the mutant mouse poly(A) polymerase alpha fused at its N terminus with an N peptide from the λ virus, which binds to BoxBr sequences placed in the 3′UTR region of the mRNA of interest. The resulting system selectively brings mPAPαm7 to the target mRNA to elongate its poly(A)-tail to a length of few hundred adenosine. Such elongation of the poly(A) tail leads to an increase in protein expression levels of about 2.5–3 times in cultured human cells compared to the C3P3-G1 system. Finally, the coding sequence of the tethered mutant poly(A) polymerase can be efficiently fused to that of the C3P3-G1 enzyme via an F2A sequence, thus constituting the single-ORF C3P3-G2 enzyme. These technical developments constitute an important milestone in improving the performance of the C3P3 system, paving the way for its applications in bioproduction and non-viral human gene therapy.

## Introduction

Many experimental and therapeutic approaches require expression of exogenous genes in eukaryotic cells. Currently, there are three common strategies to achieve exogenous gene delivery and expression. First, DNA plasmids can be introduced into cells, but the poor efficiency of nuclear localization in many cells required for exogenous gene expression limits the potency of DNA plasmid approaches. To circumvent this limitation, viruses such as adeno-associated viruses (AAV) and retroviruses can be used to deliver genomes into the nucleus of cells for expression; albeit viruses come with their own limitations in terms of production and safety^1^. Finally, lipid nanoparticles (LNP) can be used to deliver RNAs directly into the cytoplasm of cells for immediate protein production, as is used for mRNA SARS-Cov2 vaccines^2^. There are limitations to RNA-based gene expression approaches however, such as the short duration of protein expression and induction of innate signaling pathways. Given the limitations of existing strategies, we have focused on creating a DNA-based system that drives mRNA and protein expression from the cytoplasm. The rationale for this system is to harness the benefits of DNA for long term expression without requiring nuclear import.

We previously applied synthetic biology and molecular engineering to develop an artificial system called C3P3 (standing for cytoplasmic chimeric capping-prone phage polymerase), which allows the autonomous synthesis of mature target mRNAs in the cytoplasmic compartment of mammalian cells^3^. The first generation of this system (C3P3-G1) relies on single-subunit artificial chimeric enzyme created by the fusion of the DNA-dependent RNA polymerase from the K1E bacteriophage and the NP868R ^m7^GpppN-capping (cap-0) enzyme from the African Swine fever virus, separated by a flexible (G_4_S)_2_ linker. Due to their physical association, mRNA transcribed by RNA polymerase moiety is nearly fully capped by the capping enzymatic moiety, in contrast to what is observed in the absence of their physical association^3^. Such difference is explained by the restricted diffusion of large macromolecules such as mRNA and DNA caused by the gel-like viscosity of the cytoplasm or nucleoplasm^4^. Although the C3P3 enzyme functions in both of these cellular compartments, we chose to express it in the cytoplasm of the host cell. This cellular localization in fact has the advantage of avoiding the translocation of the transfected exogenous DNA from the cytoplasm towards the nucleus, which is one of the most important barriers for the delivery of exogenous DNA to the cells^5^.

The C3P3 system was designed to transcribe double-stranded DNA templates containing the target gene with C3P3 promoter, and then add a ^m7^GpppN cap-0 at the ends 5′ of the target transcripts. The proximity of these two enzymatic functions makes it possible to synthesize a target mRNA that is predominantly capped at its 5′ end^3^. Although the C3P3-G1 system is highly processive and produces large amounts of target mRNA in cultured mammalian cells, proportionally lesser amounts of protein are produced, suggesting a further bottleneck to maximum protein expression also exists during mRNA translation. For example, the C3P3-G1 expression system produced 6.7-fold more luciferase mRNA, but 2.2-fold less luciferase luminescence signal at peak relative to the standard CMV-driven Firefly luciferase expression plasmid^3^. These findings suggest that other modifications, in addition to 5′-end ^m7^GpppN capping, are required for full translatability of target transcripts.

**Figure 1:**
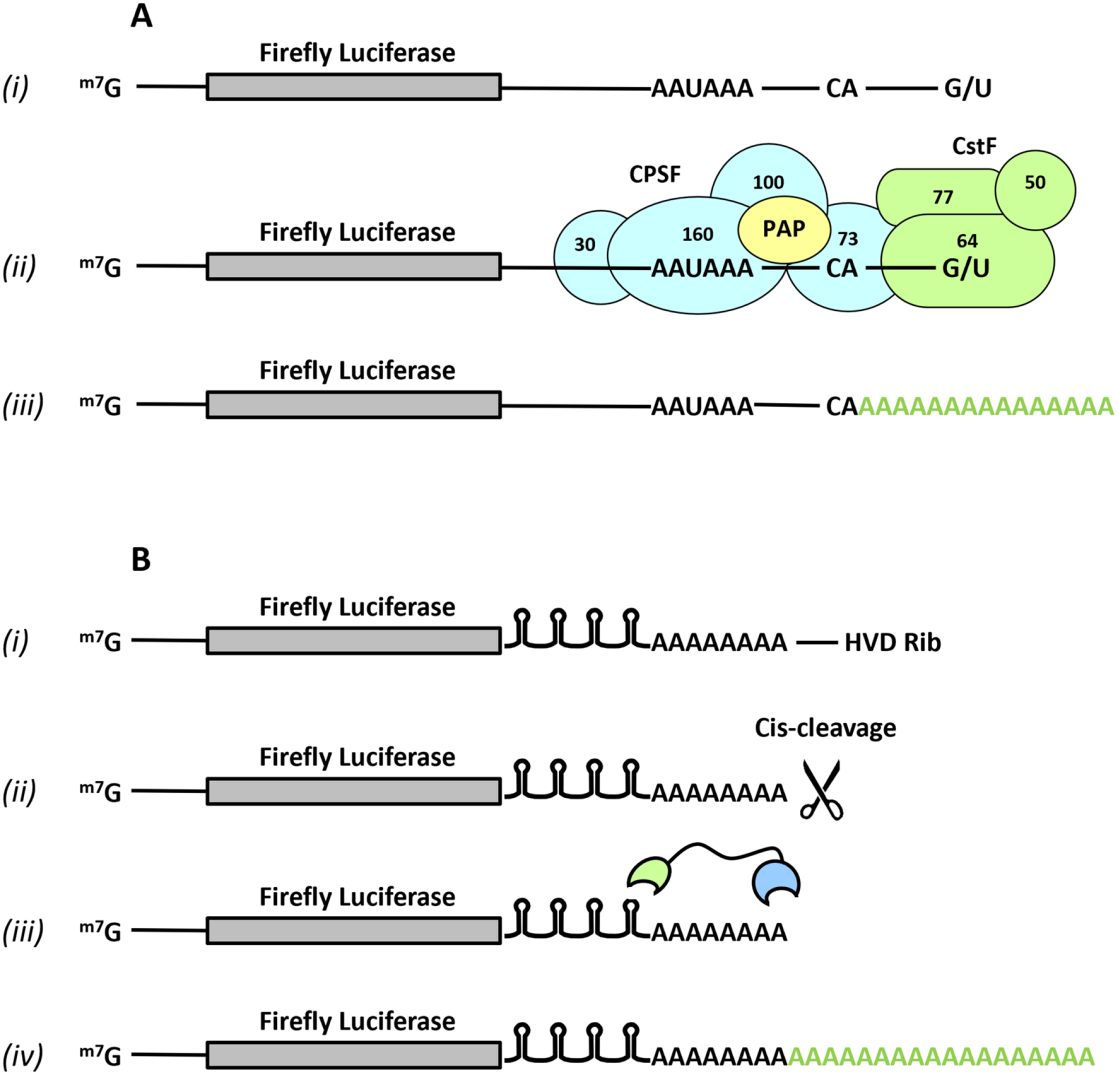
Synthetic view of the canonical nuclear polyadenylation and artificial polyadenylation system. (**A**) Standard nuclear polyadenylation. *(i)* Various sequences located in the 3′ region are involved in the nuclear cleavage and polyadenylation of pre-mRNAs, including *(ii)* a polyadenylation signal (PAS), commonly AAUAAA hexamer in metazoans, which is bound by the 160 kDa subunit of large multimeric Cleavage and Polyadenylation Specificity Factor complex (CPSF160). In metazoans, the CA dinucleotide, which is the site of endonucleotic cleavage of the pre-mRNA by the 73 kDa subunit of CPSF (CPSF73), is located approximately 19 nucleotides downstream of PAS. The Cleavage-stimulation Factor (CstF) is a heterotrimeric complex recruited by CPSF, whose 64 kDa subunit (CstF64) recognizes the G/U-rich motif downstream of PAS on the pre-mRNA. *(iii)* After cleavage, the poly(A) tail of mRNA is extended by the nuclear poly(A) polymerases bound to the CPSF complex. (**B**) General view of the artificial polyadenylation system for transcripts synthesized by C3P3-G1, with which (*i*) the C3P3-G1 enzyme synthesizes mRNA whose 5′-end is ^m7^GpppN-capped and whose 3′-end contains a poly(A) sequence of 40 adenosine, followed by a cis-splicing ribozyme of the virus of hepatitis D; (*ii*) self-cleavage of the Firefly luciferase reporter mRNA immediately after poly(A) track by hepatitis D virus genomic ribozyme *(iii)* four BoxBr tethering hairpins from the λ virus are introduced in 3′UTR region of reporter mRNA, which bind at high affinity the N peptide from λ virus (green oval). This peptide sequence is fused to the poly(A) polymerases (blue oval) through a flexible linker; (*iv*) The resulting chimeric poly(A) polymerases, which are recruited at the 3′UTR of Firefly luciferase reporter mRNA, elongate the poly(A) tail post-transcriptionally.

Polyadenylation is a post-transcriptional modification found at the 3′-end of virtually all eukaryotic mRNAs, with the notable exception of most histone mRNAs, which have a stem-loop at their 3′-end and produces cleaved non-polyadenylated mature mRNAs, although a subset of replication-dependent histone mRNAs can be also expressed as polyadenylated mRNA^6,7^. Polyadenylation occurs post-transcriptionally by poly(A) polymerases (PAP), whose prototype is the nuclear poly(A) polymerase alpha (PAPα). This enzyme post-transcriptionally adds residues of adenosine monophosphate of adenosine triphosphate to RNA by releasing a pyrophosphate group. PAPα is part of a protein complex with cleavage and polyadenylation specificity factor (CPSF), which binds to the AAUAAA polyadenylation signal hexamer, and cleaves pre-mRNA 12–30 nucleotides downstream to the hexamer. The polyadenylation complex also includes the cleavage stimulation factor F (CstF), which binds to the G/U-rich signal and cleaves the 3′-most part of a newly produced RNA (Fig. 1A). Once synthesized, the polyadenylated tail plays a crucial role in regulating the stability, nuclear transport, and translation of mRNAs^8–12^.

Given the importance of poly(A) tails in mRNA translation, we sought to extend the length of the poly(A) tails generated by the C3P3-G1 system and evaluate benefits in protein expression levels. The original C3P3-G1 system produced a short 40-residue poly(A) tail on mRNAs, transcribed from an adenosine track on the DNA template, followed by a cis-cleaving hepatitis D virus ribozyme. Conversely, native mRNAs contain poly(A) tails of up to 250–300 nucleotides at synthesis, then shortened to varying lengths in the cytoplasm with a median between 50 and 100 nucleotides at steady state^13–18^. Given the role of polyadenylation in eukaryotes on mRNA processing, we thought of developing a second-generation C3P3 system (C3P3-G2) that generates significantly longer poly(A) tails on C3P3 transcripts.

To extend poly(A) tails in the C3P3 system, a chimeric mutant PAPα was tethered to the 3′-untranslated region (3′UTR) of target mRNA. This modification post-transcriptionally lengthened the poly(A) tail of mRNA synthesized by the C3P3-G1 enzyme (Fig. 1B), and increased expression of Firefly luciferase from mRNAs by nearly 3-times compared to the first-generation C3P3-G1 system. The development of a polyadenylation system is therefore an important milestone in the improvement of exogenous gene expression by DNA-based artificial cytoplasmic expression systems.

## Material and methods

### Plasmids

Artificial gene sequences were synthesized and assembled from stepwise PCR using oligonucleotides, cloned and fully sequence verified by GeneArt AG (Regensburg, Germany). The coding sequences of all constructions were optimized for protein expression in human cells with respect to codon adaptation index^19^.

The candidate poly(A) polymerase plasmids consisted of the IE1 promoter/enhancer from the human cytomegalovirus (CMV), 5′UTR from human β-globin, Kozak consensus sequence followed by the open-reading frame (ORF) of tethered poly(A) polymerases, 3′UTR from human β-globin, and SV40 polyadenylation signal (Supplementary Fig. 1). Unless otherwise indicated, poly(A) polymerases were tethered by the fusion at their amino-terminal ends with the N-peptide sequence of 21 amino acids from the λ virus.

The C3P3-G1 plasmid, also named pCMV-NP868R-(G_4_S)_2_-K1ERNAP(R551S), was previously described^3^. It contains a single open-reading frame (ORF) encoding for the NP868R capping enzyme of the African Swine fever virus fused through a flexible (G4S)_2_ linker to the mutant DNA-dependent RNA polymerase from the K1E bacteriophage (Supplementary Fig. 2).

The Firefly luciferase reporter plasmid pK1E-Luciferase-4xλBoxBr-A40, which was used for the cell assays unless otherwise indicated, consists of the K1E phage RNA polymerase promoter, 5′UTR from human β-globin, Kozak consensus sequence followed by the ORF of the Firefly luciferase gene from *Photinus pyralis*, 3′UTR from human β-globin, four BoxBr of 17 nucleotides in tandem from the λ-virus, poly(A) track of 40 adenosine, self-cleaving hepatitis D virus antigenomic ribozyme sequence, and terminated by the bacteriophage T7 φ10 transcription stop (Supplementary Fig. 3).

### Cell culture and transfection

For standard experiments, the Human Embryonic Kidney 293 (HEK-293, ATCC CRL 1573) cells were routinely grown at 37 °C in 5% CO_2_ atmosphere at 100% relative humidity. Cells were maintained in Dulbecco’s Modified Eagle’s Medium (DMEM) supplemented with 4 mM l-alanyl-l-glutamine, 10% fetal bovine serum (FBS), 1% non-essential amino-acids, 1% sodium pyruvate, 1% penicillin and streptomycin, and 0.25% fungizone.

Cells were routinely plated in 24-well plates at 1 × 10^5^ cells per well the day before transfection and transfected at 80% cell confluence. Transient transfection was performed with Lipofectamine 2000 reagent (Invitrogen, Carlsbad, CA) according to manufacturer’s recommendations, except when otherwise stated. Lipoplexes were prepared by mixing plasmid DNA (μg) with Lipofectamine 2000 (μl) in a ratio of 1:2.5. For standard luciferase and hSEAP gene reporter expression assays, cells were analyzed 48 h after transfection, unless otherwise indicated.

### Firefly luciferase oxidation assays and eSEAP gene reporter measurements

Luciferase luminescence was assayed by the Luciferase Assay System (Promega, Madison, WI) as described elsewhere with minor modifications^3^. In brief, cells were lysed with 200 µL of Cell Culture Lysis Reagent buffer (CLR, Promega). Cell lysates were transferred to microcentrifuge tubes, briefly vortexed, then centrifuged at 12,000×*g* for two minutes at 4 °C. The supernatant was transferred to a microplate (20 µL/well), then Luciferase Assay Reagent (Promega; 100 µL/well) diluted at 1:10 was added to protein extracts. Luminescence readout was taken on a Tristar 2 microplate reader (Berthold, Bad Wildbad, Germany) with a read time of one second per well. For translatability assays, the area-under-curve from D0-to-D6 (AUC_D0–D6_) of the Firefly luminescence was calculated using the linear trapezoid method.

In order to normalize for transfection efficacy, cells were transfected with the pORF-eSEAP plasmid (InvivoGen, San Diego, CA), which encodes for the human secreted embryonic alkaline phosphatase driven by the EF-1α/HTLV composite promoter. Enzymatic activity was assayed in cell culture medium using the Quanti-Blue colorimetric enzyme assay kit (InvivoGen) as described elsewhere^3^. Gene reporter expression was expressed as the ratio of luciferase luminescence (RLU; relative light units) to eSEAP absorbance (OD, optic density).

### siRNA-mediated gene knockdown

HEK-293 cells were transfected at the final concentration of 100 nM with chemically synthesized pools of four 21-nucleotide siRNAs against human cyclin B and human p34^cdc2^ (Supplementary Material and Data 1), or non-targeting pool of siRNA used as a negative control (Dharmacon, Lafayette, CO)^20^.

### Cell proliferation and cytotoxicity assays

Cell viability was measured with the CyQUANT LDH Cytotoxicity Assay Kit (Invitrogen), a colorimetric assay to measure lactate dehydrogenase (LDH), which is a cytoplasmic enzyme released from dead or dying cells into the cell culture medium^21^. The assay is based on the conversion by LDH of lactate to pyruvate through the reduction of NAD+ to NADH, which reduces a tetrazolium salt to a red formazan product that can be measured on a microplate reader. Cytotoxicity assay was performed according to the manufacturer's instructions by adding the reaction mixture to the cell culture medium, followed 30 min later by measuring the difference between the absorbance values at 490 nm and that at 680 nm measured with a monochrometer-based Tecan instrument (Männedorf Switzerland).

Cell proliferation was assayed using the CyQUANT Direct Cell Proliferation kit (Invitrogen), which consists of two components, the green cyanine dye and a background suppression dye. This assay is based on a nucleic acid stain is a live cell permeable reagent that mainly binds to the nuclear DNA of mammalian cells^22^, whereas the suppression dye is impermeable in live cells and suppresses green fluorescence. The assay was performed according to the manufacturer's instructions by adding the reaction mixture to the cultured cells, followed 60 min later by measuring the fluorescence at 508 nm excitation and 527 nm emission wavelengths using a monochrometer-based Tecan instrument.

### Flow cytometry analysis

Transfection rate and protein expression levels were assayed with the Guava easyCyte flow cytometer (Luminex, Austin, TX) in HEK-293 cells transfected with eGFP plasmid under control of the C3P3 promoter or a conventional nuclear CMV enhancer/promoter.

### Western-blotting

For C3P3-G1 or Nλ-PAPαm7 Western-blotting, one 3xFLAG-tag was introduced in-frame into the ORF the enzymes^23^. For the Western-blot of C3P3-G2, the 3xFLAG-tagged Nλ-PAPαm7 sequence was fused in-frame with the 3xFLAG-tagged C3P3-G1 enzyme through an F2A ribosome skipping sequence. Western blotting was carried out essentially as described elsewhere, with minor modifications^3^. Cells were transfected as previously described, lysed in 200 µl of CLR buffer, then lysate was clarified by spinning for 15 s at 12,000×*g* at room temperature. Forty micrograms of total protein were resolved on 7% polyacrylamide gel, and transferred onto nitrocellulose Hybond membrane (GE Healthcare, Pittsburgh, PA) overnight at + 4 °C. Membranes were blocked with 5% skim milk powder in Tris Buffered Saline with Tween 20. Membranes were incubated with the rabbit polyclonal F7425 anti-FLAG primary antibody (1:1000; Sigma-Aldrich, Saint-Louis, MO) for 1 h at room temperature, then incubated with the anti-rabbit IgG-conjugated horseradish peroxidase 7074 antibody (1:1000; Cell Signaling Technologies, Danvers, MA). The membrane was submerged with SuperSignal West Pico Chemiluminescent Substrate solution (ThermoFisher Scientific, Waltham, MA) and scanned with the Amersham Biosciences Imager 600 (GE Healthcare, Chicago, IL). Molecular weights were determined using the high range color-coded prestained protein markers (Cell Signaling Technologies, Danvers, MA). Membranes were analyzed with ImageJ software^24^.

### C3P3 protein fluorescence imaging by laser-scanning confocal microscopy

Each moiety of the C3P3-G2 enzyme, which is obtained by fusion into a single ORF of the Nλ-PAPαm7 sequence with the C3P3-G1 enzyme via an F2A ribosome skipping sequence, were imaged by indirect immunofluorescence. To this end, one 3xFLAG tag was fused in frame to the carboxyl-terminal end of the Nλ-PAPαm7 subunit, while a 3xV5 tag to the amino-terminal ends of C3P3-G1^25^. Chinese Hamster Ovary K1 (CHO-K1, ATCC CCL-61) were transfected as described above, then fixed in 4% paraformaldehyde for 15 min at room temperature, washed in PBS and permeabilized for 10 min in 0.1% Triton X-100. Non-specific-binding was blocked with 5% normal serum (v/v) in 3% PBS-BSA buffer. Cells were incubated with the rabbit F7425 polyclonal IgG anti-3xFLAG antibody (1:100; Sigma-Aldrich, Saint-Louis, MO) and the mouse R960-25 anti-V5 Tag monoclonal IgG2a antibody (1:250; Thermo-Fisher). Cells were then incubated with the secondary Alexa Fluor 568-conjugated goat anti-rabbit A11036 orange-fluorescent (1:2000) and the Alexa Fluor 488-conjugated goat anti-mouse A11029 green-fluorescent antibody (1:500; Thermo-Fisher). Slides were mounted in the anti-fade Vectashield Mounting Medium (Vector Laboratories, Burlingame, CA), then imaged on a Leica SP8 confocal microscope equipped with the appropriate laser excitation filters under oil immersion with image magnification and processed with Leica LAS-AF software. For nuclear stained cell imaging, slides were incubated with Hoechst 33342 dye.

### Real-time mRNA synthesis imaging

Real-time live cell production of mRNA by the C3P3 system was performed with SmartFlare probes (Merck-Millipore, Billerica, MA). These probes are made of a gold nanoparticle conjugated to multiple copies of double-stranded oligonucleotide consisting of capture mRNA-specific complementary sequences hybridized to complementary reporter sequences^26^. The capture sequences are bound to gold nanoparticles, while the 5′-ends of complementary reporter sequences are bound to Cy-5 fluorophore. The fluorescence of the reporter sequences is quenched by its proximity to the gold core. When the target cellular mRNA is present, it hybridizes with the capture sequences linked to the gold nanoparticles, which releases the released reporter strand, which is no longer quenched and becomes fluorescent. CHO-K1 cells were plated on Lab-Tek chambered cover glasses and co-transfected as described above with C3P3-G1 plasmid, together with a plasmid containing the gene of interest under control of the C3P3 promoter. The SmartFlare probe was added to the culture medium at a final concentration of 400 pM and placed in a thermoregulated flow chamber at 37 °C in a humidified atmosphere carrying 5% CO_2_ using a microscope video camera (Zeiss, Iena, Germany). Cells were continuously imaged every five minutes for 48 h using the 650 nm laser excitation filters. Images were analyzed with the Zen Blue 2012 software (Zeiss). For nuclear staining, Hoechst 33342 was added to the culture medium and imaged as described above.

### mRNA translatability measurement

The effect of lengthening poly(A) tail on kinetics of target transcripts synthesized by the C3P3 system was assessed by quantitative reverse transcription-polymerase chain reaction (RT-qPCR) as described elsewhere with minor modifications^3^. HEK-293 cells were transfected as described above, then total RNA was isolated using the Nucleospin RNA columns (Macherey–Nagel, Düren, Germany) and subjected to TURBO DNase treatment (Ambion, Foster City, CA). RNA samples were quantified using a Nanodrop One spectrophotometer (ThermoFisher Scientific) and their integrity was assessed using the Agilent 2100 Bioanalyzer with the RNA 6000 Nano Kit (Agilent Technologies). Total RNA was reverse-transcribed using the high-capacity cDNA reverse transcription kit with RNase inhibitor (Life Technologies). The cDNA was then amplified by real-time RT-qPCR using primer and MGB probe sets for the Firefly luciferase and C3P3-G1 mRNAs with TaqMan detection (Supplementary Material and Data). All measured RIN values for the samples were greater than 9. Copy number quantification relied on the following normalization steps (Supplementary Material and Data). First, the purified RNAs were quantified by nanodrop and by Bioanalyzer, which made it possible to carry out the reverse transcription under similar conditions for all the samples. Second, glyceraldehyde-3-phosphate dehydrogenase (GAPDH) and β-actin (ACTB) mRNAs were quantified by RT-qPCR simultaneously with the mRNA of interest. The values obtained by RT-qPCR were thus corrected by the possible variations of these two reference genes in each of the samples. Third, the exact determination of the copy number is made by using a reference plasmid carrying all the amplicon sequences of the target genes, which makes it possible to correlate copy number and Ct, and thereby the copy number in the initial sample. For the quantification of translatability, the AUC_D0-D6_ of the Firefly luciferase mRNA was calculated using the linear trapezoid method, then reported to the AUC_D0-D6_ of the Firefly luciferase quantified by luciferin oxidation and also calculated by the linear trapezoid method.

### Poly(A) tailing assay

The poly(A) tail length of C3P3 transcripts was investigated using the Poly(A) Tailing Assay (also named RACE-PAT), which is an application of the 3′-RACE^27^. In brief, HEK-293 cells were co-transfected with the C3P3-G1 plasmid and pK1E-Luciferase-4xλBoxBr-A40, with or without the Nλ-PAPαm7 plasmid. Total RNA was then extracted with Nucleospin RNA columns as previously described, followed by incubation with the yeast poly(A) polymerase and GTP, which adds a limited number of guanosine and inosine nucleotides to the 3′-ends of poly(A)-containing RNAs, thus creating a unique poly(A)-oligo(G) junction (Affymetrix, Cleveland, OH). The tailed-RNAs was then converted to cDNA through reverse transcription using the newly added G/I tails as the priming sites. The resulting cDNA was hot-start PCR amplified using one of the two gene-specific forward primers (Supplementary Material and Data 4) and the universal 35-mers reverse primer that includes the poly(A) tails of the gene-of-interest. Finally, the PCR products are separated on an 2.5% agarose gel and images were analyzed with the ImageJ software. The length of the poly(A) tail was calculated as the sizes of poly(A) PCR-amplified products minus the calculated length of the gene-specific forward primer to the start of the templated poly(A) sequence.

### Bioinformatics

Prediction of p34^cdc2^ kinase phosphorylation sites was carried with the NetPhos 3.1 algorithm, which identifies candidate serine, threonine or tyrosine phosphorylation sites in eukaryotic proteins using ensembles of neural networks^28^.

For phylogenetic tree of the poly(A) polymerases, sequences were aligned by the ClustalW algorithm using a BLOSUM matrix (BLOcks Substitution Matrix) and default alignment parameters^29^. The phylogenetic tree was then drawn using FigTree software, using the distance-based neighbor-join method (http://tree.bio.ed.ac.uk/software/figtree/).

### Statistics

The data analysis was carried out with GraphPad Prism software (version 8.4.3, GraphPad Software for Science Inc., San Diego, CA). Comparison between two groups was performed by two-tailed Student’s t-test of two groups or one-way ANOVA, adjusted by Dunnett’s Post Hoc Test to compare the between means of more than two groups. Results are means (n ≥ 4) ± standard deviation. Significant differences are indicated by asterisks (*P < 0.05; **P < 0.01; ***P < 0.001; ****P < 0.0001).

## Results

### C3P3-G1 is an artificial expression system that allows the autonomous synthesis of the mRNA of interest in the cytoplasmic compartment

The C3P3-G1 system relies on an artificial capping-prone DNA-dependent RNA polymerase that functions in the cytoplasmic compartment. Cytoplasmic expression circumvents the limiting dependence on access to the nucleus for exogenous gene expression. Furthermore, transcription by the artificial RNA polymerase C3P3 is under the control of the corresponding promoter sequence, while its cytoplasmic localization restricts illegitimate transcription of the nuclear genome of the host cell, as supported by Agilent oligonucleotide microarray assays (P. H. Jais, unpublished data).

We then focused on the kinetics of expression by the C3P3 system, which we studied by live-cell mRNA synthesis imaging using SmartFlare probes. These gold nanoparticles are conjugated to multiple copies of double-stranded Cy-5-tagged oligonucleotides that fluoresce upon hybridization with cellular target mRNA^30^. Time-lapse live-cell imaging showed that the fluorescent signal was detectable as clusters of fluorescent dots as early as eight hours after transfection and gradually increased until the end of recording 48 h later (Supplementary Video).

Altogether, these results confirm that the synthesis of the target mRNA by the C3P3-G1 system takes place in the cytoplasmic compartment, which is an essential parameter to take into account for the development of an artificial poly(A) tail elongation process, such as the one developed here.

### Several cytoplasmic PAPs from different kingdoms of life can significantly increase protein expression by the C3P3 system

Poly(A) tail on the 3′ end of mRNAs is important for mRNA stability and efficient translation initiation. In the C3P3-G1 system, a 40-adenosine residue poly(A) tail was added to mRNAs by incorporation of 40 adenosines into the DNA template followed by a trans-cleaving ribozyme from the hepatitis D virus. To develop a second generation C3P3 system that generates longer poly(A) tails to mRNAs synthesized by C3P3, we adapted the tethered function assay technique^31^. In this technique, the 3′UTR of the mRNA forms orthogonal non-covalent RNA–protein interaction. This technique has been used to decipher the role of proteins involved in the transport, localization or post-transcriptional processing of mRNA. Moreover, this technique was used to investigate the functioning of nuclear canonical poly(A) polymerase alpha (PAPα) and non-canonical cytoplasmic GLD-2 poly(A) polymerase^32,33^.

Among the well-established orthogonal RNA–protein interaction systems, we chose the system from the lambdoid bacteriophage family because it has nanomolar affinity between short peptide sequences of N anti-termination protein and specific hairpin nucleotide sequences^34^; the short sequences of both peptide and nucleotide were ideal for engineering into the C3P3 system. The tethering peptides consist of a 19–22 amino acid arginine-rich sequence found at the amino-terminus anti-terminator protein N from lambdoid bacteriophages. On the other hand, the tethered RNA sequences consist of 17–22 nucleotide leftward BoxBl and rightward BoxBr hairpins, in which 4 bases adopt a GNRA-like tetraloop structure, where N is any base and R is a purine^35^. We used this RNA–protein interaction system to tether various candidate poly(A) polymerases that catalyze the template-independent sequential addition of adenosine monophosphate units from ATP to the 3′-terminal hydroxyl groups of RNAs, releasing pyrophosphate.

**Figure 2:**
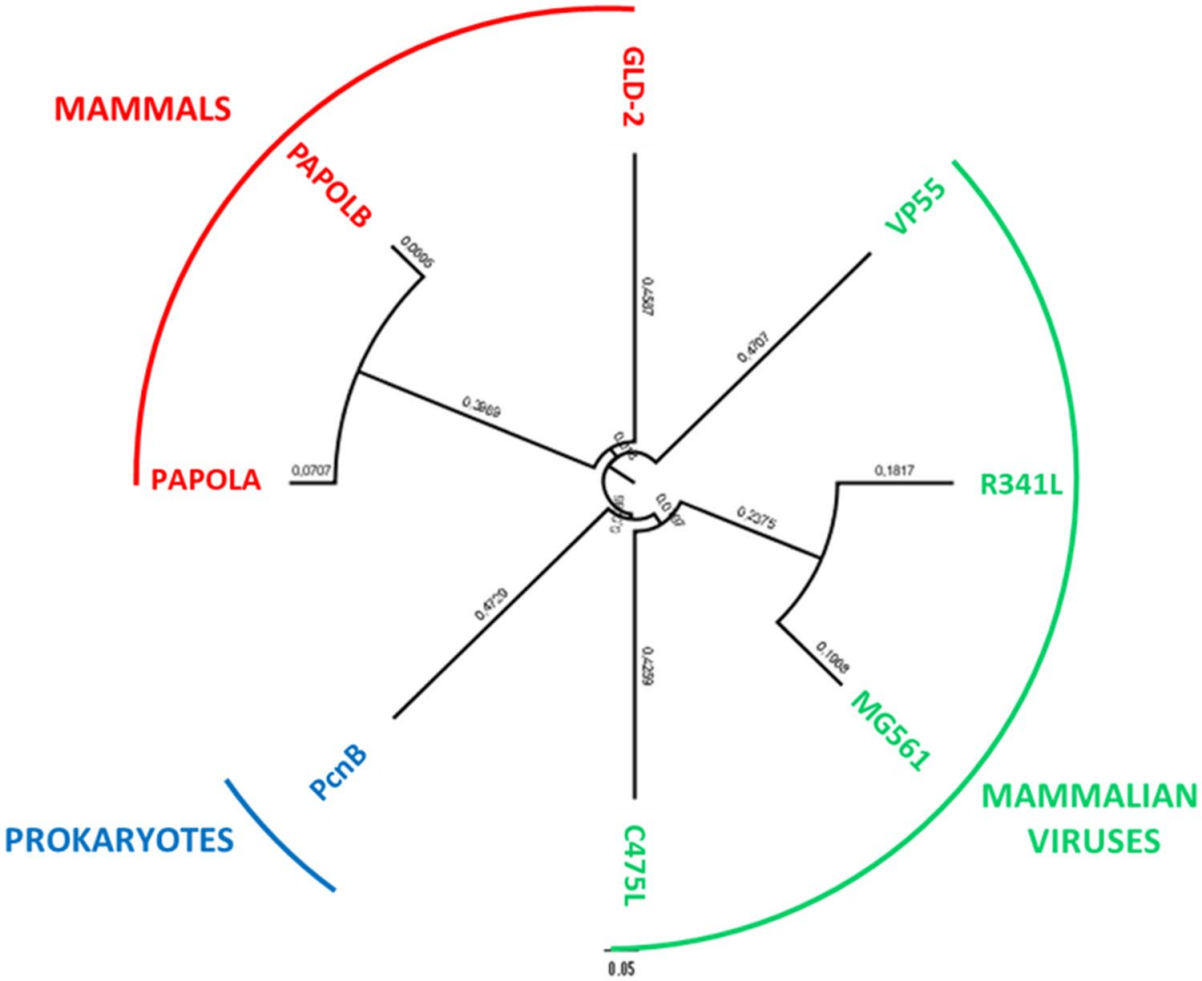
Phylogenetic tree of the poly(A) polymerases used in this work. The enzymes have been classified into three groups: 1°) those from eukaryotic DNA viruses, i.e. MG561 from Megavirus chiliensis, C475L from African swine fever virus, R341L from Acanthamoeba polyphaga mimivirus and VP55 from Vaccinia virus, 2°) enzymes from mouse as a representative of the animal kingdom, including the canonical poly(A) polymerase β (PAPβ, also named PAPOLB) and the non-canonical poly(A) polymerase GLD-2, 3°) PcnB poly(A) polymerase from Escherichia coli, as a representative of prokaryotes. Branch labels indicate posterior probability computed by the neighbor-joining method.

Poly(A) polymerases are found in various kingdoms of life. The phylogenetic tree of the poly(A) polymerases which will be tested below is depicted in Fig. 2. First, the canonical poly(A) polymerases from the eukaryotes, PAPα, β and γ, are mainly nuclear and have a tripartite structure as described hereinafter. Second, the non-canonical poly(A) polymerases from eukaryotic cells are essentially cytoplasmic and constitute a more heterogeneous group whose prototype is GLD-2. Third, poly(A) polymerases derived from eukaryotic viruses, in particular DNA viruses whose replication is cytoplasmic. Fourth, the poly(A) polymerases from prokaryotes, for which polyadenylation has a function radically different from that of eukaryotes because it is thought to act as a signal for transcriptional destabilization.

**Figure 3:**
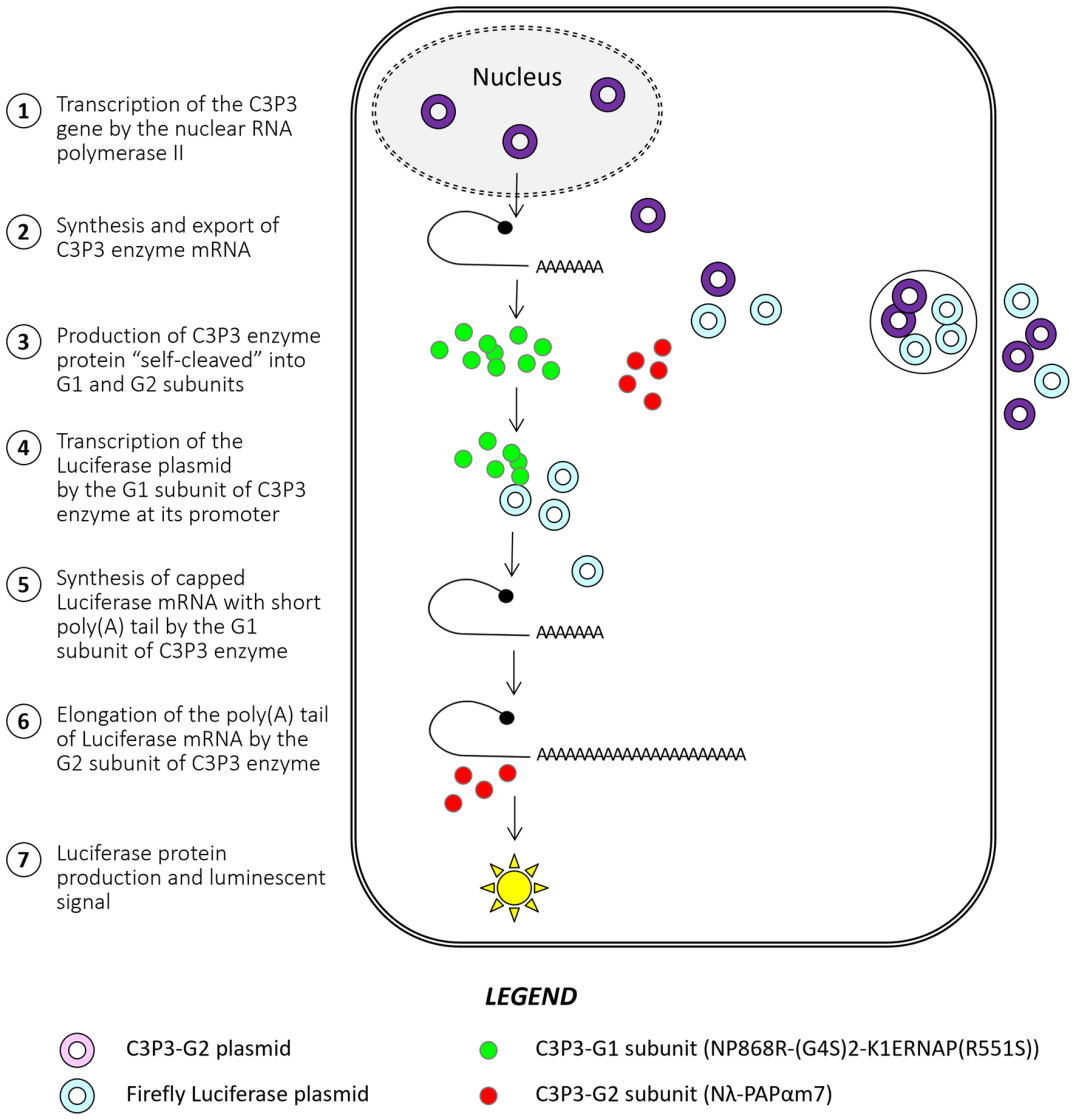
Process of target mRNA synthesis by the C3P3-G2 system. C3P3-G2 plasmid and the Firefly luciferase plasmid under the control of the C3P3 enzyme are co-transfected into HEK-293 cells (for simplification purposes, the final single ORF version of C3P3-G2 in a single plasmid is shown here). C3P3-G2 mRNA is expressed by an RNA polymerase II-dependent promoter, which is exported into the cytoplasmic compartment. C3P3-G2 mRNA is translated in the cytoplasm as a polyprotein that is “self-cleaved” into G1 and G2 subunits by F2A sequence. Both subunits are found the cytoplasm. The G1 subunit mediates both transcription and capping of Firefly luciferase mRNA. The G2 subunit then binds to the target mRNA through interaction between the Nλ peptide of the G2 subunit and the 4xBoxBr sequence in the 3′UTR of the target mRNA, resulting in elongation of the poly(A) tail. Finally, the Firefly luciferase protein is produced by translation of mRNA, whose enzymatic activity is monitored by a luciferin oxidation test.

To develop an artificial post-transcriptional polyadenylation system, we fused the amino-terminal ends of candidate poly(A) polymerases to Nλ-tethering peptide from the λ bacteriophage, while four tandem BoxBr RNA hairpin also from λ bacteriophage were introduced in the 3′UTR of the Firefly luciferase reporter gene. HEK-293 human cells were co-transfected with these plasmids together with the C3P3-G1 plasmid. Following expression of the C3P3-G1 enzyme, transcripts are synthesized and ^m7^GpppN-capped. Subsequently, the tethered poly(A) polymerases are recruited specifically to BoxBr from the target transcripts, which has the expected effect of lengthening their poly(A) tails. The effect of this post-transcriptional modification on reporter protein expression can be monitored by conventional luciferin oxidation assay (Fig. 3).

**Figure 4:**
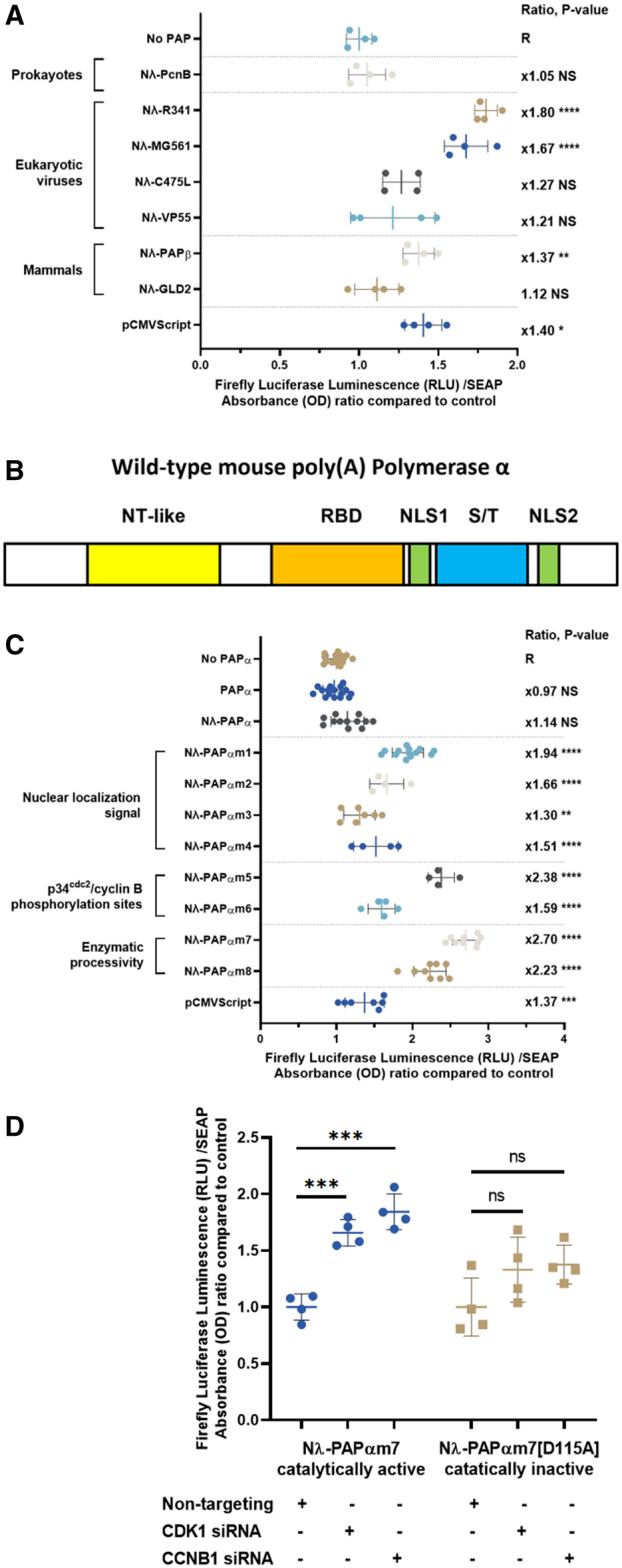
Screening phase of candidate tethered poly(A) polymerases. (**A**) First screening phase of candidate tethered poly(A) polymerases listed above. Plasmids encoding for the candidate tethered poly(A) polymerase plasmids were co-transfected in HEK-293 cells, together with the C3P3-G1 and Firefly luciferase reporter plasmids. Constructions were tested for luciferin oxidation assays 48 h after transfection and expressed as RLU divided by the hSEAP protein expression expressed in DO (RLU/DO ratio) and normalized to the reference group (R). Values represent the mean ± SD (n ≥ 4). Statistical analysis was performed by ANOVA adjusted by Dunnett’s Post Hoc analysis (*P < 0.05; **P < 0.01; ***P < 0.001; ****P < 0.0001). (**B**) Domain architecture of the wild-type mouse PAPα, which has a tripartite structure with a N-terminal nucleotidyl-transferase-like catalytic domain in yellow (NT-like), a central RNA binding domain (RBD) in orange, and highly phosphorylated Serine/Threonine enriched region (S/T) in blue. This S/T domain contains several p34^cdc2^/cyclin B phosphorylation sites involved in regulating enzymatic activity during the M-phase of the cell cycle. The two nuclear localization signals (NLS1 and NLS2) surround the serine/threonine rich region are shown in green. (**C**) Molecular engineering of the mouse tethered poly(A) polymerase α (PAPα), which was engineered by iterative targeted mutagenesis for enhanced reporter protein expression. Three series of PAPα mutants were successively tested. First series of mutations intended to relocate PAPα in the cytoplasm. The mutant Nλ-PAPαm1, which impair the SUMOylation site of NLS2, gave the best results and was used for further optimization. Second series of mutations designed to inactivate phosphorylation sites in the serine/threonine-rich region of mouse PAPα by p34^cdc2^/cyclin B. Nλ-PAPαm5 mutant, which comprises three inactivating mutations of consensus sites conserved between murine and bovine species, gave the best results. Third series of mutations with increased processivity in vitro. The mutant Nλ-PAPαm7, which comprises an F100I substitution, gave the best results and was used as the final C3P3-G2 enzyme. Constructions were tested for luciferin oxidation assays 48 h after transfection and expressed as RLU divided by the hSEAP protein expression expressed in DO (RLU/DO ratio) and normalized to the reference group (R). Values represent the mean ± SD (n ≥ 4). Statistical analysis was performed by ANOVA adjusted by Dunnett’s Post Hoc analysis for multiple comparisons (*P < 0.05; **P < 0.01; ***P < 0.001; ****P < 0.0001). (**E**) siRNA knockdown of human cyclin B (CCNB1) and p34^cdc2^ (CDK1). HEK-293 cell assays were performed as previously described and co-transfected with pools of siRNA at a concentration of 100 nM. Luciferin oxidation assays was performed 48 h after transfection and expressed as RLU normalized for hSEAP protein expression expressed in DO (RLU/DO ratio) relative to reference condition (i.e. pool of non-targeting siRNA, empty bars). Values represent the mean ± SD (n ≥ 4). Statistical analysis was performed by ANOVA adjusted by Dunnett’s Post Hoc analysis for multiple comparisons (*P < 0.05; **P < 0.01; ***P < 0.001; ****P < 0.0001).

In a first screening step, we evaluated several wild-type poly(A) polymerases from eukaryotic viruses or mesophilic prokaryotes, which were selected for their predicted cytoplasmic localization, as well as for their optimum temperature close to 37 °C. Four of these poly(A) polymerases were from cytoplasmic DNA viruses, i.e. MG561 from Megavirus chiliensis^36^, C475L from African swine fever virus^37^, R341L from Acanthamoeba polyphaga mimivirus^36^ and VP55 from Vaccinia virus^38^. In addition, the prokaryotic PcnB poly(A) polymerase from Escherichia coli was tested^39^. These enzymes were tethered to the Nλ-peptide as described above. All the poly(A) polymerases increased the protein expression of the reporter gene, the best results being obtained with the R341 and MG561 poly(A) polymerases which respectively increased the expression levels of the reporter protein by 1.80 and 1.67-fold respectively (P < 0.001; Fig. 4A, first and second series).

We also included in the initial screening two wild-type mammalian cytoplasmic poly(A) polymerases, which are found at least partially in the cytoplasmic compartment. First, the mouse PAPβ (also named PAPOLB) which is specifically expressed in testicular cells, where it lengthens the poly(A) tail of certain mRNAs^40^. Secondly, the cytoplasmic non-canonical mouse GLD-2 poly(A) polymerase, which associates with the GLD-3 regulatory subunit to form a heterodimer and is mostly expressed in the central nervous system^32^. These two enzymes were tethered with the Nλ peptide and tested as described above. Mouse tethered PAPβ enhanced significantly the expression levels of the Firefly luciferase reporter protein by 1.37-fold, but surprisingly, the tethered mouse cytoplasmic GLD2 poly(A) polymerase had nearly no detectable activity (P < 0.001 and NS, respectively; Fig. 4A, third series).

### PAPα increases protein expression by the C3P3 system when properly tethered and relocalized to the cytoplasm

Owing to encouraging results obtained with cytoplasmic poly(A) polymerases from various domains of life, we next evaluated if the same approach could also be developed to the canonical nuclear mammalian PAPα, which carries out the bulk of pre-mRNA polyadenylation in mammals. The mouse poly(A) polymerase α canonical isoform 1 (PAPα, also named PAPOLA) was chosen as a prototype because of its extensive structural and functional characterization enabling advanced engineering. As other canonical nuclear poly(A) polymerases, this enzyme has a tripartite structure (Fig. 4B): a nucleotidyl-transferase catalytic domain at the N-terminus, a central domain RNA binding region, and two C-terminal nuclear localization signals (NLS1 and NLS2) that surround a serine/threonine-rich region^41^. This C-terminal domain has several cyclin-dependent kinase phosphorylation sites, which finely regulate the enzymatic activity during the cell-division cycle^42^.

The C3P3 system being of cytoplasmic localization, the nuclear localization of the wild-type PAPα could represent a major obstacle to achieving high efficiency of polyadenylation in the cytoplasm^41^. To test if cytoplasmic localization of PAPα would enhance efficiency of cytoplasmic C3P3-based expression systems, one or both parts of the bipartite nuclear localization signal (NLS) were mutagenized. Four mutants were tested in this optimization step. First, in the mutant Nλ-PAPαm1, two lysine residues of NLS2 were substituted with arginine at residues at positions 656–657. This mutation inhibits the conjugation of PAPα by the protein SUMO 2/3 (Small Ubiquitin-like MOdifier), which results in relocation of PAPα to the cytoplasm^43^. Second, in the Nλ-PAPαm2 mutant, the bipartite NLS2 was inactivated and has been shown to relocate bovine PAPα to the cytoplasm^41^. Third, in the mutant Nλ-PAPαm3, the entire carboxyl-terminal end was deleted, which included the NLS1 and NLS2, as well as the interaction domain with cleavage and polyadenylation specificity factor (CPSF) that is involved in the cleavage of the signaling region from pre-mRNA at their 3′-end. This regulatory region, which was found to be non-essential for the in vitro activity of mammalian PAPα, is found exclusively in vertebrates and absent in protists^33,41^. Fourth, for Nλ-PAPαm4, four tandem nuclear export signals from HIV-1 were fused to the carboxy-terminus of PAPα in order to relocate PAPα to the cytoplasmic compartment^44^.

When biological activity of these mutant tethered PAPα were tested, as expected, the nuclear wild-type nuclear tethered PAPα had virtually no effect on the expression of the Firefly luciferase reporter protein. Conversely, all four tethered PAPα mutants increased the expression of the reporter protein at varying levels (Fig. 4C, first series). Of these, Nλ-PAPαm1 gave the best results by increasing by 1.94-fold the expression of Firefly luciferase reporter protein compared to C3P3-G1 system only (P < 0.001). The Nλ-PAPαm1 mutant was therefore selected for further enhancement, all mutations described below containing this mutation.

### Additional mutations of mouse PAPα that inactivate p34^cdc2^/cyclin phosphorylation and increase enzymatic processivity enhance protein expression by the C3P3 system

The activity of vertebrate PAPα is tightly regulated through post translational modifications, and such regulation could inactivate the activity of tethered PAPα during certain phases of the cell cycle. We have therefore investigated whether modifications to the post-translational regulation of the tethered PAPα could have a favorable effect on the protein expression levels by the C3P3 system. Specifically, native PAPα has a C-terminal serine/threonine-rich domain with several consensus (S/T-P-X-K/R) and non-consensus (S/T-P-X-X) cyclin-dependent kinase sites, which can be phosphorylated by p34^cdc2^/cyclin B during the M-phase of the cell cycle^45^. Such cell cycle-related phosphorylation downregulates PAPα enzyme activity and is thought to contribute to the reduction of mRNA polyadenylation during M phase^42^. We hypothesized that the phosphorylation of mouse Nλ-tethered PAPα by p34^cdc2^/cyclin B could reduce its enzymatic activity, and thereby decrease the polyadenylation lengthening of the target mRNA.

The role of PAPα phosphorylation by p34^cdc2^/cyclin B on luciferase expression were evaluated by co-transfecting siRNA pools against human cyclin B (CCNB1) or p34^cdc2^ (CDK1)_._ Both siRNA pools significantly increased protein expression compared to a pool of non-targeting siRNAs when co-transfected with the Nλ-PAPαm1 and C3P3-G1 plasmids (P < 0.001 for both siRNA; Fig. 4D). To demonstrate that the enhancement of luciferase expression upon p34^cdc2^/cyclin B silencing is specifically induced by tethered PAPα, the catalytically dead tethered PAPα was used under the same conditions. Co-transfection of siRNAs against human cyclin B or p34^cdc2^ did not significantly enhance expression of the Firefly luciferase reporter protein context of the catalytically dead tethered PAPα, thus confirming the specificity of the observed effect.

To engineer PAPα to be insensitive to p34^cdc2^/cyclin B, we generated Nλ-tethered PAPα with mutated p34^cdc2^/cyclin B phosphorylation sites. Specifically, alanine substitution by site-directed mutagenesis was conducted on validated and/or predicted phosphorylatable serine residues in the C-terminal region of the mouse PAPα (Fig. 4C, second series). Two mutants were tested in this optimization step. First, in Nλ-PAPαm5, alanine residues were substituted into the three serine residues conserved between murine and bovine PAPα and previously confirmed to have biological function on PAPα activity in vivo^46^. Secondly, in Nλ-PAPαm6, in addition to the above serine-to-alanine substitutions, four additional candidate phosphorylatable serine residues at non-consensus p34^cdc2^/cyclin B sites predicted by the NetPhos 3.1 neural network algorithm were inactivated by alanine substitutions^28^. Nλ-PAPαm5 was the most active mutant with 2.38-fold greater Firefly luciferase reporter protein expression compared to the C3P3-G1 system alone (P < 0.001), and was therefore selected for further optimization. In contrast, the Nλ-PAPαm6 mutant had much lower activity, which could possibly be explained by excessive structural modifications of the protein caused by additional mutations.

Finally with respect to optimizing PAPα, we evaluated whether mutations known to increase the processivity of the enzyme in vitro could also increase the level of protein expression by the C3P3 system (Fig. 4C, third series). Two mutants were tested in this final optimization step: Nλ-PAPαm7 and Nλ-PAPαm8, which correspond to F100I^41^ and R104A substitutions^47^, respectively. Both mutations, which are located in a α-helical helix, are proposed to widen the access channel to the substrate and thus promote the influx of ATP or the release of pyrophosphate (PPi) after formation of the phosphodiester bonds^47^. Nλ-PAPαm7 was the most active and increased expression of Firefly luciferase reporter protein 2.7-fold compared to no PAPα (P < 0.001). The Nλ-PAPαm7 enzyme was therefore selected as the final attached poly(A) polymerase, this term corresponding below to the enzyme used alone. Nλ-PAPαm7 is designated as C3P3-G2 when used as an independent module or assembled C3P3-G2 when fused to the C3P3-G1 enzyme in a single ORF.

### Optimal protein expression by the C3P3 system is found with at least four BoxBr hairpin repeats from the λ virus properly spaced in the 3′UTR of the target mRNA

Having optimized the poly(A) polymerase, the next goals was to establish the best we then optimized the RNA hairpin sequences to tether poly(A) polymerase to the 3′ UTR of mRNAs. The λ virus genome contains two hairpins BoxBl and BoxBr of 17 nucleotides which differ by only one nucleotide. The effects of the substitution of 4xλBoxBr from the λ virus by 4xλBoxBl repeats were tested, the latter having a slightly greater in vitro affinity than the former for the Nλ peptide^48^. No significant difference was observed between the BoxBr and BoxBl hairpins with canonical or extended stems with respect to Firefly luciferase reporter protein expression (Supplementary Fig. 4, first series).

With cis elements, the number of repeats can often affect the efficiency of recruiting trans elements. Accordingly, we evaluated the effect of varying the number of λBoxBr repeats on protein expression by the C3P3 system. From one-to-twelve λBoxBr repeats were introduced into the 3′UTR of the Firefly luciferase reporter gene and tested under the same conditions as before. A gradual increase in protein expression was observed up to four λBoxBr, after which a plateau was observed (Supplementary Fig. 4, second series), suggesting that four λBoxBr repeats are optimal.

Thirdly, we evaluated the effect of spacing between the λBoxBr hairpins, which we varied between 2 to 40 nucleotides. A marked decrease in protein expression was observed when the spacing between λBoxBr hairpins was reduced to just two nucleotides, which may be related to steric hindrance of Nλ-PAPαm7 binding to λ-BoxBr hairpins (Supplementary Fig. 4, third series). There was no virtually difference in Firefly luciferase activity when spacing was increased from 10 to 20 or 40 nucleotides.

### The integrity of the complete Nλ-PAPαm7/4xλBoxBr system is necessary for increased protein expression by the C3P3 system

In order to confirm the specificity of the post-transcriptional lengthening of the poly(A) tail, we synthesized an inactive Nλ-PAPαm7 mutant by D115A substitution. This mutation impairs the catalytic site of the poly(A) polymerase and thereby reduces the in vitro enzymatic activity of wild-type bovine PAPα to less than 1%^47^. The catalytically dead Nλ-PAPαm7[D115A], when used under the same conditions as above with the C3P3-G1 and pK1E-Luciferase-4xλBoxBr-A40 plasmids, led to no detectable change in Firefly luciferase reporter protein activity compared to the C3P3-G1 system alone, thus confirming the specificity of the observed effect (Supplementary Fig. 5A).

To further confirm the specificity of the Nλ-PAPαm7/4xλBoxBr system, the 4xλBoxBr hairpins were replaced with scrambled 4xscλBoxBr sequences. Again, no augmented luciferase activity was detected with the 4xscλBoxBr system compared to the C3P3-G1 system, thus confirming the importance of this sequence for the activity of the Nλ-PAPαm7/4xλBoxBr system (Supplementary Fig. 5B).

### The Nλ-PAPαm7/4xλBoxBr system can increase protein expression by the C3P3 system in various types of mammalian cells and with various genetic constructs

While the Nλ-PAPαm7/4xλBoxBr significantly augmented expression of luciferase, it was important to establish that similar enhancements would occur with other exogenous gene expression constructs, and hence would hold broad utility towards exogenous gene expression applications. To this end, we evaluated the effect of Nλ-PAPαm7/4xλBoxBr on expression of enhanced green fluorescent protein (eGFP). Specifically, eGFP was inserted into the pK1E-gene of interest-4xλBoxBr-A40 plasmid and co-transfected with C3P3-G1, plus-or-minus Nλ-PAPαm7. Levels of eGFP in transfected cells were quantified by flow cytometric analysis. eGFP expression was 3.7 times higher when the Nλ-PAPαm7 plasmid was co-transfected with the C3P3-G1 plasmid (Supplementary Fig. 6A). We then investigated whether the Nλ-PAPαm7/4xλBoxBr system could also increase the expression of other constructs with various 5′UTR or 3′UTR sequences. These different constructions were cotransfected as described above with the CP3-G1 plasmid, with or without the Nλ-PAPαm7 plasmid. A significant increase in expression was observed when the Nλ-PAPαm7 plasmid was co-transfected regardless of the 5′UTR or 3′UTR sequence (Supplementary Fig. 6B,C). Altogether, these findings suggest that the Nλ-PAPαm7/4xλBoxBr system can be utilized with broad exogenous genes and UTRs, so long as the major components such as the K1E promoter and 4xλBoxBr cis elements are present.

We then tested whether the Nλ-PAPαm7/4xλBoxBr system could be used in other mammalian cell types, which seems likely due to the orthogonal nature of the RNA–protein interaction system. Mouse NIH-3T3 fibroblasts, Chinese hamster ovary CHO-K1 cells, and rat K-9 hepatocytes were transfected under the same conditions as described previously (Supplementary Fig. 7A–C). In all cell lines, co-expression of Nλ-PAPαm7 significantly increased the level of the reporter protein by 2-to-fourfold (P < 0.001). These findings confirm that the Nλ-PAPαm7/4xλBoxBr system is functional in mammalian cultured cells other than human. Noticeably, the C3P3 system has poor efficiency in some cell lines compared to a standard nuclear expression plasmid (e.g. NIH-3T3 and K9 cells), unlike other cell lines where its performance is greater than a standard nuclear expression plasmid (e.g. CHO-K1 and HEK-293). The reasons for such difference remain unclear; their understanding constituting an important avenue for future improvement of the C3P3 system.

We next investigated whether there were differences in cell proliferation and cytotoxicity mediated by the C3P3 system that could explain the expression level differences observed between cell lines (Supplementary Fig. 8A,B). The rate of cell proliferation and cytotoxicity was measured in HEK-293 and CHO-K1 cells transfected with wild-type or enzymatically dead C3P3-G1, wild-type or enzymatically dead Nλ-PAPαm7, and pK1E-Luciferase-4xλBoxBr-A40 plasmids. The rates of cell proliferation and cytotoxicity were roughly similar in these two cell lines, with no statistically significant differences between the different experimental conditions compared to the reference. These results suggest that the C3P3-G1 and Nλ-PAPαm7 enzymes per se, as well as the Firefly luciferase mRNA produced by these systems, have no obvious specific effect on cell growth or death, although more subtle effects cannot be excluded. Cellular toxicity and decreased cell proliferation are, however, observed in these different experimental conditions compared to the transfection reagent alone, which are attributable to the plasmid DNA itself or to traces of endotoxin. A possible solution to reduce these nonspecific effects could therefore rely on either the establishment of stable cell lines that produce the C3P3 enzyme constitutively or inducibly, or on the use of cell lines modified by editing certain genes involved in the innate cellular immune response to exogenous DNA such as the Toll-like membrane receptor 9 for unmethylated CpG DNA^49^, or cyclic GMP-AMP synthase (cGAS)-STING pathway that senses DNA in the cytoplasm^50^.

### The Nλ/4xλBoxBr system compares favorably to other technological alternatives to polyadenylation

We then compared different technological alternatives that could have been used instead of or together with the present artificial polyadenylation system. First, in place of 3′-end mRNA polyadenylation, the metazoan replication-dependent histone mRNAs contain a conserved stem-loop sequence of 25–26 nucleotides, which binds to the nuclear stem-loop binding protein (SLBP). This complex is involved in all steps of histone mRNA metabolism, including processing, nuclear export, translation, and degradation^51^. Since the addition of a histone stem-loop downstream of a poly(A) track has been shown to potentiate the expression of synthetic mRNA vaccines^52^, we thought to compare such construct to the Nλ/4xλBoxBr system. The human H3 clustered histone 10 (H3C10) stem-loop was therefore introduced in the 3′UTR of the Firefly luciferase reporter plasmid with or without a 40-adenosine residue track. Tested under the same conditions as previously, the H3C10 stem-loop alone had almost the same effect as a 40-adenosine poly(A) track on the expression of the Firefly luciferase reporter gene (Supplementary Fig. 9A; ratio 1.06, NS). Moreover, an additive effect on reporter gene expression was observed when the H3C10 stem-loop was placed downstream of a 40-adenosine residue track (ratio 2.15; P < 0.001), but which was however significantly lower than that obtained with the Nλ/4xλBoxBr-A40 system (2.89-fold, P < 0.001). We finally tested the 3′UTR effect of an H3C10 stem-loop in a Firefly luciferase reporter plasmid containing a 4xλBoxBr-40 track. A marginal but non-significant increase in expression level was observed with this construct compared to a 4xλBoxBr-40 lane only (2.95 vs. 3.21-fold).

Several viruses have also developed functional replacement strategies for polyadenylation based on the use of highly structured 3′UTR sequences. For example, the 3′UTR of the S-segment of Bunyamwera orthobunyavirus^53^ and Andes hantavirus^54^ smRNA mediates efficient translation in the absence of a poly(A) tail through PABP-independent mechanisms, probably by forming a closed-loop mRNA by direct or indirect binding to eIF4G. Conversely, a conserved stem-loop from the non-polyadenylated 3′UTR of dengue virus type 2 mRNA ensures translation efficiency through its direct binding to PABP, thus forming a closed-loop mRNA^55^. These 3′UTR sequences were introduced upstream of the poly(A) track of the plasmid pK1E-Luciferase-A40, then were tested as described previously. A significant increase in the expression of the Firefly luciferase reporter gene was found with the Bunyamwera orthobunyavirus 3′UTR (ratio 1.66; P < 0.001), but much lower than that obtained with the Nλ/4xλBoxBr-A40 system (2.89-fold, P < 0.001; Supplementary Fig. 9B). No significant effect was observed with the Andes hantavirus or dengue virus 3′UTR sequences.

We also tested the effect of adding a 10-residue polycytosine track placed immediately downstream of the polyadenosine track (A40C10) of the pK1E-Luciferase-A40 plasmid. Such homopolymeric sequence was indeed found to increase and prolong the expression of synthetic mRNA both in vitro and in vivo, presumably by blocking its deadenylation^56^. Tested under the same conditions as previously in Firefly luciferase reporter gene construct without 4xλBoxBr repeat, a slight but non-significant increase in the expression of the Firefly luciferase reporter was indeed observed with the A40C10 compared to the A40 track (Supplementary Fig. 9C). Moreover, when A40C10 was inserted instead of A40 in a plasmid containing a 4xλBoxBr repeat, a slight but non-significant increase of expression in comparison to 4xλBoxBr-A10 was found (ratio of 2.77 and 2.95, respectively, P < 0.001).

### The Nλ-PAPαm7/4xλBoxBr system lengthens the poly(A) tail of the transcripts synthesized by the C3P3 system and increases mRNA translatability

**Figure 5:**
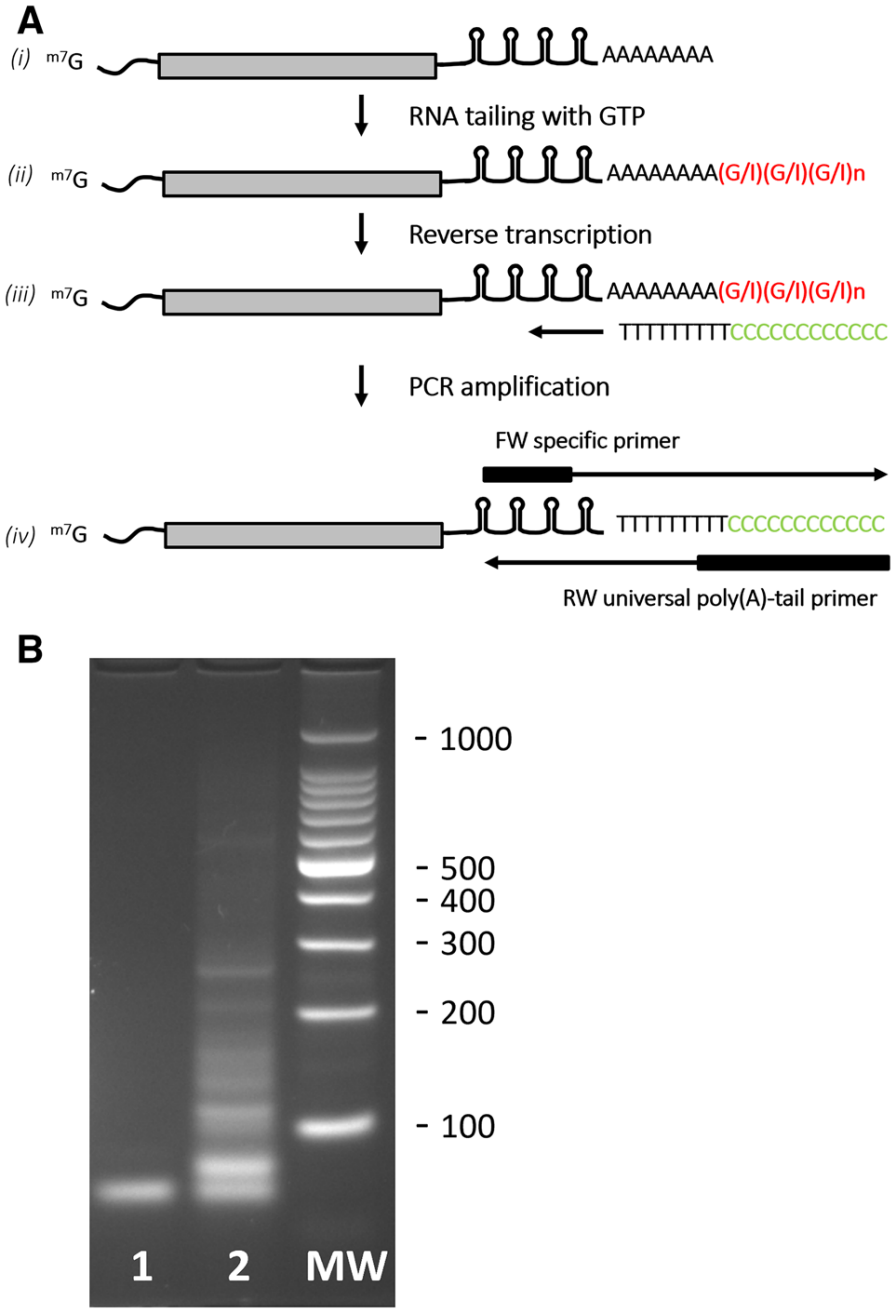
Poly(A) tailing assay. (**A**) Schematic presentation of the poly(A)-tailing assay adapted from^27^: (*i*) target mRNA with a poly(A) tail synthesized in vivo, (*ii*) poly(A) polymerase adds a limited number of guanosine and inosine nucleotides to its 3′-ends, (*iii*) the tailed-RNAs are reverse transcribed to cDNA using the newly added G/I tails and a universal primer, (*iv*) PCR amplification products are generated using a forward Firefly luciferase-specific primer and reward universal poly(A)-tail primer. (**B**) The resulting PCR products are run on 2.5% agarose gel: MW molecular weight markers, track 1: Firefly luciferase mRNA synthesized by C3P3-G1 enzyme only; track 2: Firefly luciferase mRNA synthesized by C3P3-G1 enzyme together with the tethered Nλ-PAPαm7 C3P3-G2 poly(A) polymerase.

To better appreciate the extent of poly(A) elongation on transcripts by the Nλ-PAPαm7/4xλBoxBr system, we used the poly(A)-tailing assay developed by Kusov et al.^27^. This PCR-based tailing method relies on the formation of a poly(A)-oligo(G) junction at the end of poly(A) tail of the target mRNA. The tailed-RNAs, converted to cDNA, is then amplified with a universal reward primer hybridizing at the poly(A)-oligo (G) junction and one of the two gene-specific forward primers tested (Fig. 5A). In the absence of Nλ-PAPαm7, a single band was mainly observed, corresponding to a poly(A) tail of 40 nucleotides (Fig. 5B; Supplementary Fig. 10, tracks 1). Conversely, a ladder of multiple bands was observed with Nλ-PAPαm7, therefore corresponding to a poly(A) tail of 40 to approximately 250 nucleotides (Fig. 5B; Supplementary Fig. 10, tracks 2). These findings therefore confirms that the Nλ-PAPαm7/4xλBoxBr system allows the elongation of the poly(A) tail of the transcripts synthesized with the C3P3 system.

**Figure 6:**
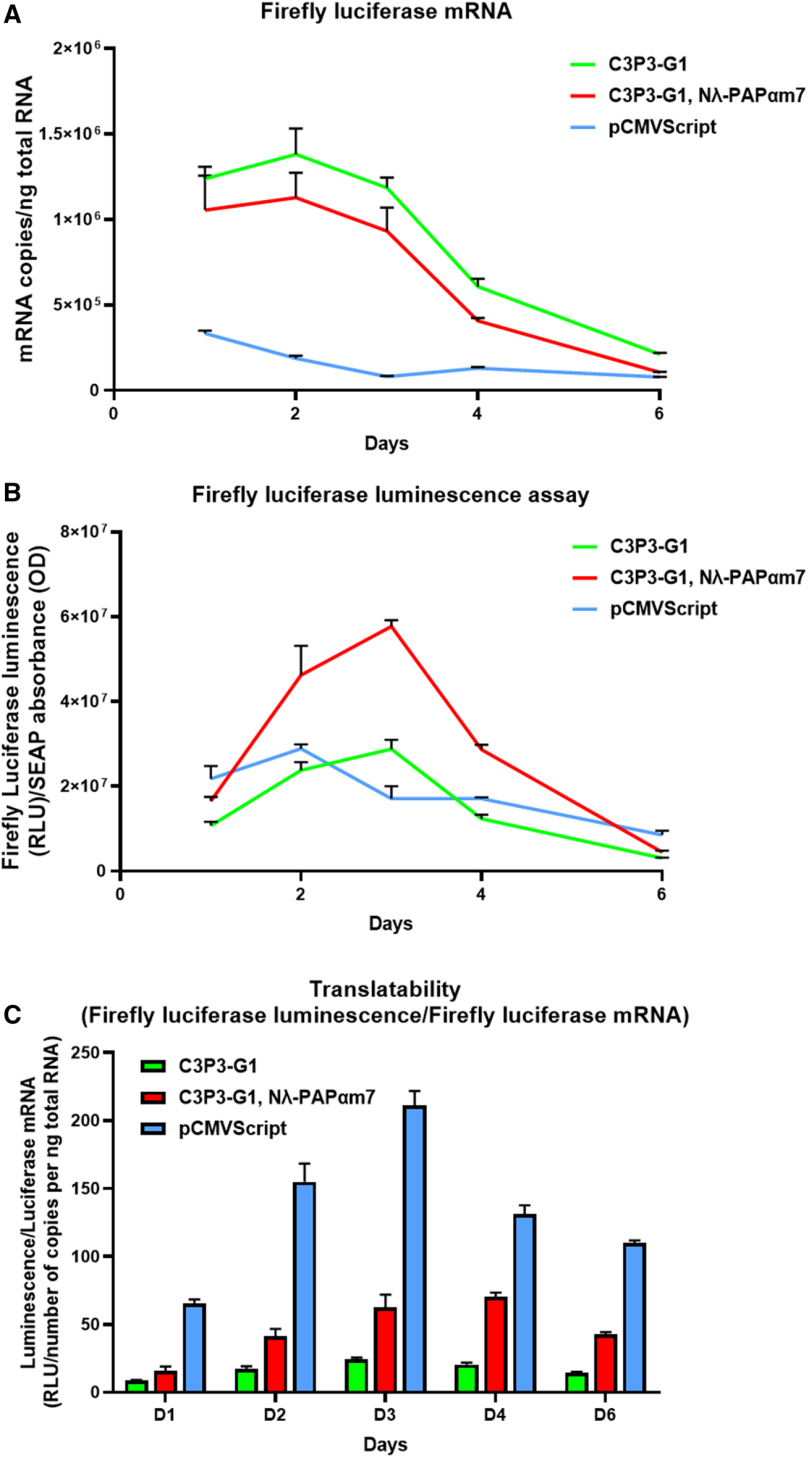
Firefly luciferase mRNA translatability assay. mRNA translatability was investigated by transfecting human HEK-293 cells either with the pCMVScript-Luciferase plasmid as a comparator, or with the C3P3-G1 plasmid with or without Nλ-PAPαm7 plasmid. (**A**) Kinetics of Firefly luciferase mRNA as number of copies per ng total RNA by RT-qPCR, and (**B**) Firefly luciferase luminescence expressed as Relative Light Units (RLU)/SEAP absorbance (OD), were used to calculate (**C**) the translatability index as the ratio of Firefly luciferase luminescence/Firefly luciferase mRNA.

We then focused on establishing the mechanisms that account for higher protein expression by the Nλ-PAPαm7/4xλBoxBr system. Poly(A) tails are well characterized to increase translatability by promoting the formation of closed loops. To investigate this hypothesis, we first measured by reverse transcription-quantitative PCR (RT-qPCR) the kinetics of the Firefly luciferase reporter mRNA synthesized by the C3P3 system with or without Nλ-PAPαm7/4xλBoxBr, and then compared it to that driven by the standard pCMVScript-Luciferase as a control. Firefly luciferase target reporter mRNA produced by C3P3-G1 with or without Nλ-PAPαm7 peaked at D2, unlike that produced by the pCMVScript which peaked at D1 (Fig. 6A). The C3P3-G1 plus Nλ-PAPαm7/4xλBoxBr system shows a slightly lower copy number of Firefly luciferase target mRNA than the C3P3-G1 system alone. On the other hand, Firefly luciferase AUC_D0–D6_ target mRNA copy number calculated by linear trapezoid method was 5.91 and 4.64 times higher with C3P3-G1 and C3P3-G1 plus Nλ-PAPαm7 than with the standard pCMVScript plasmid, respectively. The expression of Firefly luciferase protein in the transfected HEK-293 cells was simultaneously measured by luciferin oxidation assay. Firefly luciferase protein expression peaked at D3 in C3P3-G1 transfected cells with or without Nλ-PAPαm7, unlike the pCMVScript plasmid which peaked at D2 after transfection. The C3P3-G1 plus Nλ-PAPαm7/4xλBoxBr plasmid showed an AUC_D0–D6_ of Firefly luciferase bioluminescence 1.95 and 1.76 times higher than with the C3P3-G1 plasmid alone or the pCMVScript plasmid, respectively (Fig. 6B). Finally, the combination of the above results made it possible to calculate a translatability index, which we defined as the ratio AUC_D0-D6_ luminescence/AUC_D0-D6_ mRNA. This translatability index was 2.7 greater with the C3P3-G1 plus Nλ-PAPαm7 plasmids than with the C3P3-G1 plasmid alone (Fig. 6C). Nevertheless, compared to the standard pCMVScript expression plasmid, this translatability index was only 0.13 and 0.35 with respectively C3P3-G1 plasmid alone or together with the Nλ-PAPαm7 plasmid. These results therefore show that the PAPαm7/4xλBoxBr system clearly increases the translatability of mRNA synthesized by the C3P3 system, possibly by pseudo-circularization of the mRNA though the formation of closed-loops, but nevertheless there are still further advancements necessary to achieve the translatability levels exhibited by mRNA from CMV-driven nuclear expression.

To test the hypothesis that the Nλ-PAPαm7/4xBoxBr system enhances translatability through the formation of closed-loop with the poly(A) tail of the target mRNA, we evaluated the effect of proteins involved in such mRNA pseudo-circularization fused with Nλ tethering sequences, i.e. PABP which binds the poly(A) tail to mRNA 3′ ends, eIF4E which binds to the cap of mRNA 5′ ends and eIF4G which bridges these two proteins to form closed-loop mRNA. Tested under the same conditions as previously, expression of all three tethered proteins significantly increased Firefly luciferase reporter expression, although to a lesser degree than Nλ-PAPαm7, therefore supporting our initial hypothesis (Supplementary Fig. 11).

### The C3P3-G2 enzyme encoded by a single open reading frame can be efficiently assembled by fusing the tethered poly(A) polymerase Nλ-PAPαm7 with the C3P3-G1 enzyme NP868R-(G4S)2-K1ERNAP(R551S) via an F2A ribosome skipping sequence

**Figure 7:**
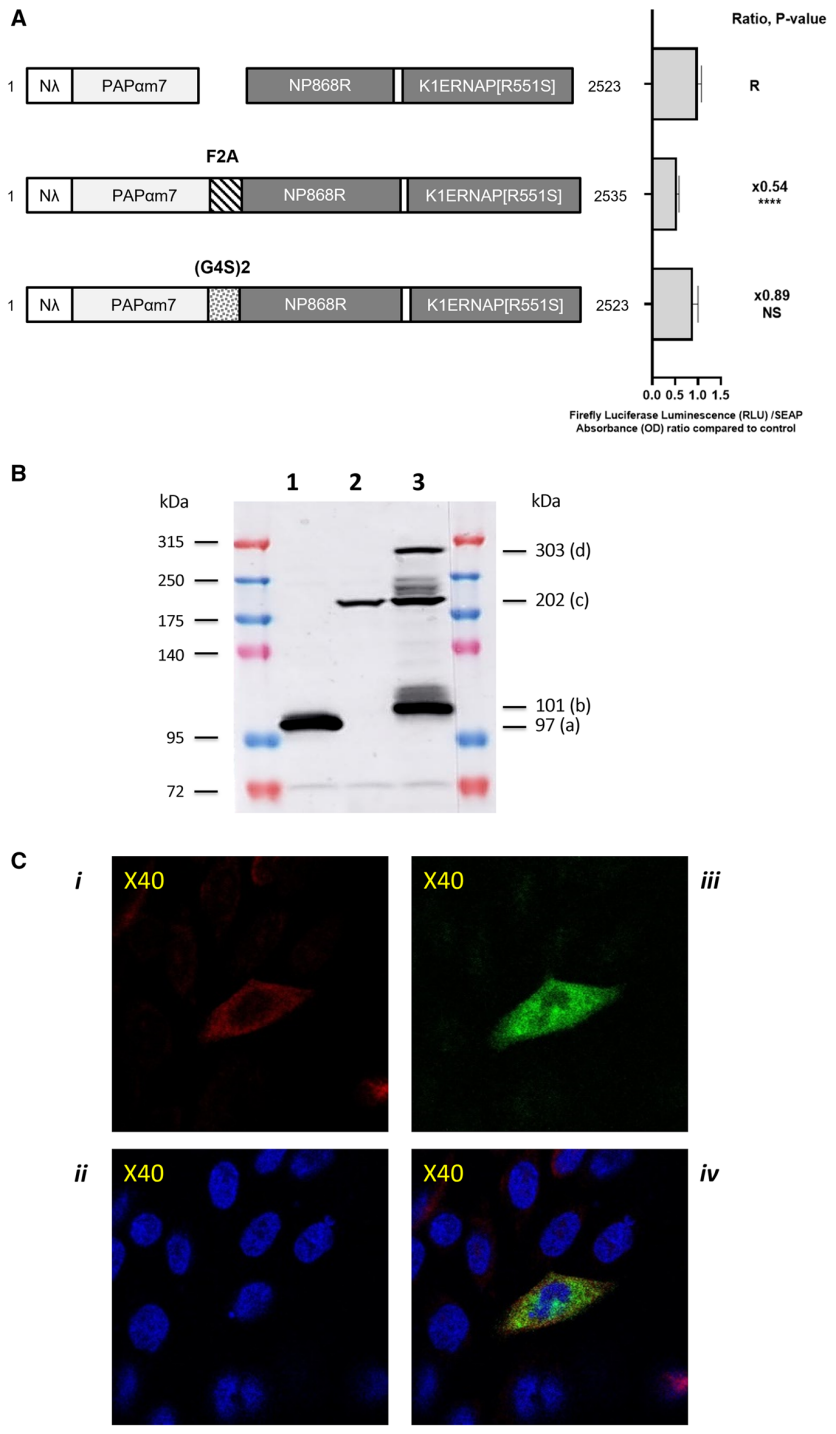
Design and optimization of single-ORF assembled C3P3-G2 enzymes. (**A**) Left: design of the Nλ-PAPαm7 and C3P3-G1 protein, as well the C3P3-G2 enzymes encoded by a single-ORF, which consist of the Nλ-PAPαm7 protein linked to the C3P3-G1 enzyme through either a pseudo-cleaving ribosome-skipping F2A sequence or a (G4S)2 flexible linker. Right: Firefly luciferase expression with these constructs transfected in HEK-293 cell was compared to the co-transfection of the Nλ-PAPαm7 and C3P3-G1 plasmids. Constructions were tested for luciferin oxidation assays 48 h after transfection and expressed as RLU divided by the hSEAP protein expression expressed in DO (RLU/DO ratio) and normalized to the reference group (R). Values represent the mean ± SD (n ≥ 4). Statistical analysis was performed by ANOVA adjusted by Dunnett’s Post Hoc analysis for multiple comparisons (*P < 0.05; **P < 0.01; ***P < 0.001; ****P < 0.0001). (**B**) Efficiency of the pseudo-cleavage by ribosome skipping of the C3P3-G2 protein assessed by Western blot. HEK-293 cells were transfected with the single tagged Nλ-PAPαm7-3xFLAG (lane 1) or the 3xFLAG-NP868R-(G4S)2-K1ERNAP(R551S) plasmids (lane 2), as well as the two-tagged single-ORF C3P3-G2 plasmid encoding for Nλ-PAPαm7-3xFLAG-F2A-3xFLAG-NP868R-(G4S)2-K1ERNAP(R551S) (lane 3). Protein lysates were subjected to western blotting with an anti-FLAG tag polyclonal antibody. The Nλ-PAPαm7-3xFLAG and 3xFLAG-NP868R-(G4S)2-K1ERNAP(R551S) protein were detected by Western-blotting as 97 kDa (band a) and 202 kDa bands (band c), respectively. Three major bands of 101, 202 and 303 kDa were observed with the single-ORF C3P3-G2 lysate, which correspond respectively to Nλ-PAPαm7-3xFLAG-F2A, (band b) 3xFLAG-NP868R-(G4S)2-K1ERNAP(R551S) (band c), and the read-through Nλ-PAPαm7-3xFLAG-F2A-3xFLAG-NP868R-(G4S)2-K1ERNAP(R551S) protein (band d). (**C**) Immunofluorescence of the single ORF C3P3-G2 protein. CHO-K1 cells were transfected with the C3P3-G2 plasmid that encodes for the two-tagged C3P3-G2 enzyme and imaged by indirect immunofluorescence. The Nλ-PAPαm7 protein was labeled with red fluorescent dye (*i*), while the NP868R-(G4S)2-K1ERNAP(R551S) protein was labelled in green (*ii*). Both proteins are essentially, if not exclusively, located in the cytoplasm. Nucleus were stained by Hoechst 33342 (*iii*). An orange fluorescence is also visible in the merged images is possibly related to double labelling of the read-through protein and the emission of green and red fluorescence in close vicinity (*iv*).

An important feature of the C3P3-G1 system is that the enzyme can be encoded by a single ORF, which makes it easier to use for certain applications, in particular therapeutics. We therefore attempted to generate a single ORF enzyme by in-frame fusion of the Nλ-PAPαm7 coding sequence to the amino-terminus of the NP868R-(G4S)2-K1ERNAP(R551S) C3P3-G1 enzyme. Conversely, reverse constructions by in-frame fusion to the carboxyl-terminus of C3P3-G1 were not tested because phage RNA polymerases do not tolerate carboxyl-terminal extension^57,58^. We tested two types of constructions, either by ligation though a flexible (G_4_S)_2_ linker therefore resulting in the production of a single-unit protein, or through a ribosome skipping F2A sequence from the Aphtovirus (Fig. 7A). The 2A sequences found in different virus species, among which the F2A sequence from the foot-and-mouth disease virus, prevents the ribosome from covalently generate a glycyl-prolyl peptide bond at the C-terminus of the F2A and thereby continue protein translation leading to an apparent co-translational cleavage and a two subunits enzyme^59^. These two constructions were tested under the same conditions as previously. Since higher expression of the Firefly luciferase reporter protein was found with the ribosome skipping F2A sequence than with the flexible linker (G4S)2, the former sequence was selected as the final assembled C3P3-G2 enzyme.

Previous findings have shown variable efficacy of pseudo-cleavage by F2A, either with a fraction of the F2A-containing proteins remaining as a read-through fusion, or with decreased expression of the downstream peptide due to ribosome fall-off^60^. We therefore evaluated the efficacy of F2A-mediated pseudo-cleavage of the C3P3-G2 enzyme into two subunits using Western blot analysis. Each subunit was separately tagged with an in-frame 3xFLAG tag either at the carboxyl-terminal end of Nλ-PAPαm7, or at the amino-terminal end of the NP868R-(G4S)2-K1ERNAP(R551S) protein. The tagged Nλ-PAPαm7-3xFLAG was detected as a 97 kDa band (Fig. 7B, track 1), and for the tagged 3xFLAG-NP868R-(G4S)2-K1ERNAP(R551S) subunit (track 2) as a 202 kDa band (Fig. 7B, track 2). We then immunoblotted the C3P3-G2 protein in which two 3xFLAG tags were inserted in-frame: one immediately before the F2A sequence downstream of Nλ-PAPαm7-3xFLAG, the other immediately after the F2A sequence upstream of 3xFLAG-NP868R-(G4S)2-K1ERNAP(R551S) protein. Three major bands of 101 kDa (Nλ-PAPαm7-3xFLAG), 202 kDa (3xFLAG-NP868R-(G4S)2-K1ERNAP(R551S)), and 303 kDa (Nλ-PAPαm7-3xFLAG-F2A-3xFLAG-NP868R-(G4S)2-K1ERNAP(R551S) read-through protein) were detected (Fig. 7B, track 3). These results confirm that although the ribosome-skipping F2A allows the production of the two subunits as expected, it also induces the production of a single fusion protein by read-through, as well as a greater proportion of the upstream subunit than the downstream one due to ribosome fall-off. Other technical solutions remain to be explored to avoid such read-through and fall-off products, the use of mutant ubiquitin to replace 2A peptides appearing particularly attractive^61^.

Finally, we imaged the C3P3-G2 protein by immunofluorescence by introducing two different labels into each of the subunits, which were both found in the cytoplasmic compartment (Fig. 7C).

## Discussion

We previously introduced the first generation of the C3P3 system, which to the best of our knowledge, is the first artificial non-viral system that enables in vivo autonomous production of mature mRNA^3^. Although this system is conceptually adaptable throughout the eukaryotic kingdom due to the conservation of the majority of mRNA post-transcriptional modifications, it has been mainly optimized by us for the human species and more generally mammals.

The C3P3-G1 system enables the production of mRNA having 5′-end ^m7^GpppN cap, a post-transcriptional modification which is critical for mRNA translation. It relies on an artificial single subunit chimeric enzyme, which is formed by the fusion of a mutant DNA-dependent RNA polymerase from Enterobacteria bacteriophage K1E and the capping enzyme of the African swine fever virus. The RNA polymerase moiety of the C3P3-G1 enzyme allows the transcription of genes under control of its promoter, whereas its capping enzyme moiety ensures efficient ^m7^GpppN capping of target mRNAs. Noticeably, a complete ^m7^GpppN cap can be synthesized by the capping enzyme, which has all three enzymatic activities necessary for its synthesis: 5′-triphosphatase that removes the γ-phosphate residue of 5′-triphosphate mRNA end resulting in diphosphate 5′-terminus, a guanylyltransferase that transfers GMP from GTP to diphosphate 5′-terminus, and a N7-guanine methyltransferase that adds a methyl residue onto nitrogen-7 of guanine resulting in ^m7^GpppN 5′-cap^3^.

Polyadenylation is another important post-translational mRNA modification, consisting of the addition of homopolymeric sequences at 3′-ends of mRNA, which is carried out by poly(A) polymerases. In mammalian cells, nuclear poly(A) polymerases are part of complexes of more than a dozen individual subunits that are physically coupled to RNA polymerase II via the carboxy-terminal domain of its largest subunit^62^. Upon recognition of polyadenylation signal sequences and enhancer elements on the pre-mRNA, this complex cleaves the pre-mRNAs and then mediates the synthesis of the poly(A) tail. Upon exiting the nucleus, the poly(A) tail is considered to have a fairly constant length of 250–300 adenosine in mammalian cells, but which actually appears to be quite variable depending on the species and cell type^13–17^. Once in the cytoplasm, the length of the poly(A) tails then becomes very heterogeneous by deadenylation at variable rates, which is a key mechanism for regulating the translatability and stability of the mRNA^9,12^.

Unlike nuclear polyadenylation, which is non-templated, that by the C3P3-G1 enzyme is templated, since it is produced by the transcription of a short track of 40 adenosines in the 3′UTR of the DNA template, followed by a trans-cleaving ribozyme from the hepatitis D virus. As the use of a longer adenosine track in the DNA templates did not provide an acceptable solution due to low production yields of the corresponding plasmids and the risk of plasmid recombination, we have opted for the development of a non-templated post-transcriptional polyadenylation system by an engineered poly(A) polymerase. To selectively enable polyadenylation of mRNA synthesized by the C3P3 system, we have developed an orthogonal RNA–protein interaction system that brings a mouse mutant PAPα fused to the N-peptide from the λ virus to the mRNA target in which 4xλBoxBr harpins were introduced the 3′UTR. As shown by the poly(A) tailing assay, this system makes it possible to extend the poly(A) tail to a variable length of up to about 250 nucleotides.

One of the mechanisms that can account for the increase in the level of protein expression by the Nλ-PAPαm7/4xλBoxBr system is the improvement of mRNA translatability. The poly(A) tail is indeed necessary for the formation of mRNA closed-loop which is a state of translation initiation resulting from the interaction of the poly(A) binding protein (PABP) bound to the 3′ poly(A) tail, eIF4E that binds to the 5′ capping and eIF4G that simultaneously binds eIF4E and PABP^63–65^. This pseudo-circularization is thought to promote the engagement of terminating ribosomes to a new round of translation at the same mRNA molecule, thus accounting for the functional synergy between ^m7^GpppN capping and 3′ poly(A) tail for enhancing protein translation. This has been well demonstrated by the pioneering study by Gallie et al., which has shown that the addition of poly(A_50_) tail increases by 156-fold the protein expression of a Firefly luciferase reporter mRNA electroporated in CHO cells compared to ^m7^GpppN capped mRNA without poly(A) tail, whereas polyadenylation has minimal effect in the absence of capping^66^. This is in agreement with the clear increase in translatability of mRNAs synthesized with C3P3 by the Nλ-PAPαm7/4xλBoxBr system. However, translatability remained lower than that of mRNA produced by a transgene with a conventional nuclear promoter, suggesting that other translation-limiting factors are involved.

Several hypotheses could explain the low translatability of the transcripts produced by C3P3, even with the Nλ-PAPαm7/4xλBoxBr system, which are not necessarily mutually exclusive. First of all, translation can be repressed by a type-I interferon response, which can be triggered by the absence of certain post-transcriptional modifications of the transcripts synthesized by the C3P3 system. These modifications could include adenosine-to-inosine editing^67^, internal modifications (e.g. N6-methyladenosine, 5-methylcytosine or pseudouridine)^68,69^, cap1 or cap2 2′-O-ribose-methylation at the first or second nucleotide of nascent mRNA^70^ or even reversible N6,2′-O-dimethyladenosine methylation at the first nucleotide^71^. Another molecular trigger of type-I interferon response could be the production of double-stranded RNA, which is an aberrant byproduct of transcription by phage RNA polymerases such as the one from the C3P3 enzyme^72^. Nevertheless, a massive interferon response would be difficult to reconcile with the quasi-normality of the polysomal profile observed with the C3P3-G1 system^3^, even if more subtle anomalies of translation initiation or elongation cannot be excluded. Second, the poor translatability of the newly synthesized RNA could be related to an inappropriate subcellular localization of the C3P3 enzyme. By analogy, the transcription and translation of nucleocytoplasmic large DNA viruses take place in virus-induced intracellular compartments called viral factories, where factors are recruited and concentrated, thus increasing the efficiency of the processes^73^. The absence of accumulation of these factors in the subcellular region of the C3P3 system could contribute to the poor translatability of the target mRNA. Third, the mRNA synthesis by the C3P3 system could exhaust the cell’s resources of nutrients, energy or oxygen, which could impair protein translation. For example, RNA synthesis by the C3P3 system probably consumes large amounts of nucleotides and could thus induce depletion of the nucleotide pool, by decreasing mRNA synthesis not only, but also with the production of non-coding rRNAs which are the main components of ribosomes^74^. Deciphering which mechanisms are involved is a crucial avenue to further improve the performance of the C3P3 system.

Polyadenylation also plays a central role in mRNA stability through the regulation of its decay^8^. Indeed, eukaryotic mRNA decay typically begins with poly(A) tail shortening by exonucleases, including the poly(A)-specific ribonuclease (PARN), Pan2/Pan3 complex and CCR4–NOT complex^75^. Therefore, the length of the poly(A) tail is generally positively correlated with the half-life of mRNA^75^. It is therefore likely that the same extension of mRNA half-life is conferred by the Nλ-PAPαm7/4xλBoxBr system, but unfortunately this hypothesis could not be tested in this present study due to the absence of a potent specific inhibitor of the phage RNA polymerase moiety of the enzyme C3P3.

The C3P3 system is a versatile platform technology well suited for many *in cellulo* applications, such as the rescue of wild-type or recombinant RNA viruses by reverse genetics. For example, the C3P3-G1 system was found to increase by 5–10-fold viral protein expression and by 50–100-fold reovirus titers in rescue experiments compared to the standard expression system^76^. Additionally, C3P3-G1 has been shown to rescue > 80% of rotavirus strains by reverse genetics in MA104 N*V cells, in contrast to standard strategies that rescue only < 20% of strains^77,78^. The C3P3 system can also be adapted for the production of recombinant lentivirus vectors, which remains problematic with conventional techniques due to their low yields and biosafety. Preliminary results show that the C3P3 system increases lentiviral vector production yields by at least four times compared to conventional techniques, as well as their predicted biosafety^79^.

Furthermore, the C3P3 system is also of potential interest for the production of recombinant proteins in mammalian cells, for which quality and production yields are essential requirements. Although not yet tested on a large scale, the C3P3 system could meet these requirements for the following reasons. First, the C3P3 system was found to produce proteins of usual quality, which is consistent with the fact that this system has no specific effect on the cellular translation process. For example, human erythropoietin produced in human HEK-293 cells with the C3P3-G1 system was found to have normal functional activity, which requires correct protein folding and post-translational glycosylation^3^. Second, the current performance of the C3P3-G2 system is attractive for its use for bioproduction purposes in CHO and HEK-293, which are the main cell lines used for bioproduction^80^. Third, the C3P3 enzyme acts as an expression master, which makes it possible to simultaneously produce several proteins necessary for the formation of multi-subunit complexes^76,77^. Such multi-subunit proteins, and more particularly antibody-based therapies – monoclonal antibodies, bispecific antibodies and antibody–drug conjugates – have emerged as a major class of therapies accounting for 30% of all new drugs and 73% of biologics approved by the FDA in 2022^81^. Fourth, the C3P3-G1 enzyme is active in the cytoplasm or in the nuclear compartment^3^, therefore making the C3P3 system potentially usable for both transient and stable expression systems.

Still another attractive application of the C3P3 system is its use in vivo for human therapeutics. The therapeutic solution we are currently developing is based on the use of semi-synthetic DNA assembling the C3P3 gene, as well as one or more genes under the control of the C3P3 promoter. This non-viral system has the advantages of ease and speed of development, low production cost, high level of expression, and capacity to express simultaneously several proteins if needed. Actually, the delivery of such semi-synthetic DNA could certainly benefit from recent advances in the field of lipid nanoparticles for mRNA vaccination^2^. This system will initially be tested for non-viral genetic compensation for acute diseases involving slow-cycling/resting organs such as the liver or respiratory tract. Another interesting therapeutic application is prophylactic vaccination against infectious diseases, which could benefit from the ability of the C3P3 system to simultaneously express multiple antigens for greater vaccine efficacy and/or immunization against different strains of pathogens^82^. Similarly, encouraging results for curative solid tumor immunotherapy with synthetic mRNA vaccines also highlight the importance of simultaneous expression of multiple neoantigens^83^, making the C3P3 system attractive for this application.

In summary, we have successfully developed an artificial polyadenylation system that allows extension of the poly(A) tail of transcripts synthesized with the C3P3 system, thereby significantly increasing the performance of the system. This second generation constitutes an important step in the development of the C3P3 artificial expression system which encourages its continued improvement.

### Supplementary Information

Supplementary Information 1.

Supplementary Information 2.

Supplementary Video 1.

## Data Availability

The GenBank accession numbers (https://www.ncbi.nlm.nih.gov/genbank/) of the sequences of this article are OQ509033, OQ509034, OQ509035, OQ509036, OQ509037, OQ509038, OQ509039, OQ509040, OQ509041, OQ509042, OQ509043, OQ509044, OQ509045, OQ509046, OQ509047, OQ509048, OQ509049, OQ509050, OQ509051, OQ509052, OQ509053, OQ509054, OQ509055, OQ509056, OQ509057, OQ509058, OQ509059, OQ509060, OQ509061, and OQ509062.

## Abbreviations

C3P3-G1 and C3P3-G2: First and second generations of cytoplasmic capping-prone phage polymerase expression system
PAPα: Poly(A) polymerase alpha

## References

[1] Bulcha JT, Wang Y, Ma H, Tai PWL, Gao G (2021). Viral vector platforms within the gene therapy landscape. Signal Transduct. Target. Ther..

[2] Hou X, Zaks T, Langer R, Dong Y (2021). Lipid nanoparticles for mRNA delivery. Nat. Rev. Mater..

[3] Jais PH, Decroly E, Jacquet E, Le Boulch M, Jais A, Jean-Jean O, Eaton H, Ponien P, Verdier F, Canard B (2019). C3P3-G1: First generation of a eukaryotic artificial cytoplasmic expression system. Nucleic Acids Res..

[4] Kwapiszewska K, Szczepanski K, Kalwarczyk T, Michalska B, Patalas-Krawczyk P, Szymanski J, Andryszewski T, Iwan M, Duszynski J, Holyst R (2020). Nanoscale viscosity of cytoplasm is conserved in human cell lines. J. Phys. Chem. Lett..

[5] Yao J, Fan Y, Li Y, Huang L (2013). Strategies on the nuclear-targeted delivery of genes. J. Drug Target.

[6] Ielasi FS, Ternifi S, Fontaine E, Iuso D, Coute Y, Palencia A (2022). Human histone pre-mRNA assembles histone or canonical mRNA-processing complexes by overlapping 3'-end sequence elements. Nucleic Acids Res..

[7] Griesbach E, Schlackow M, Marzluff WF, Proudfoot NJ (2021). Dual RNA 3'-end processing of H2A.X messenger RNA maintains DNA damage repair throughout the cell cycle. Nat. Commun..

[8] Garneau NL, Wilusz J, Wilusz CJ (2007). The highways and byways of mRNA decay. Nat. Rev. Mol. Cell Biol..

[9] Parker R, Song H (2004). The enzymes and control of eukaryotic mRNA turnover. Nat. Struct. Mol. Biol..

[10] Beilharz TH, Preiss T (2007). Widespread use of poly(A) tail length control to accentuate expression of the yeast transcriptome. RNA.

[11] Gallie DR, Tanguay R (1994). Poly(A) binds to initiation factors and increases cap-dependent translation in vitro. J. Biol. Chem..

[12] Passmore LA, Coller J (2022). Roles of mRNA poly(A) tails in regulation of eukaryotic gene expression. Nat. Rev. Mol. Cell Biol..

[13] Legnini I, Alles J, Karaiskos N, Ayoub S, Rajewsky N (2019). FLAM-seq: Full-length mRNA sequencing reveals principles of poly(A) tail length control. Nat. Methods.

[14] Nicholson AL, Pasquinelli AE (2019). Tales of detailed poly(A) tails. Trends Cell. Biol..

[15] Subtelny AO, Eichhorn SW, Chen GR, Sive H, Bartel DP (2014). Poly(A)-tail profiling reveals an embryonic switch in translational control. Nature.

[16] Chang H, Lim J, Ha M, Kim VN (2014). TAIL-seq: Genome-wide determination of poly(A) tail length and 3' end modifications. Mol. Cell.

[17] Eisen TJ, Eichhorn SW, Subtelny AO, Lin KS, McGeary SE, Gupta S, Bartel DP (2020). The dynamics of cytoplasmic mRNA metabolism. Mol. Cell.

[18] Mitschka S, Mayr C (2022). Context-specific regulation and function of mRNA alternative polyadenylation. Nat. Rev. Mol. Cell Biol..

[19] Raab D, Graf M, Notka F, Schodl T, Wagner R (2010). The GeneOptimizer Algorithm: Using a sliding window approach to cope with the vast sequence space in multiparameter DNA sequence optimization. Syst. Synth. Biol..

[20] Jackson AL, Burchard J, Leake D, Reynolds A, Schelter J, Guo J, Johnson JM, Lim L, Karpilow J, Nichols K (2006). Position-specific chemical modification of siRNAs reduces "off-target" transcript silencing. RNA.

[21] Chan FK, Moriwaki K, De Rosa MJ (2013). Detection of necrosis by release of lactate dehydrogenase activity. Methods Mol. Biol..

[22] Jones LJ, Gray M, Yue ST, Haugland RP, Singer VL (2001). Sensitive determination of cell number using the CyQUANT cell proliferation assay. J. Immunol. Methods.

[23] Einhauer A, Jungbauer A (2001). The FLAG peptide, a versatile fusion tag for the purification of recombinant proteins. J. Biochem. Biophys. Methods.

[24] Schneider CA, Rasband WS, Eliceiri KW (2012). NIH image to ImageJ: 25 years of image analysis. Nat. Methods.

[25] Hanke T, Szawlowski P, Randall RE (1992). Construction of solid matrix-antibody-antigen complexes containing simian immunodeficiency virus p27 using tag-specific monoclonal antibody and tag-linked antigen. J. Gen. Virol..

[26] Lahm H, Doppler S, Dressen M, Werner A, Adamczyk K, Schrambke D, Brade T, Laugwitz KL, Deutsch MA, Schiemann M (2015). Live fluorescent RNA-based detection of pluripotency gene expression in embryonic and induced pluripotent stem cells of different species. Stem Cells.

[27] Kusov YY, Shatirishvili G, Dzagurov G, Gauss-Muller V (2001). A new G-tailing method for the determination of the poly(A) tail length applied to hepatitis A virus RNA. Nucleic Acids Res..

[28] Blom N, Sicheritz-Ponten T, Gupta R, Gammeltoft S, Brunak S (2004). Prediction of post-translational glycosylation and phosphorylation of proteins from the amino acid sequence. Proteomics.

[29] Chenna R, Sugawara H, Koike T, Lopez R, Gibson TJ, Higgins DG, Thompson JD (2003). Multiple sequence alignment with the Clustal series of programs. Nucleic Acids Res..

[30] Golab K, Krzystyniak A, Langa P, Pikula M, Kunovac S, Borek P, Trzonkowski P, Millis JM, Fung J, Witkowski P (2020). Effect of serum on SmartFlare RNA Probes uptake and detection in cultured human cells. Biomed. J. Sci. Tech. Res..

[31] Coller J, Wickens M (2007). Tethered function assays: An adaptable approach to study RNA regulatory proteins. Methods Enzymol..

[32] Kwak JE, Wang L, Ballantyne S, Kimble J, Wickens M (2004). Mammalian GLD-2 homologs are poly(A) polymerases. Proc. Natl. Acad. Sci. USA.

[33] Dickson KS, Thompson SR, Gray NK, Wickens M (2001). Poly(A) polymerase and the regulation of cytoplasmic polyadenylation. J. Biol. Chem..

[34] Greenblatt J, Nodwell JR, Mason SW (1993). Transcriptional antitermination. Nature.

[35] Correll CC, Swinger K (2003). Common and distinctive features of GNRA tetraloops based on a GUAA tetraloop structure at 1.4 A resolution. RNA.

[36] Priet S, Lartigue A, Debart F, Claverie JM, Abergel C (2015). mRNA maturation in giant viruses: Variation on a theme. Nucleic Acids Res..

[37] Iyer LM, Balaji S, Koonin EV, Aravind L (2006). Evolutionary genomics of nucleo-cytoplasmic large DNA viruses. Virus Res..

[38] Moure CM, Bowman BR, Gershon PD, Quiocho FA (2006). Crystal structures of the vaccinia virus polyadenylate polymerase heterodimer: Insights into ATP selectivity and processivity. Mol. Cell.

[39] Cao GJ, Sarkar N (1992). Identification of the gene for an Escherichia coli poly(A) polymerase. Proc. Natl. Acad. Sci. USA.

[40] Kashiwabara S, Noguchi J, Zhuang T, Ohmura K, Honda A, Sugiura S, Miyamoto K, Takahashi S, Inoue K, Ogura A (2002). Regulation of spermatogenesis by testis-specific, cytoplasmic poly(A) polymerase TPAP. Science.

[41] Raabe T, Murthy KG, Manley JL (1994). Poly(A) polymerase contains multiple functional domains. Mol. Cell Biol..

[42] Colgan DF, Murthy KG, Prives C, Manley JL (1996). Cell-cycle related regulation of poly(A) polymerase by phosphorylation. Nature.

[43] Vethantham V, Rao N, Manley JL (2008). Sumoylation regulates multiple aspects of mammalian poly(A) polymerase function. Genes Dev..

[44] Fischer U, Huber J, Boelens WC, Mattaj IW, Luhrmann R (1995). The HIV-1 Rev activation domain is a nuclear export signal that accesses an export pathway used by specific cellular RNAs. Cell.

[45] Colgan DF, Murthy KG, Zhao W, Prives C, Manley JL (1998). Inhibition of poly(A) polymerase requires p34cdc2/cyclin B phosphorylation of multiple consensus and non-consensus sites. EMBO J..

[46] Colgan DF, Manley JL (1997). Mechanism and regulation of mRNA polyadenylation. Genes Dev..

[47] Martin G, Jeno P, Keller W (1999). Mapping of ATP binding regions in poly(A) polymerases by photoaffinity labeling and by mutational analysis identifies a domain conserved in many nucleotidyltransferases. Protein Sci..

[48] Austin RJ, Xia T, Ren J, Takahashi TT, Roberts RW (2002). Designed arginine-rich RNA-binding peptides with picomolar affinity. J. Am. Chem. Soc..

[49] Ohto U, Shibata T, Tanji H, Ishida H, Krayukhina E, Uchiyama S, Miyake K, Shimizu T (2015). Structural basis of CpG and inhibitory DNA recognition by Toll-like receptor 9. Nature.

[50] Chen Q, Sun L, Chen ZJ (2016). Regulation and function of the cGAS-STING pathway of cytosolic DNA sensing. Nat. Immunol..

[51] Marzluff WF, Koreski KP (2017). Birth and death of histone mRNAs. Trends Genet..

[52] Roth N, Schon J, Hoffmann D, Thran M, Thess A, Mueller SO, Petsch B, Rauch S (2022). Optimised non-coding regions of mRNA SARS-CoV-2 vaccine CV2CoV improves homologous and heterologous neutralising antibody responses. Vaccines (Basel).

[53] Blakqori G, van Knippenberg I, Elliott RM (2009). Bunyamwera orthobunyavirus S-segment untranslated regions mediate poly(A) tail-independent translation. J. Virol..

[54] Vera-Otarola J, Soto-Rifo R, Ricci EP, Ohlmann T, Darlix JL, Lopez-Lastra M (2010). The 3' untranslated region of the Andes hantavirus small mRNA functionally replaces the poly(A) tail and stimulates cap-dependent translation initiation from the viral mRNA. J. Virol..

[55] Polacek C, Friebe P, Harris E (2009). Poly(A)-binding protein binds to the non-polyadenylated 3' untranslated region of dengue virus and modulates translation efficiency. J. Gen. Virol..

[56] Li CY, Liang Z, Hu Y, Zhang H, Setiasabda KD, Li J, Ma S, Xia X, Kuang Y (2022). Cytidine-containing tails robustly enhance and prolong protein production of synthetic mRNA in cell and in vivo. Mol. Ther. Nucleic Acids.

[57] Mookhtiar KA, Peluso PS, Muller DK, Dunn JJ, Coleman JE (1991). Processivity of T7 RNA polymerase requires the C-terminal Phe882-Ala883-COO- or "foot". Biochemistry.

[58] Gardner LP, Mookhtiar KA, Coleman JE (1997). Initiation, elongation, and processivity of carboxyl-terminal mutants of T7 RNA polymerase. Biochemistry.

[59] Donnelly MLL, Hughes LE, Luke G, Mendoza H, Ten Dam E, Gani D, Ryan MD (2001). The 'cleavage' activities of foot-and-mouth disease virus 2A site-directed mutants and naturally occurring '2A-like' sequences. J. Gen. Virol..

[60] Liu Z, Chen O, Wall JBJ, Zheng M, Zhou Y, Wang L, Vaseghi HR, Qian L, Liu J (2017). Systematic comparison of 2A peptides for cloning multi-genes in a polycistronic vector. Sci. Rep..

[61] Varshavsky A (2005). Ubiquitin fusion technique and related methods. Methods Enzymol..

[62] Hsin JP, Manley JL (2012). The RNA polymerase II CTD coordinates transcription and RNA processing. Genes Dev..

[63] Shirokikh NE, Preiss T (2018). Translation initiation by cap-dependent ribosome recruitment: Recent insights and open questions. Wiley Interdiscip. Rev. RNA.

[64] Archer SK, Shirokikh NE, Hallwirth CV, Beilharz TH, Preiss T (2015). Probing the closed-loop model of mRNA translation in living cells. RNA Biol..

[65] Alekhina OM, Terenin IM, Dmitriev SE, Vassilenko KS (2020). Functional cyclization of eukaryotic mRNAs. Int. J. Mol. Sci..

[66] Gallie DR (1991). The cap and poly(A) tail function synergistically to regulate mRNA translational efficiency. Genes Dev..

[67] Nishikura K (2016). A-to-I editing of coding and non-coding RNAs by ADARs. Nat. Rev. Mol. Cell Biol..

[68] Meyer KD, Saletore Y, Zumbo P, Elemento O, Mason CE, Jaffrey SR (2012). Comprehensive analysis of mRNA methylation reveals enrichment in 3' UTRs and near stop codons. Cell.

[69] Anderson BR, Muramatsu H, Nallagatla SR, Bevilacqua PC, Sansing LH, Weissman D, Kariko K (2010). Incorporation of pseudouridine into mRNA enhances translation by diminishing PKR activation. Nucleic Acids Res..

[70] Daffis S, Szretter KJ, Schriewer J, Li J, Youn S, Errett J, Lin TY, Schneller S, Zust R, Dong H (2010). 2'-O methylation of the viral mRNA cap evades host restriction by IFIT family members. Nature.

[71] Mauer J, Luo X, Blanjoie A, Jiao X, Grozhik AV, Patil DP, Linder B, Pickering BF, Vasseur JJ, Chen Q (2017). Reversible methylation of m(6)A(m) in the 5' cap controls mRNA stability. Nature.

[72] Dousis A, Ravichandran K, Hobert EM, Moore MJ, Rabideau AE (2023). An engineered T7 RNA polymerase that produces mRNA free of immunostimulatory byproducts. Nat. Biotechnol..

[73] Schmid M, Speiseder T, Dobner T, Gonzalez RA (2014). DNA virus replication compartments. J. Virol..

[74] Pelletier J, Riano-Canalias F, Almacellas E, Mauvezin C, Samino S, Feu S, Menoyo S, Domostegui A, Garcia-Cajide M, Salazar R (2020). Nucleotide depletion reveals the impaired ribosome biogenesis checkpoint as a barrier against DNA damage. EMBO J..

[75] Weill L, Belloc E, Bava FA, Mendez R (2012). Translational control by changes in poly(A) tail length: Recycling mRNAs. Nat. Struct. Mol. Biol..

[76] Eaton HE, Kobayashi T, Dermody TS, Johnston RN, Jais PH, Shmulevitz M (2017). African swine fever virus NP868R capping enzyme promotes reovirus rescue during reverse genetics by promoting reovirus protein expression, virion assembly, and RNA incorporation into infectious virions. J. Virol..

[77] Sanchez-Tacuba L, Feng N, Meade NJ, Mellits KH, Jais PH, Yasukawa LL, Resch TK, Jiang B, Lopez S, Ding S (2020). An optimized reverse genetics system suitable for efficient recovery of simian, human, and murine-like rotaviruses. J. Virol..

[78] Kawagishi T, Sanchez-Tacuba L, Feng N, Costantini VP, Tan M, Jiang X, Green KY, Vinje J, Ding S, Greenberg HB (2023). Mucosal and systemic neutralizing antibodies to norovirus induced in infant mice orally inoculated with recombinant rotaviruses. Proc. Natl. Acad. Sci. USA.

[79] Jais, P. H. & LeBoulch, M. In *International Society for Cell and Gene Therapy Annual Meeting *(Cytotherapy, ed.), Vol. 25, S186 (Cytotherapy, 2023).

[80] Dumont J, Euwart D, Mei B, Estes S, Kshirsagar R (2016). Human cell lines for biopharmaceutical manufacturing: History, status, and future perspectives. Crit. Rev. Biotechnol..

[81] Mullard A (2023). 2022 FDA approvals. Nat. Rev. Drug Discov..

[82] Freyn AW, Ramos da Silva J, Rosado VC, Bliss CM, Pine M, Mui BL, Tam YK, Madden TD, de Souza Ferreira LC, Weissman D (2020). A multi-targeting, nucleoside-modified mRNA influenza virus vaccine provides broad protection in mice. Mol. Ther..

[83] Rojas LA, Sethna Z, Soares KC, Olcese C, Pang N, Patterson E, Lihm J, Ceglia N, Guasp P, Chu A (2023). Personalized RNA neoantigen vaccines stimulate T cells in pancreatic cancer. Nature.

