# Supplementary material for: Rational design of an artificial tethered enzyme for non-templated post-transcriptional mRNA polyadenylation by the second generation of the C3P3 system
Source: Sci Rep. 2024 Mar 2;14:5156. doi: 10.1038/s41598-024-55947-0 (PMC10908868; doi:10.1038/s41598-024-55947-0)
Supplement: Supplementary file 1 — Supplementary Information 1. [file 41598_2024_55947_MOESM1_ESM.pptx]

## Slide 1
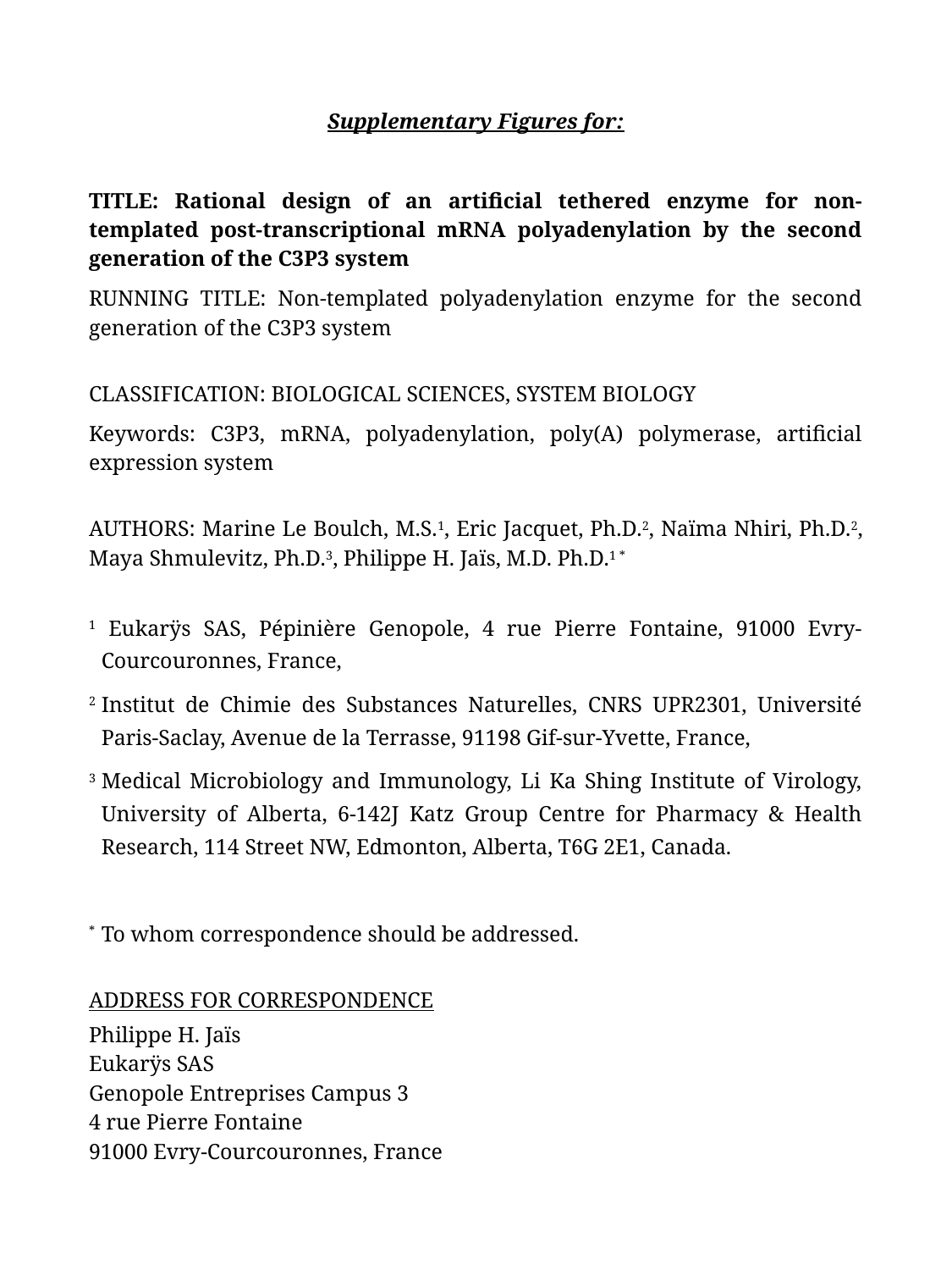

Supplementary Figures for:
TITLE: Rational design of an artificial tethered enzyme for non-templated post-transcriptional mRNA polyadenylation by the second generation of the C3P3 system
RUNNING TITLE: Non-templated polyadenylation enzyme for the second generation of the C3P3 system
Classification: biological sciences, System Biology
Keywords: C3P3, mRNA, polyadenylation, poly(A) polymerase, artificial expression system
Authors: Marine Le Boulch, M.S.1, Eric Jacquet, Ph.D.2, Naïma Nhiri, Ph.D.2, Maya Shmulevitz, Ph.D.3, Philippe H. Jaïs, M.D. Ph.D.1 *
1 	Eukarÿs SAS, Pépinière Genopole, 4 rue Pierre Fontaine, 91000 Evry-Courcouronnes, France,
2	Institut de Chimie des Substances Naturelles, CNRS UPR2301, Université Paris-Saclay, Avenue de la Terrasse, 91198 Gif-sur-Yvette, France,
3	Medical Microbiology and Immunology, Li Ka Shing Institute of Virology, University of Alberta, 6-142J Katz Group Centre for Pharmacy & Health Research, 114 Street NW, Edmonton, Alberta, T6G 2E1, Canada.
*	To whom correspondence should be addressed.
Address for correspondence
Philippe H. Jaïs
Eukarÿs SAS
Genopole Entreprises Campus 3
4 rue Pierre Fontaine
91000 Evry-Courcouronnes, France

## Slide 2
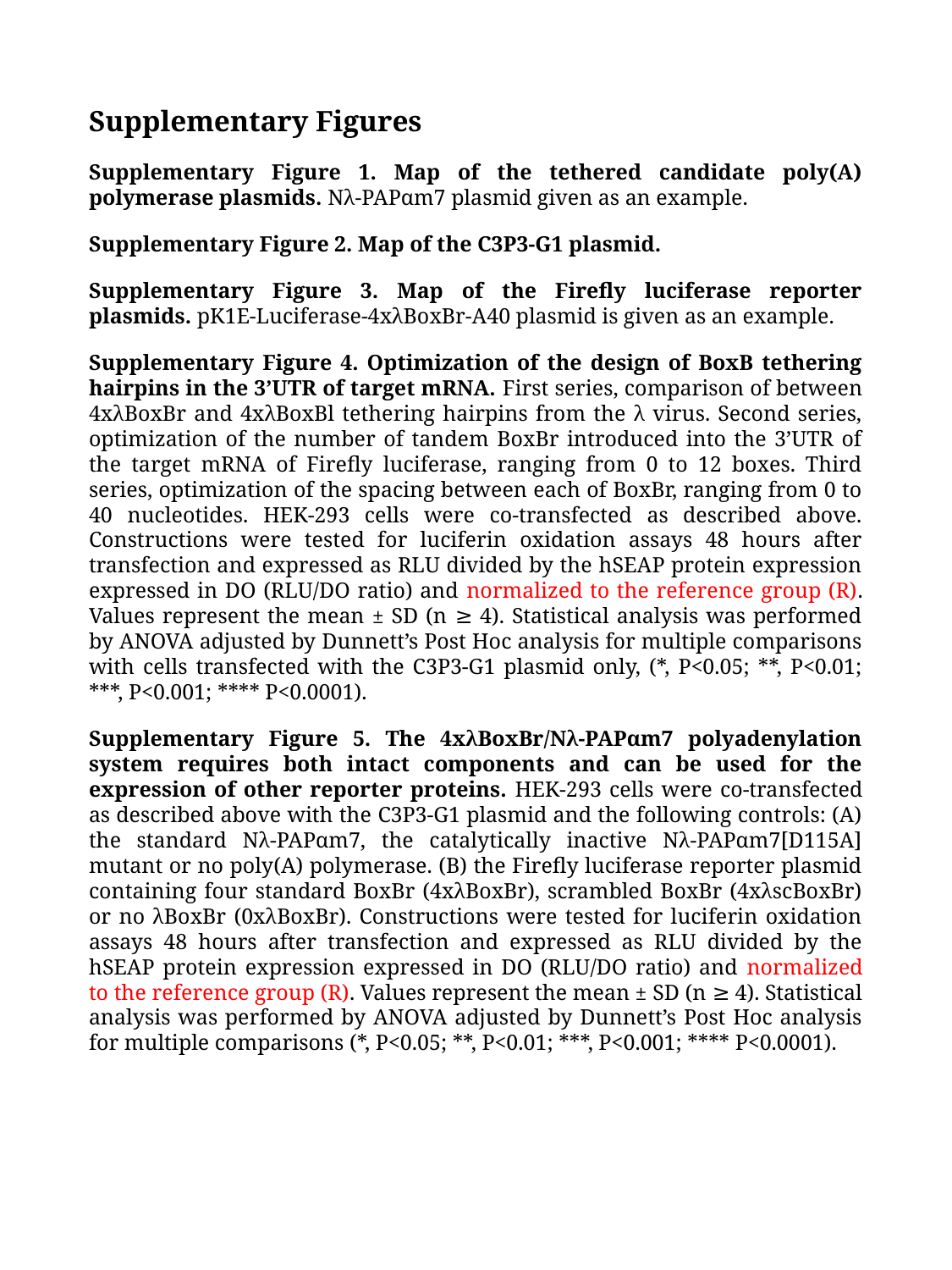

Supplementary Figures
Supplementary Figure 1. Map of the tethered candidate poly(A) polymerase plasmids. Nλ-PAPαm7 plasmid given as an example.
Supplementary Figure 2. Map of the C3P3-G1 plasmid.
Supplementary Figure 3. Map of the Firefly luciferase reporter plasmids. pK1E-Luciferase-4xλBoxBr-A40 plasmid is given as an example.
Supplementary Figure 4. Optimization of the design of BoxB tethering hairpins in the 3’UTR of target mRNA. First series, comparison of between 4xλBoxBr and 4xλBoxBl tethering hairpins from the λ virus. Second series, optimization of the number of tandem BoxBr introduced into the 3’UTR of the target mRNA of Firefly luciferase, ranging from 0 to 12 boxes. Third series, optimization of the spacing between each of BoxBr, ranging from 0 to 40 nucleotides. HEK-293 cells were co-transfected as described above. Constructions were tested for luciferin oxidation assays 48 hours after transfection and expressed as RLU divided by the hSEAP protein expression expressed in DO (RLU/DO ratio) and normalized to the reference group (R). Values represent the mean ± SD (n ≥ 4). Statistical analysis was performed by ANOVA adjusted by Dunnett’s Post Hoc analysis for multiple comparisons with cells transfected with the C3P3-G1 plasmid only, (*, P<0.05; **, P<0.01; ***, P<0.001; **** P<0.0001).
Supplementary Figure 5. The 4xλBoxBr/Nλ-PAPαm7 polyadenylation system requires both intact components and can be used for the expression of other reporter proteins. HEK-293 cells were co-transfected as described above with the C3P3-G1 plasmid and the following controls: (A) the standard Nλ-PAPαm7, the catalytically inactive Nλ-PAPαm7[D115A] mutant or no poly(A) polymerase. (B) the Firefly luciferase reporter plasmid containing four standard BoxBr (4xλBoxBr), scrambled BoxBr (4xλscBoxBr) or no λBoxBr (0xλBoxBr). Constructions were tested for luciferin oxidation assays 48 hours after transfection and expressed as RLU divided by the hSEAP protein expression expressed in DO (RLU/DO ratio) and normalized to the reference group (R). Values represent the mean ± SD (n ≥ 4). Statistical analysis was performed by ANOVA adjusted by Dunnett’s Post Hoc analysis for multiple comparisons (*, P<0.05; **, P<0.01; ***, P<0.001; **** P<0.0001).

## Slide 3
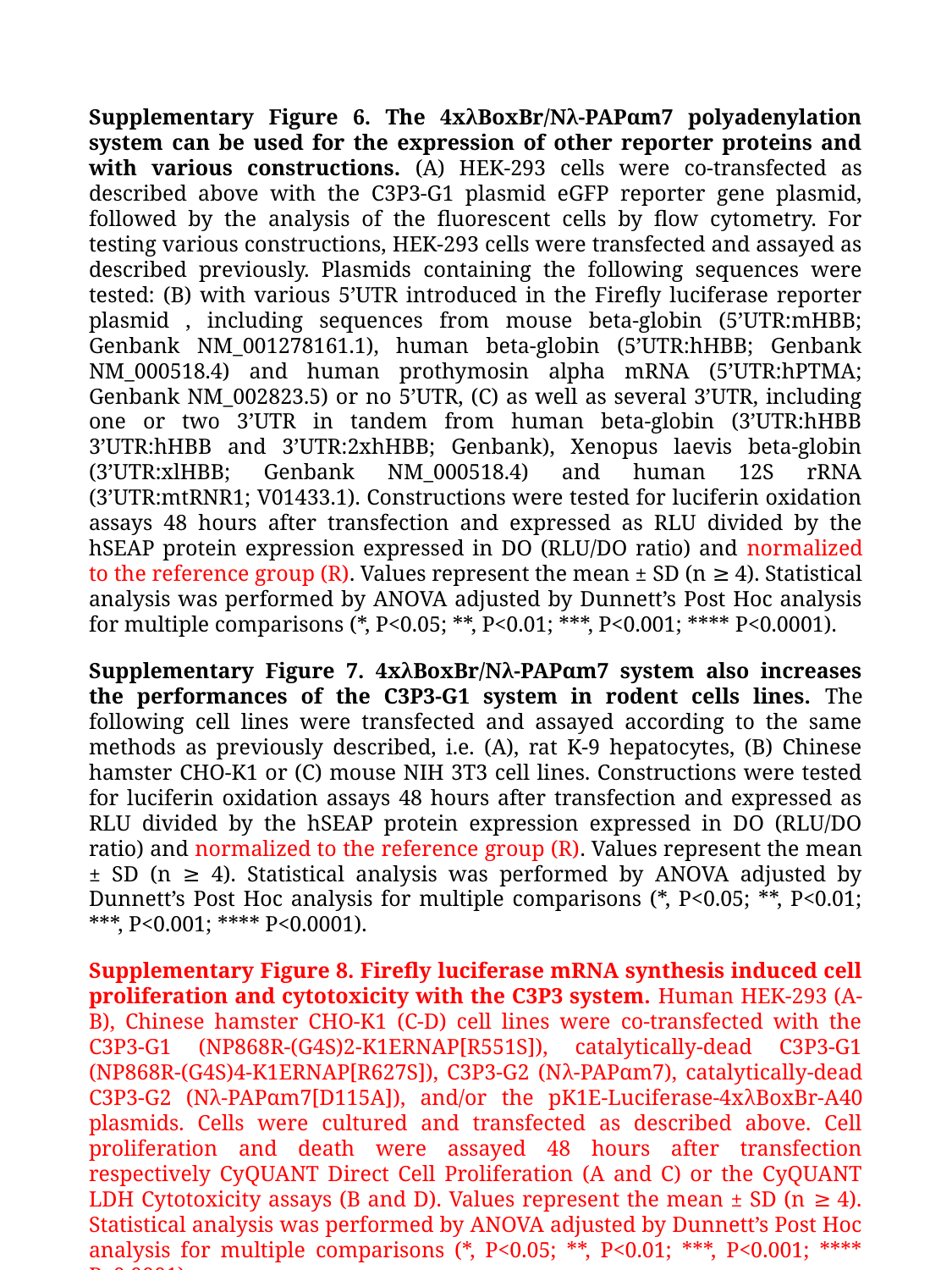

Supplementary Figure 6. The 4xλBoxBr/Nλ-PAPαm7 polyadenylation system can be used for the expression of other reporter proteins and with various constructions. (A) HEK-293 cells were co-transfected as described above with the C3P3-G1 plasmid eGFP reporter gene plasmid, followed by the analysis of the fluorescent cells by flow cytometry. For testing various constructions, HEK-293 cells were transfected and assayed as described previously. Plasmids containing the following sequences were tested: (B) with various 5’UTR introduced in the Firefly luciferase reporter plasmid , including sequences from mouse beta-globin (5’UTR:mHBB; Genbank NM_001278161.1), human beta-globin (5’UTR:hHBB; Genbank NM_000518.4) and human prothymosin alpha mRNA (5’UTR:hPTMA; Genbank NM_002823.5) or no 5’UTR, (C) as well as several 3’UTR, including one or two 3’UTR in tandem from human beta-globin (3’UTR:hHBB 3’UTR:hHBB and 3’UTR:2xhHBB; Genbank), Xenopus laevis beta-globin (3’UTR:xlHBB; Genbank NM_000518.4) and human 12S rRNA (3’UTR:mtRNR1; V01433.1). Constructions were tested for luciferin oxidation assays 48 hours after transfection and expressed as RLU divided by the hSEAP protein expression expressed in DO (RLU/DO ratio) and normalized to the reference group (R). Values represent the mean ± SD (n ≥ 4). Statistical analysis was performed by ANOVA adjusted by Dunnett’s Post Hoc analysis for multiple comparisons (*, P<0.05; **, P<0.01; ***, P<0.001; **** P<0.0001).
Supplementary Figure 7. 4xλBoxBr/Nλ-PAPαm7 system also increases the performances of the C3P3-G1 system in rodent cells lines. The following cell lines were transfected and assayed according to the same methods as previously described, i.e. (A), rat K-9 hepatocytes, (B) Chinese hamster CHO-K1 or (C) mouse NIH 3T3 cell lines. Constructions were tested for luciferin oxidation assays 48 hours after transfection and expressed as RLU divided by the hSEAP protein expression expressed in DO (RLU/DO ratio) and normalized to the reference group (R). Values represent the mean ± SD (n ≥ 4). Statistical analysis was performed by ANOVA adjusted by Dunnett’s Post Hoc analysis for multiple comparisons (*, P<0.05; **, P<0.01; ***, P<0.001; **** P<0.0001).
Supplementary Figure 8. Firefly luciferase mRNA synthesis induced cell proliferation and cytotoxicity with the C3P3 system. Human HEK-293 (A-B), Chinese hamster CHO-K1 (C-D) cell lines were co-transfected with the C3P3-G1 (NP868R-(G4S)2-K1ERNAP[R551S]), catalytically-dead C3P3-G1 (NP868R-(G4S)4-K1ERNAP[R627S]), C3P3-G2 (Nλ-PAPαm7), catalytically-dead C3P3-G2 (Nλ-PAPαm7[D115A]), and/or the pK1E-Luciferase-4xλBoxBr-A40 plasmids. Cells were cultured and transfected as described above. Cell proliferation and death were assayed 48 hours after transfection respectively CyQUANT Direct Cell Proliferation (A and C) or the CyQUANT LDH Cytotoxicity assays (B and D). Values represent the mean ± SD (n ≥ 4). Statistical analysis was performed by ANOVA adjusted by Dunnett’s Post Hoc analysis for multiple comparisons (*, P<0.05; **, P<0.01; ***, P<0.001; **** P<0.0001).

## Slide 4
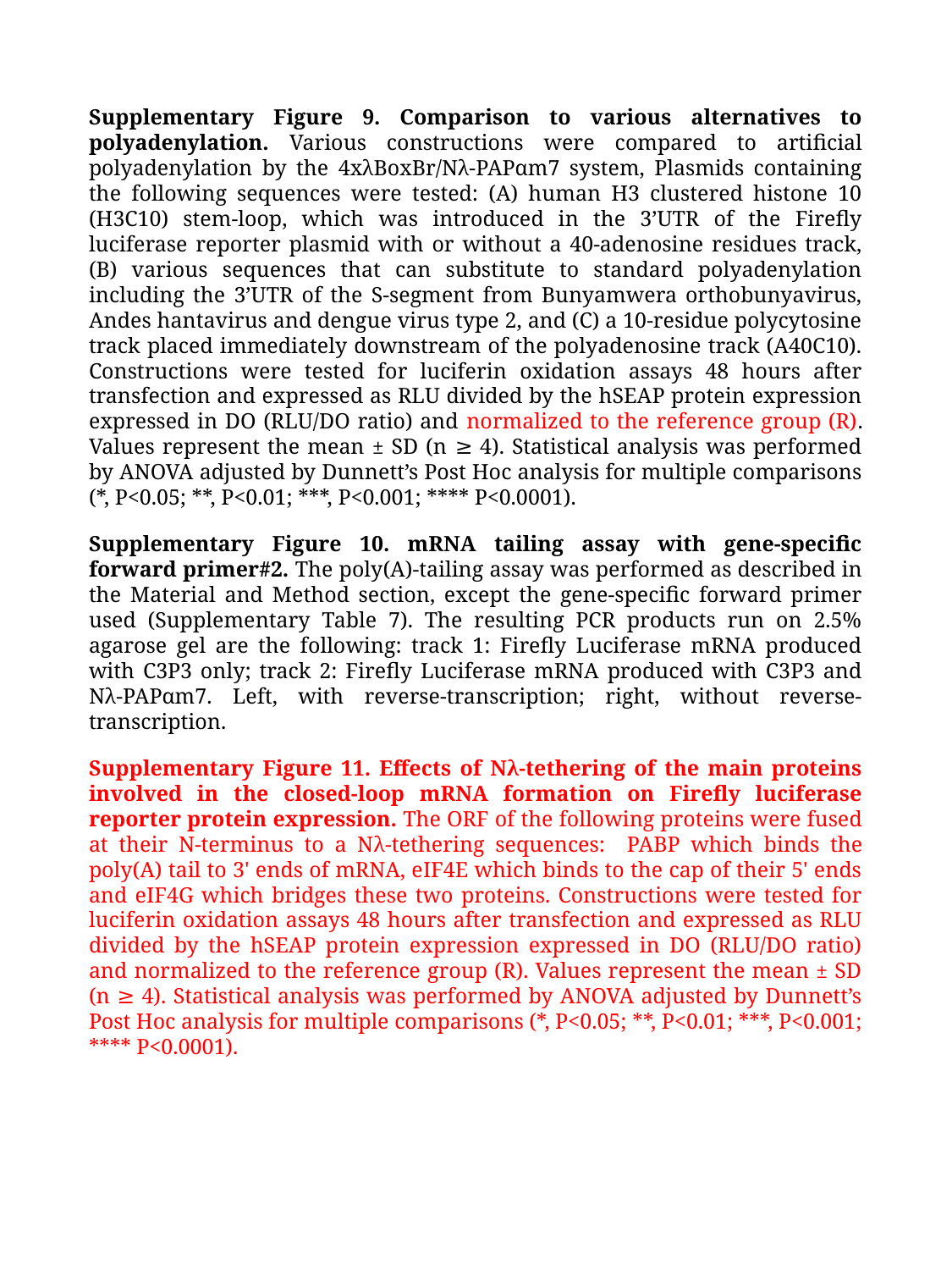

Supplementary Figure 9. Comparison to various alternatives to polyadenylation. Various constructions were compared to artificial polyadenylation by the 4xλBoxBr/Nλ-PAPαm7 system, Plasmids containing the following sequences were tested: (A) human H3 clustered histone 10 (H3C10) stem-loop, which was introduced in the 3’UTR of the Firefly luciferase reporter plasmid with or without a 40-adenosine residues track, (B) various sequences that can substitute to standard polyadenylation including the 3’UTR of the S-segment from Bunyamwera orthobunyavirus, Andes hantavirus and dengue virus type 2, and (C) a 10-residue polycytosine track placed immediately downstream of the polyadenosine track (A40C10). Constructions were tested for luciferin oxidation assays 48 hours after transfection and expressed as RLU divided by the hSEAP protein expression expressed in DO (RLU/DO ratio) and normalized to the reference group (R). Values represent the mean ± SD (n ≥ 4). Statistical analysis was performed by ANOVA adjusted by Dunnett’s Post Hoc analysis for multiple comparisons (*, P<0.05; **, P<0.01; ***, P<0.001; **** P<0.0001).
Supplementary Figure 10. mRNA tailing assay with gene-specific forward primer#2. The poly(A)-tailing assay was performed as described in the Material and Method section, except the gene-specific forward primer used (Supplementary Table 7). The resulting PCR products run on 2.5% agarose gel are the following: track 1: Firefly Luciferase mRNA produced with C3P3 only; track 2: Firefly Luciferase mRNA produced with C3P3 and Nλ-PAPαm7. Left, with reverse-transcription; right, without reverse-transcription.
Supplementary Figure 11. Effects of Nλ-tethering of the main proteins involved in the closed-loop mRNA formation on Firefly luciferase reporter protein expression. The ORF of the following proteins were fused at their N-terminus to a Nλ-tethering sequences: PABP which binds the poly(A) tail to 3' ends of mRNA, eIF4E which binds to the cap of their 5' ends and eIF4G which bridges these two proteins. Constructions were tested for luciferin oxidation assays 48 hours after transfection and expressed as RLU divided by the hSEAP protein expression expressed in DO (RLU/DO ratio) and normalized to the reference group (R). Values represent the mean ± SD (n ≥ 4). Statistical analysis was performed by ANOVA adjusted by Dunnett’s Post Hoc analysis for multiple comparisons (*, P<0.05; **, P<0.01; ***, P<0.001; **** P<0.0001).

## Slide 5
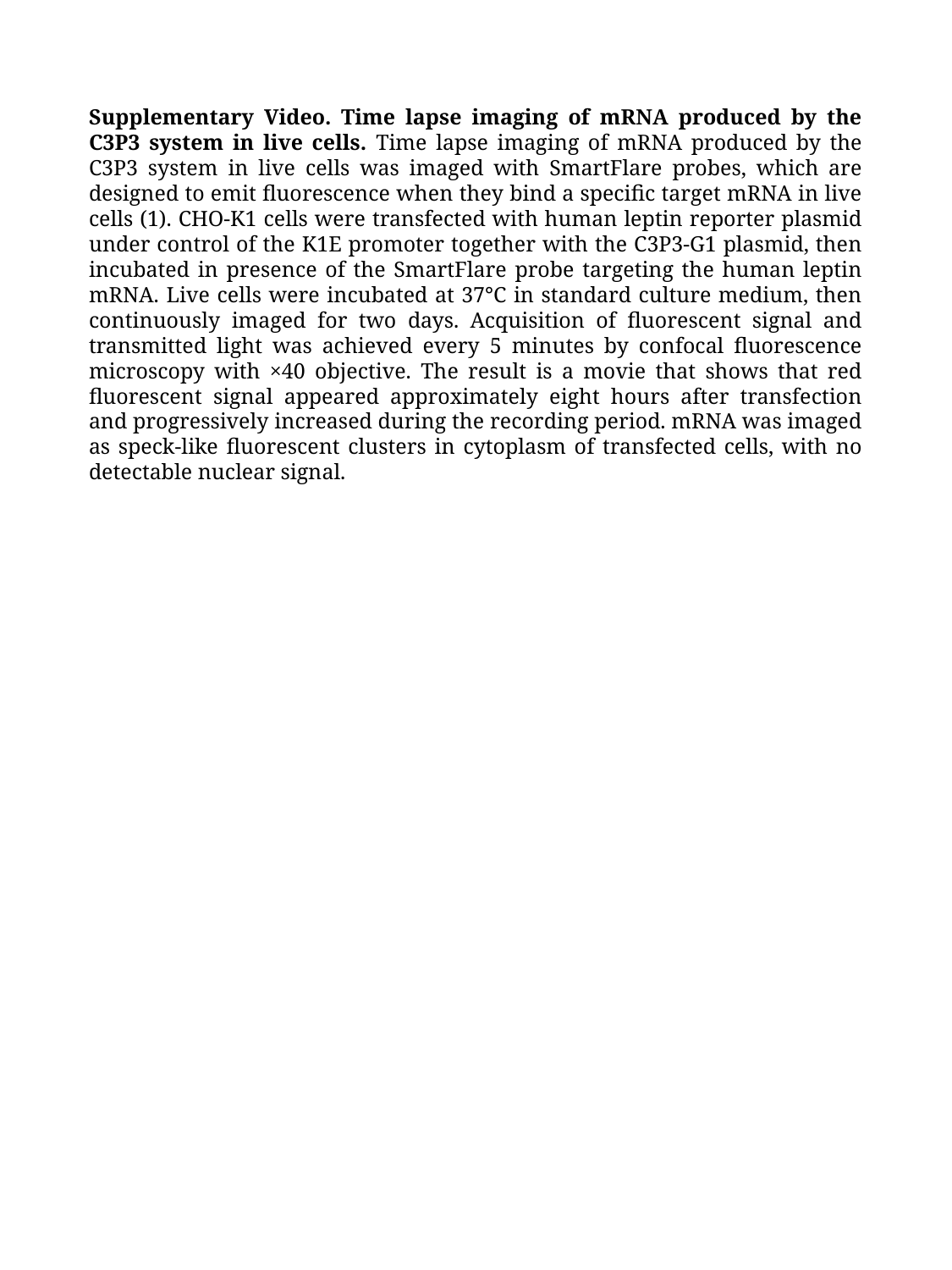

Supplementary Video. Time lapse imaging of mRNA produced by the C3P3 system in live cells. Time lapse imaging of mRNA produced by the C3P3 system in live cells was imaged with SmartFlare probes, which are designed to emit fluorescence when they bind a specific target mRNA in live cells (1). CHO-K1 cells were transfected with human leptin reporter plasmid under control of the K1E promoter together with the C3P3-G1 plasmid, then incubated in presence of the SmartFlare probe targeting the human leptin mRNA. Live cells were incubated at 37°C in standard culture medium, then continuously imaged for two days. Acquisition of fluorescent signal and transmitted light was achieved every 5 minutes by confocal fluorescence microscopy with ×40 objective. The result is a movie that shows that red fluorescent signal appeared approximately eight hours after transfection and progressively increased during the recording period. mRNA was imaged as speck-like fluorescent clusters in cytoplasm of transfected cells, with no detectable nuclear signal.

## Slide 6
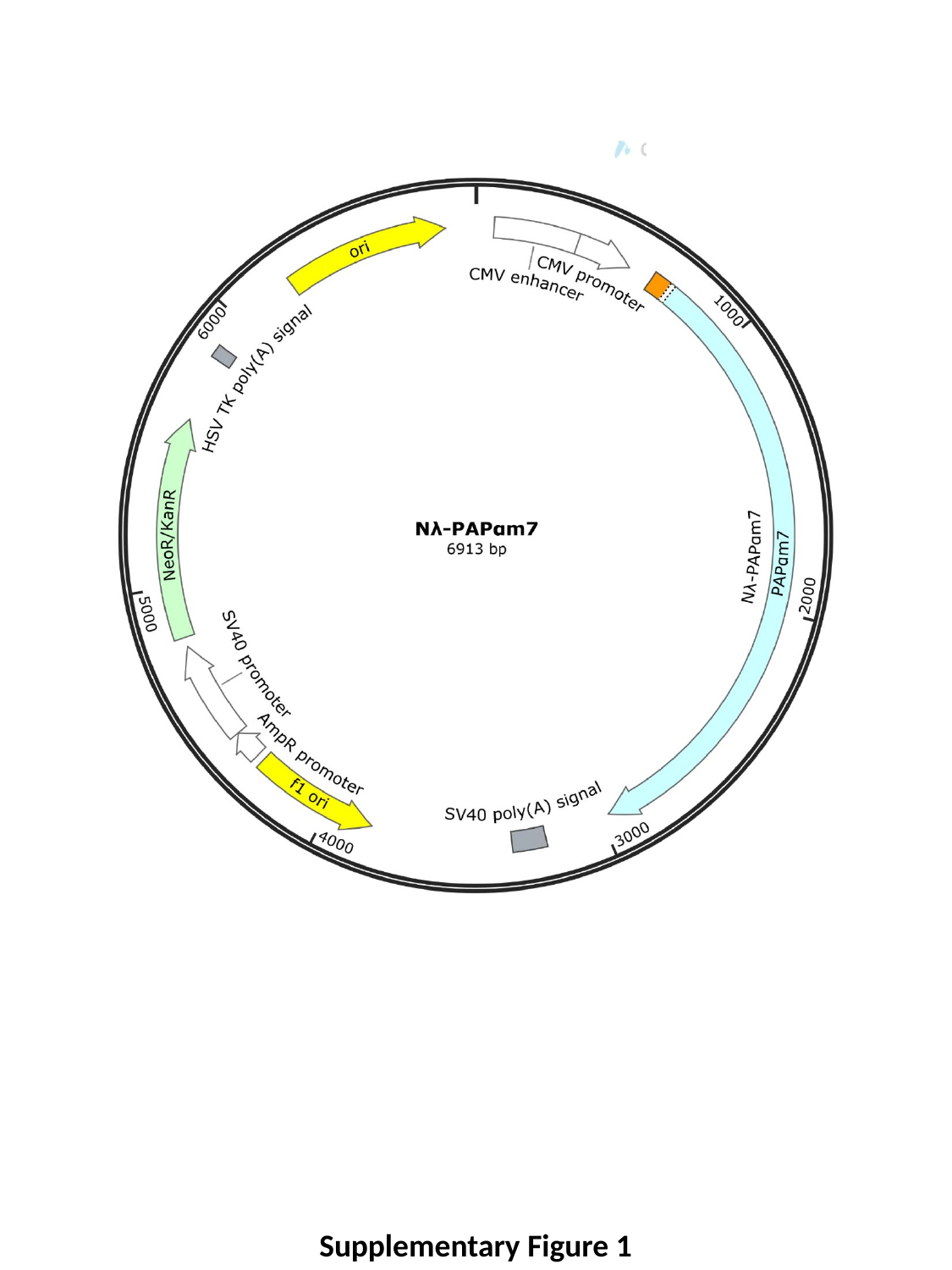

Supplementary Figure 1

## Slide 7
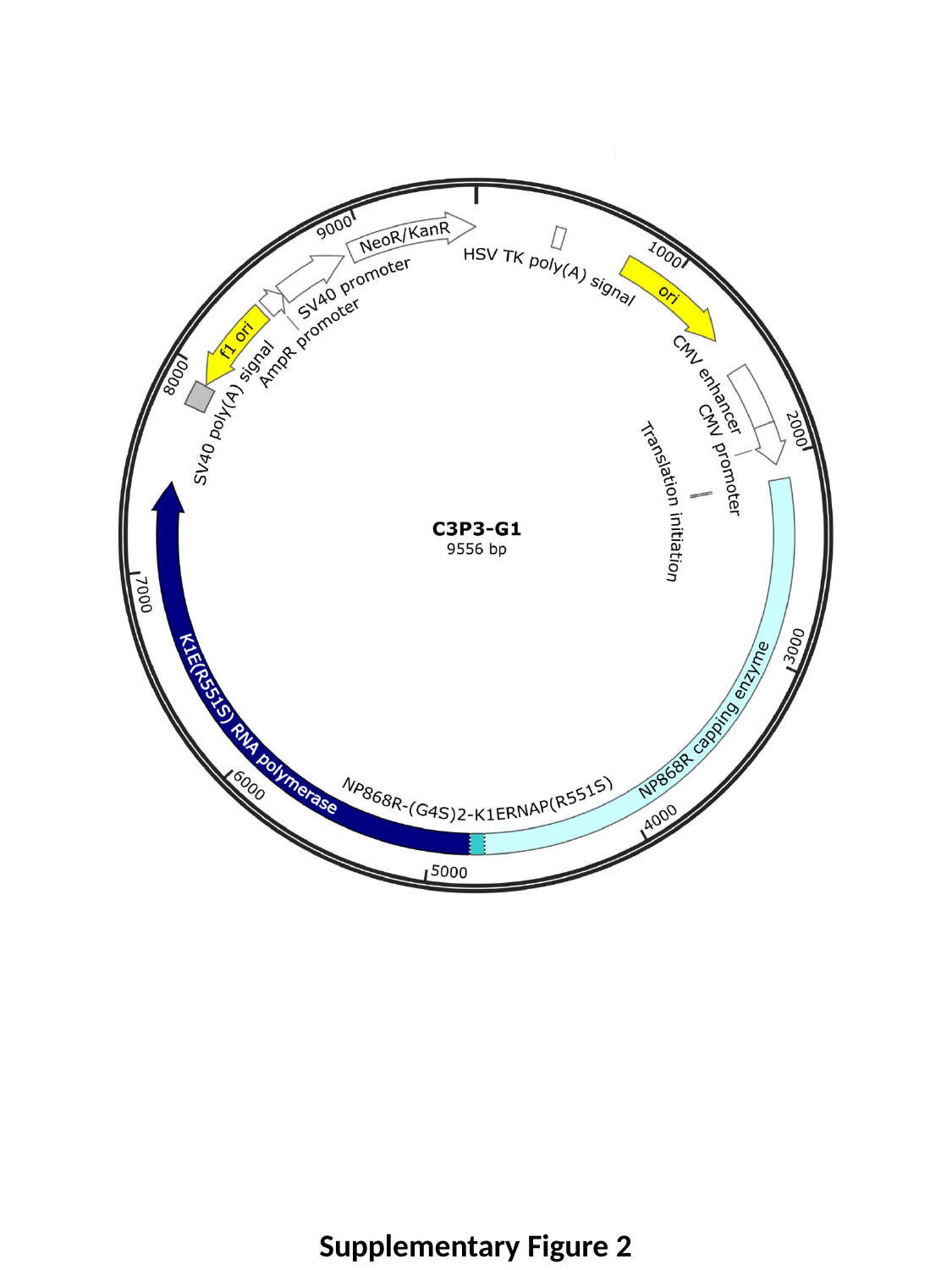

Supplementary Figure 2

## Slide 8
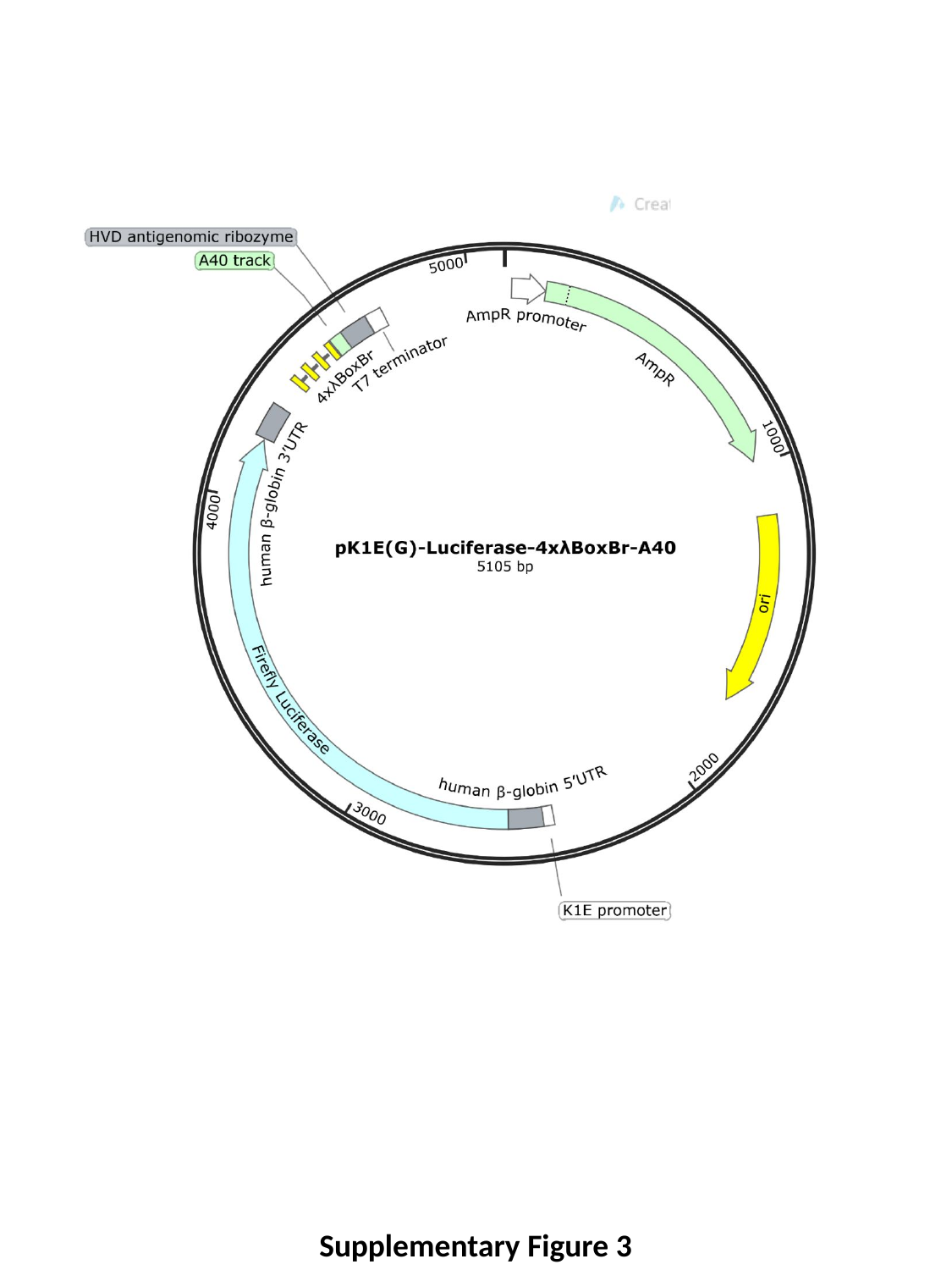

Supplementary Figure 3

## Slide 9
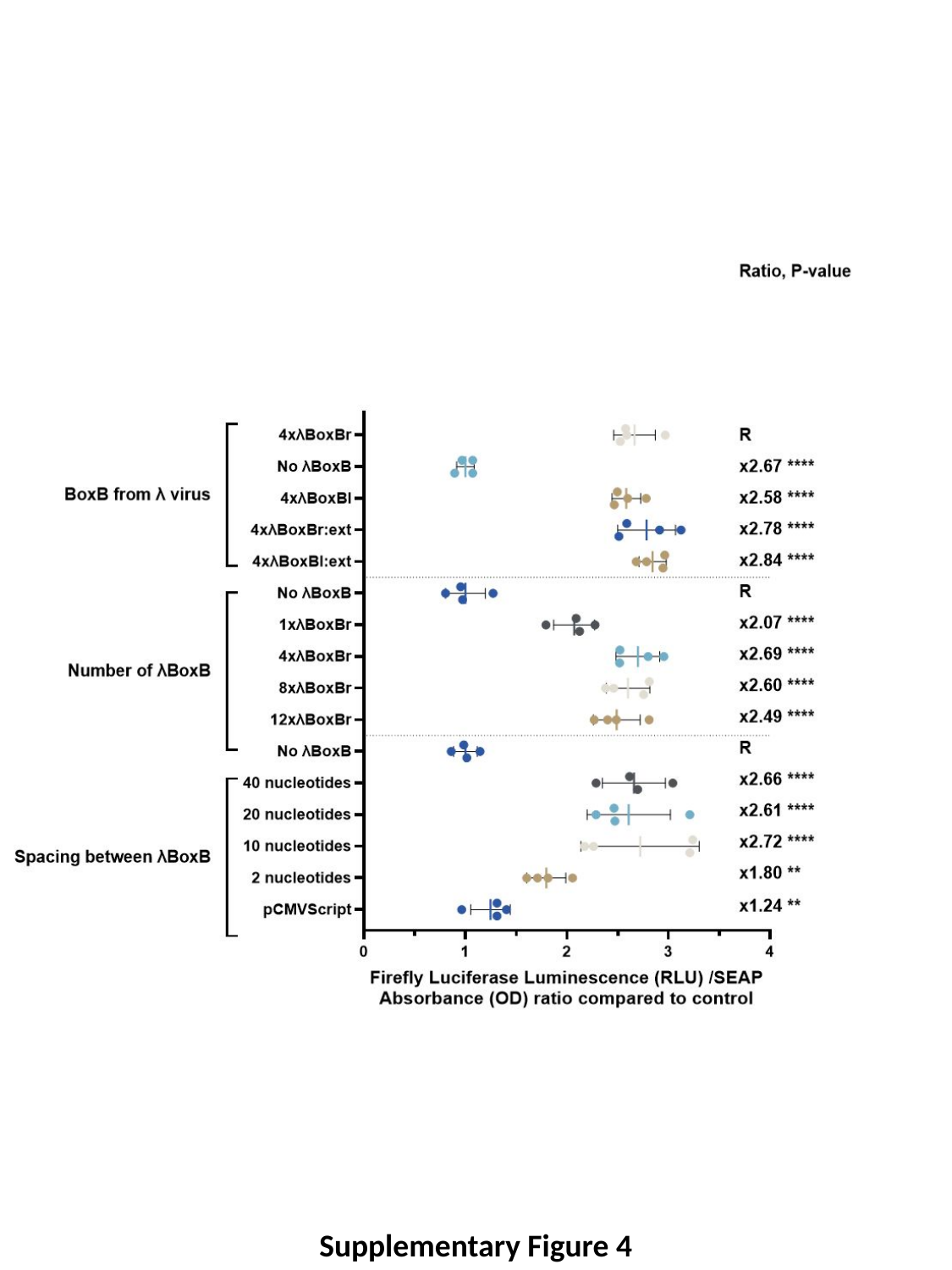

Supplementary Figure 4

## Slide 10
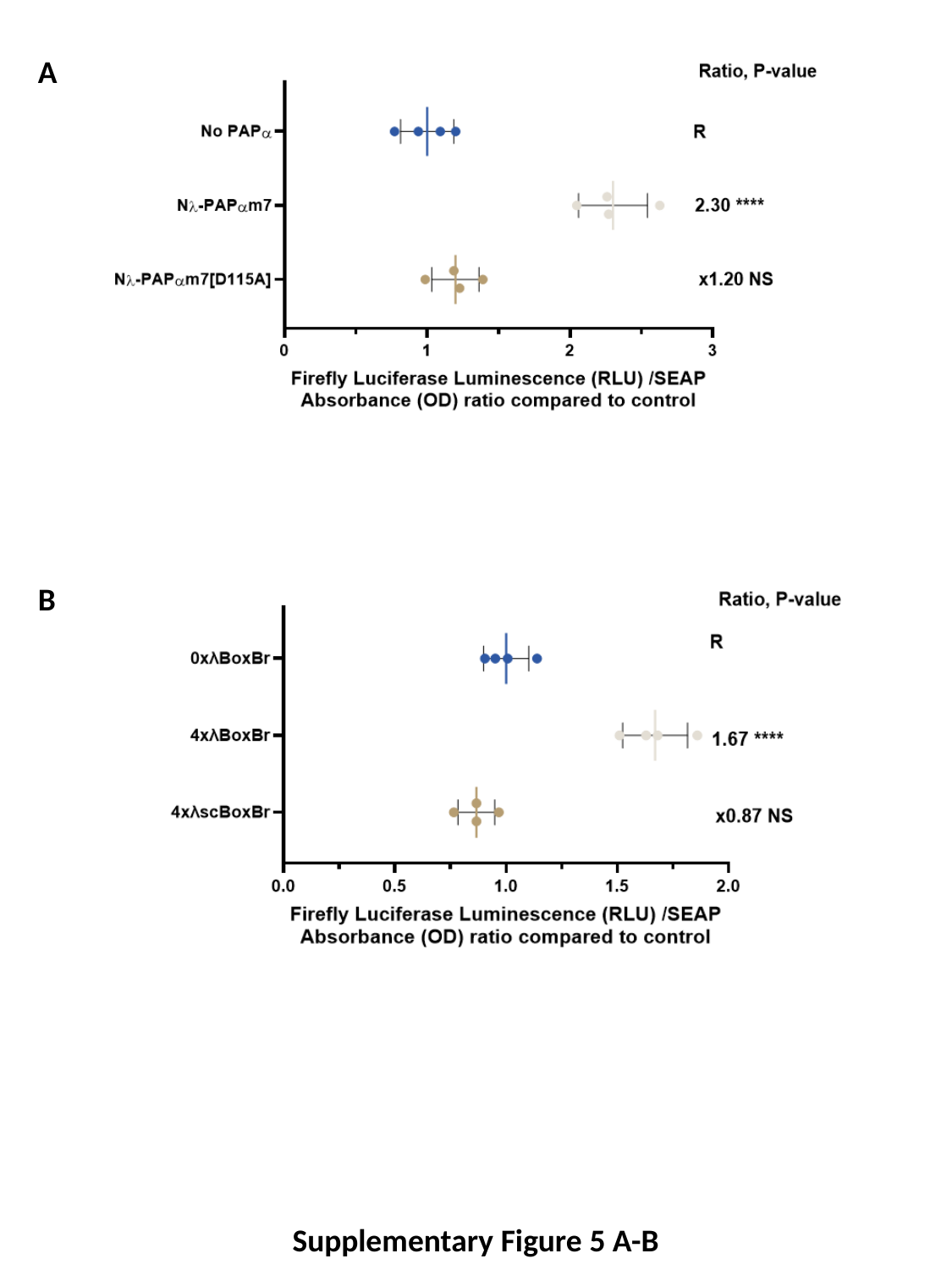

A
B
Supplementary Figure 5 A-B

## Slide 11
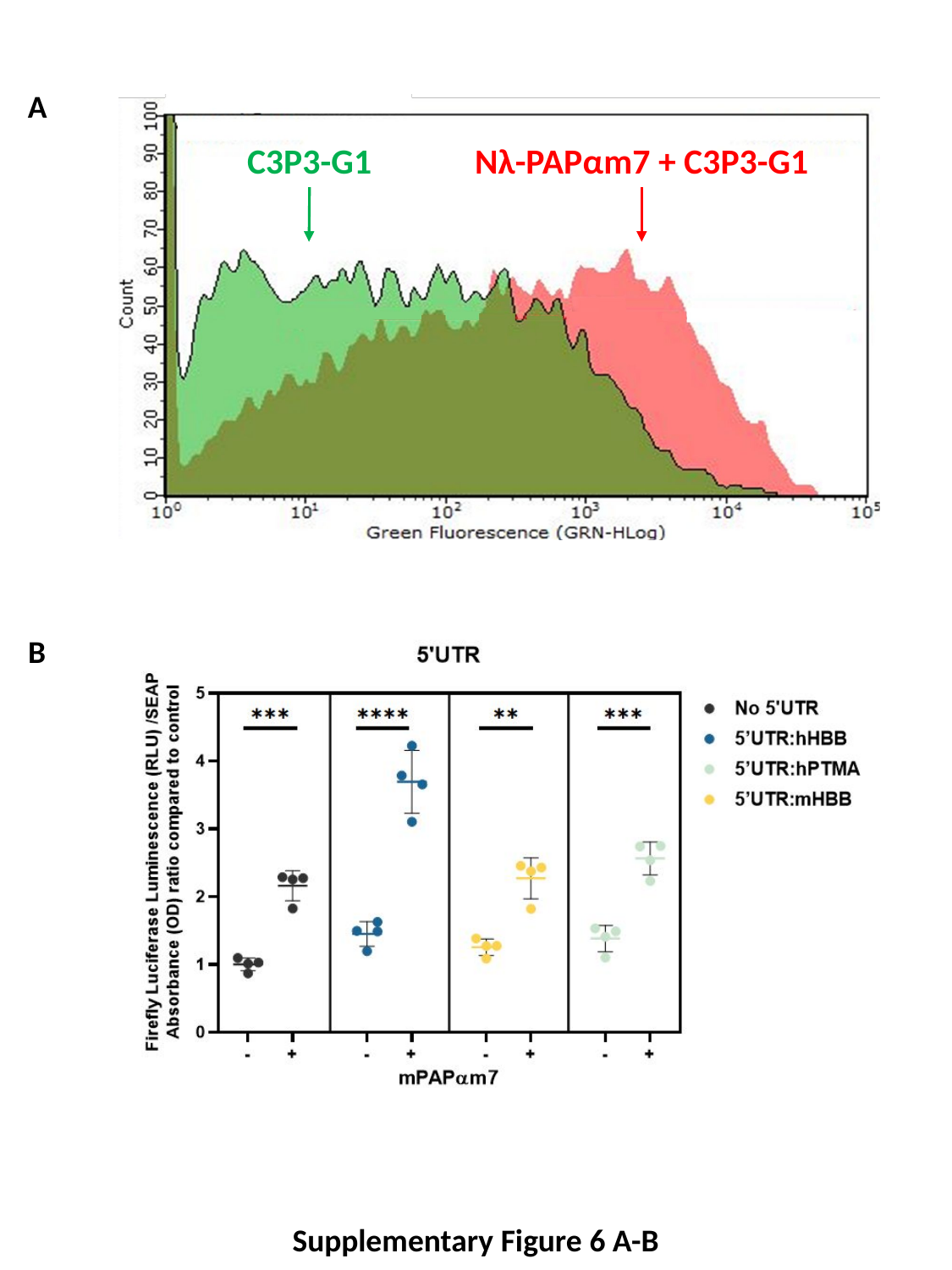

A
C3P3-G1
Nλ-PAPαm7 + C3P3-G1
B
Supplementary Figure 6 A-B

## Slide 12
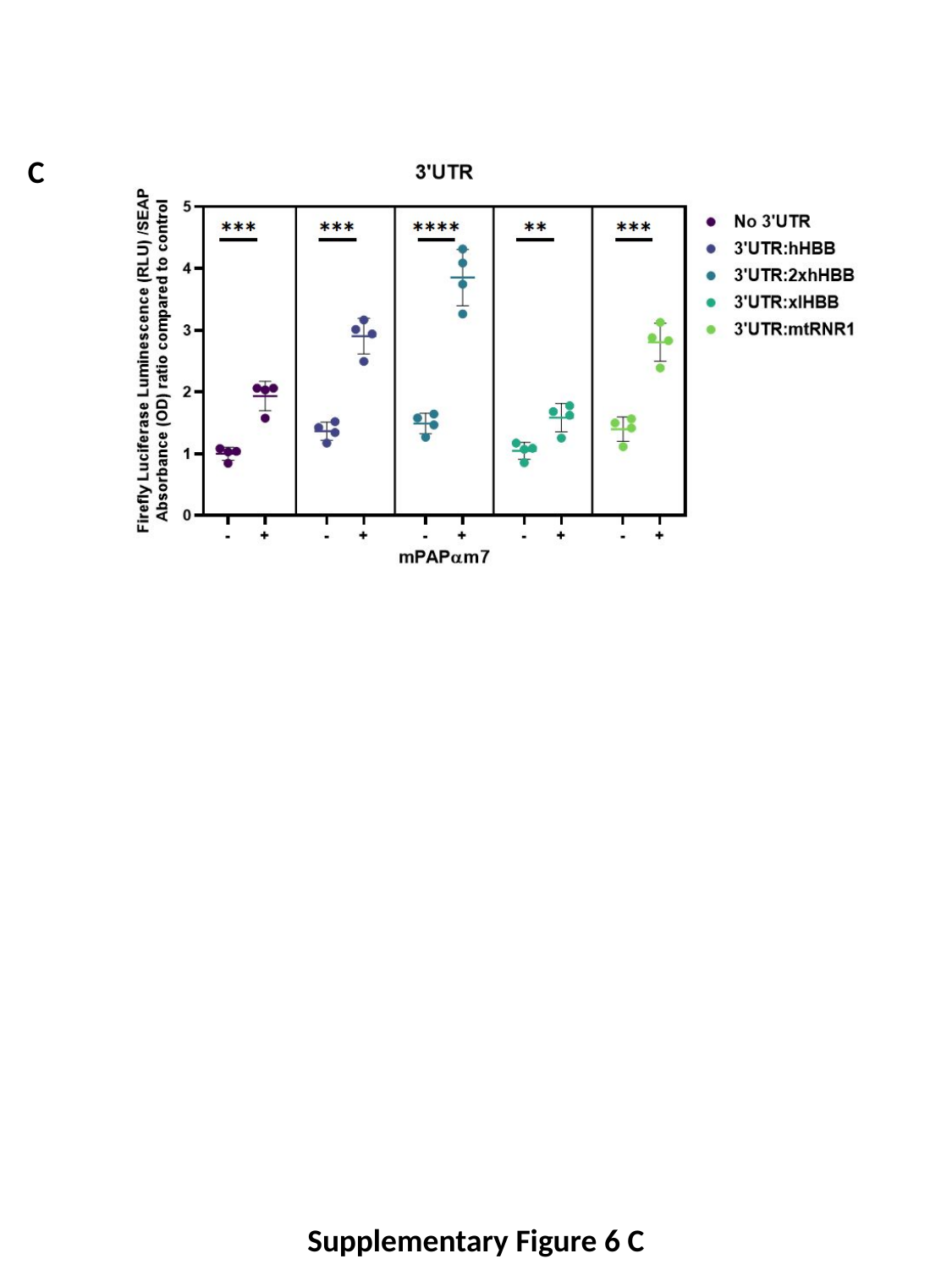

C
Supplementary Figure 6 C

## Slide 13
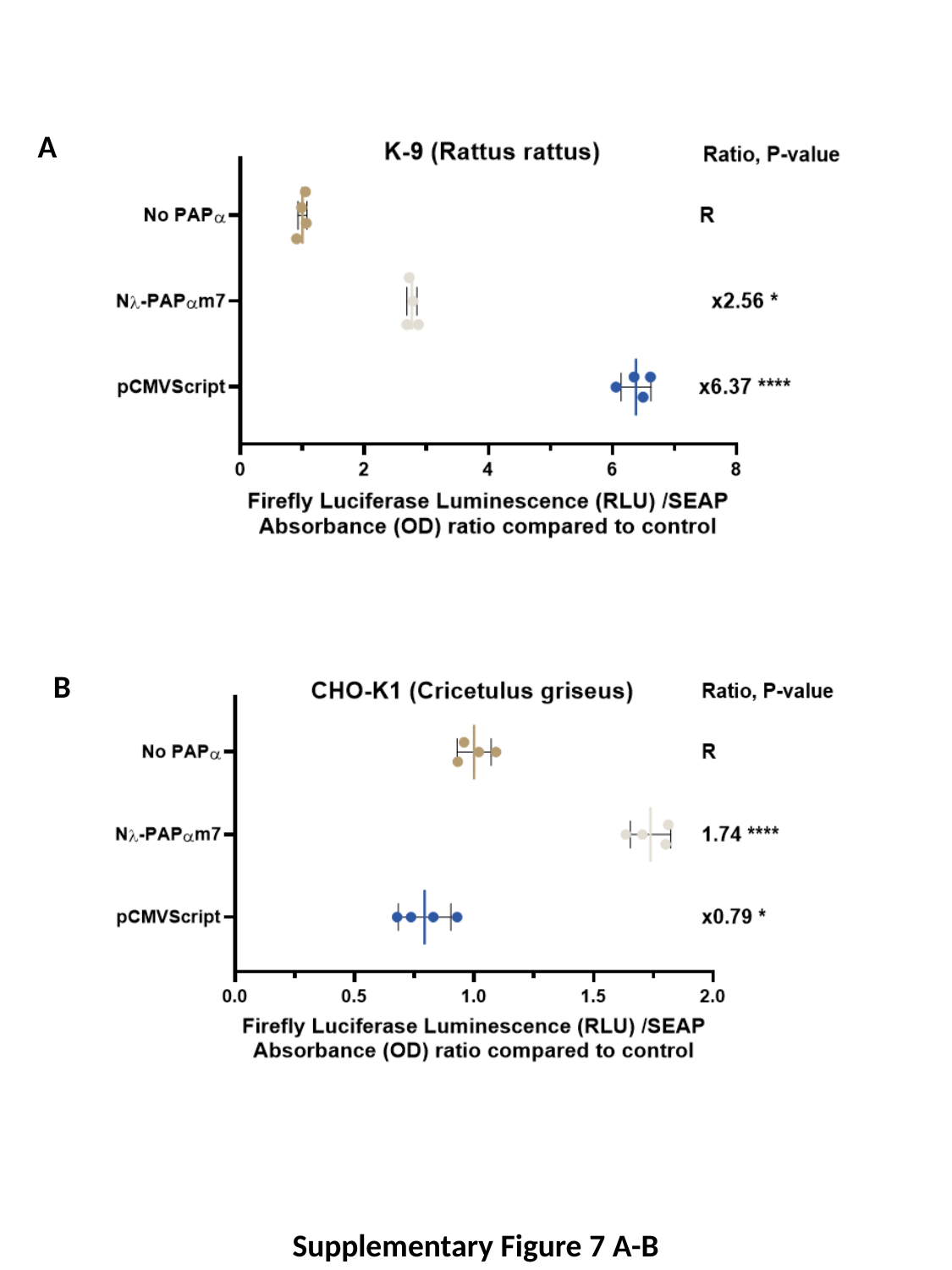

A
B
Supplementary Figure 7 A-B

## Slide 14
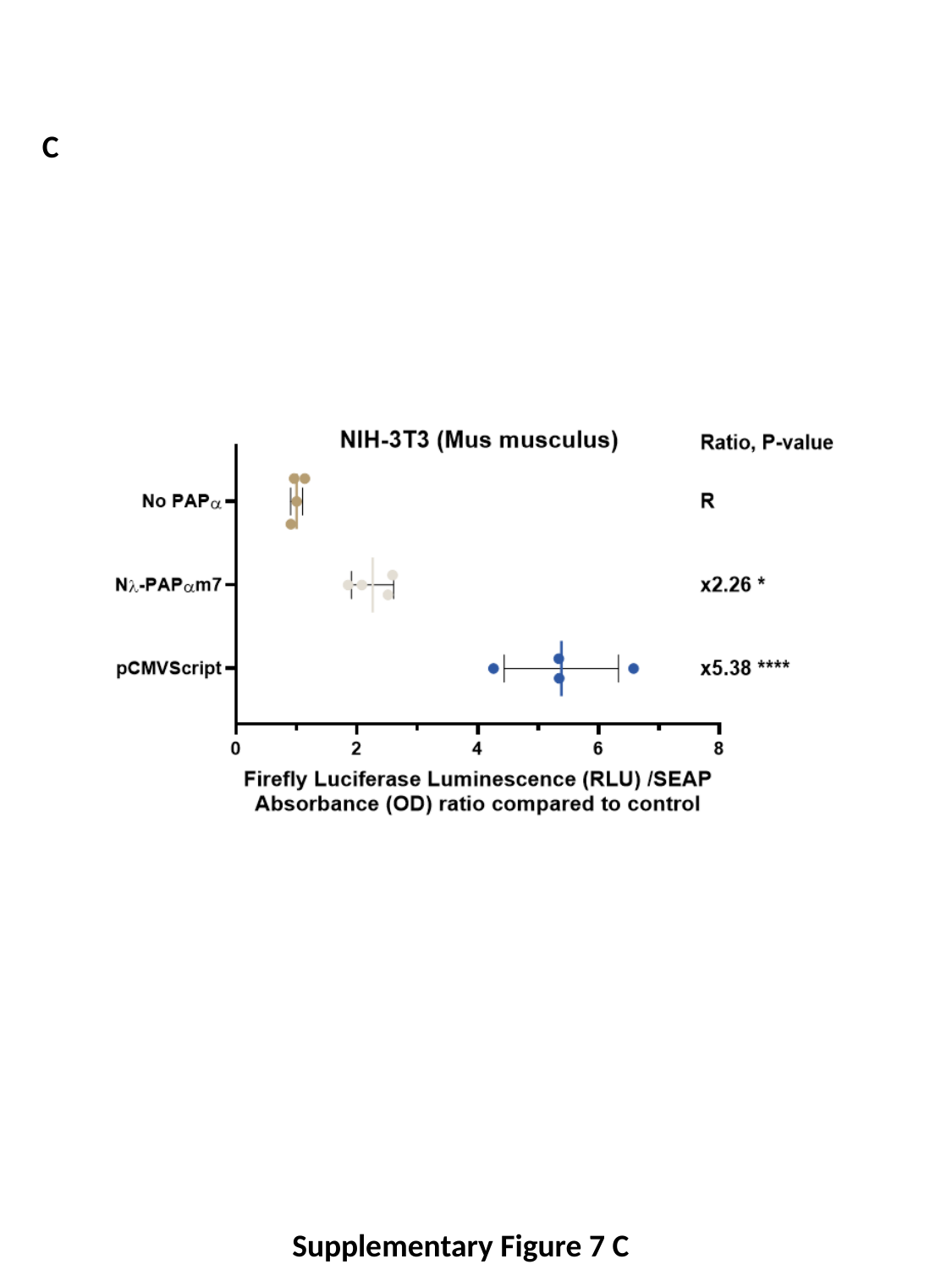

C
Supplementary Figure 7 C

## Slide 15
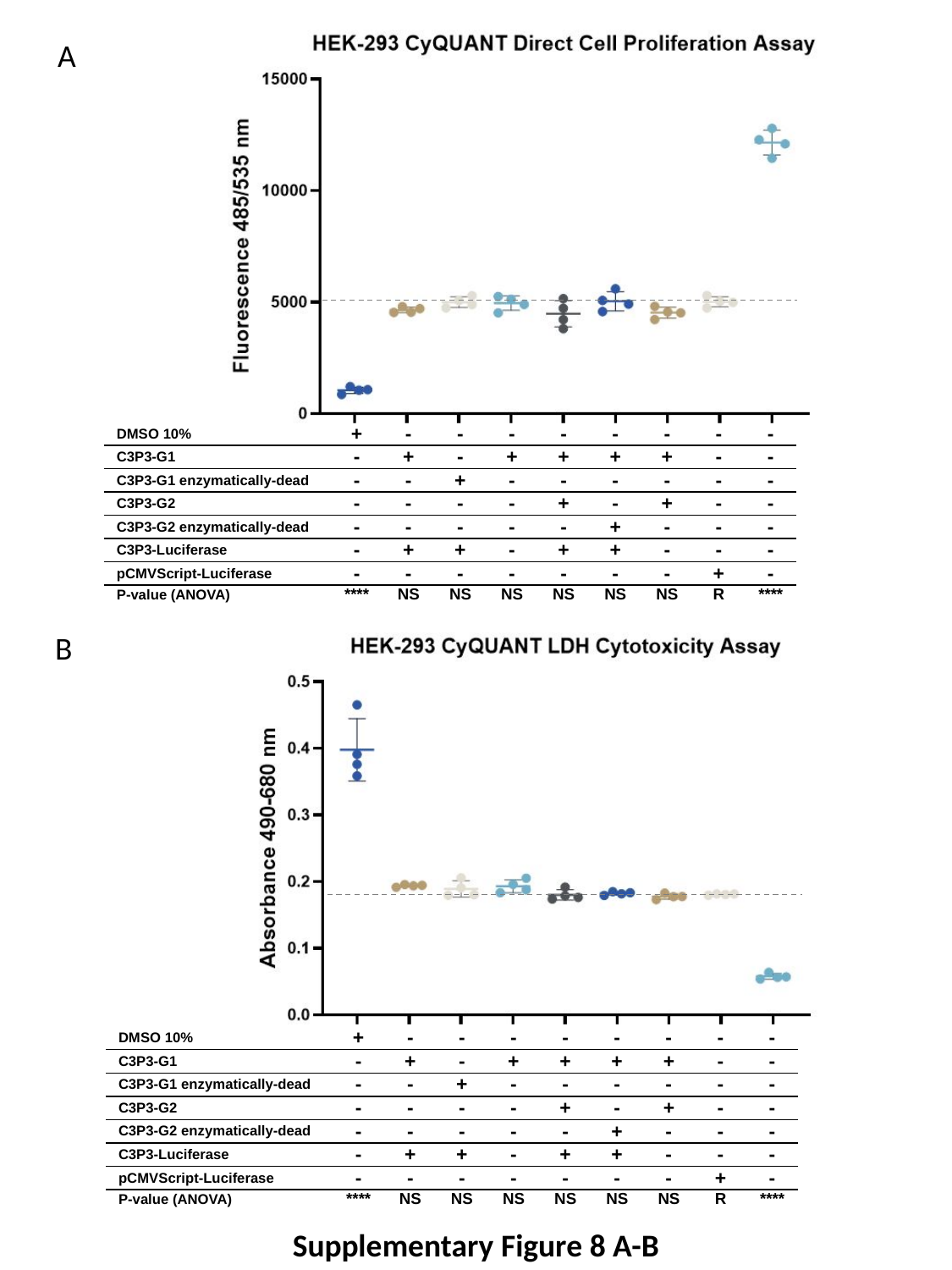

A
| DMSO 10% | + | - | - | - | - | - | - | - | - |
| --- | --- | --- | --- | --- | --- | --- | --- | --- | --- |
| C3P3-G1 | - | + | - | + | + | + | + | - | - |
| C3P3-G1 enzymatically-dead | - | - | + | - | - | - | - | - | - |
| C3P3-G2 | - | - | - | - | + | - | + | - | - |
| C3P3-G2 enzymatically-dead | - | - | - | - | - | + | - | - | - |
| C3P3-Luciferase | - | + | + | - | + | + | - | - | - |
| pCMVScript-Luciferase | - | - | - | - | - | - | - | + | - |
| P-value (ANOVA) | \*\*\*\* | NS | NS | NS | NS | NS | NS | R | \*\*\*\* |
B
| DMSO 10% | + | - | - | - | - | - | - | - | - |
| --- | --- | --- | --- | --- | --- | --- | --- | --- | --- |
| C3P3-G1 | - | + | - | + | + | + | + | - | - |
| C3P3-G1 enzymatically-dead | - | - | + | - | - | - | - | - | - |
| C3P3-G2 | - | - | - | - | + | - | + | - | - |
| C3P3-G2 enzymatically-dead | - | - | - | - | - | + | - | - | - |
| C3P3-Luciferase | - | + | + | - | + | + | - | - | - |
| pCMVScript-Luciferase | - | - | - | - | - | - | - | + | - |
| P-value (ANOVA) | \*\*\*\* | NS | NS | NS | NS | NS | NS | R | \*\*\*\* |
Supplementary Figure 8 A-B

## Slide 16
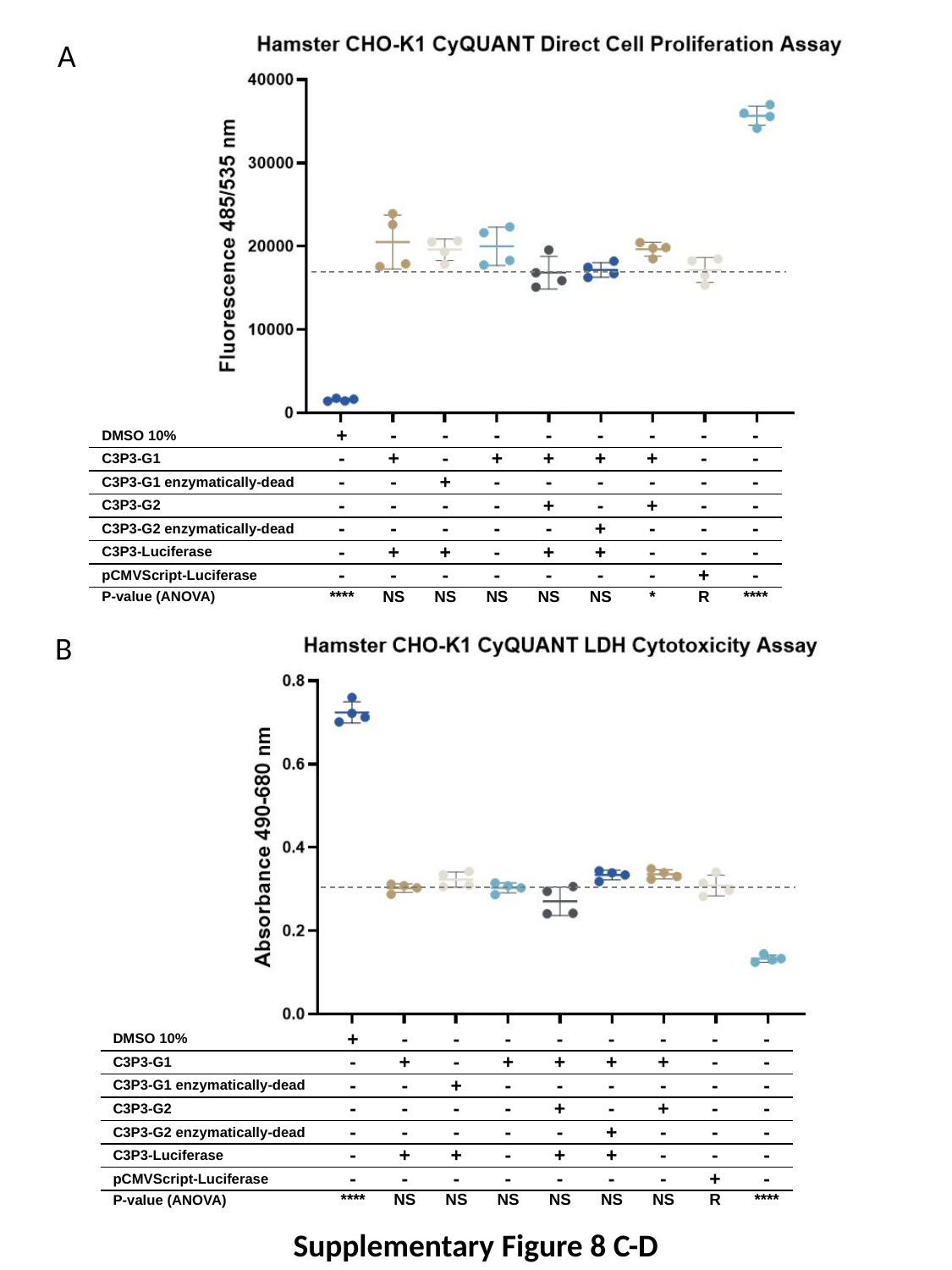

A
| DMSO 10% | + | - | - | - | - | - | - | - | - |
| --- | --- | --- | --- | --- | --- | --- | --- | --- | --- |
| C3P3-G1 | - | + | - | + | + | + | + | - | - |
| C3P3-G1 enzymatically-dead | - | - | + | - | - | - | - | - | - |
| C3P3-G2 | - | - | - | - | + | - | + | - | - |
| C3P3-G2 enzymatically-dead | - | - | - | - | - | + | - | - | - |
| C3P3-Luciferase | - | + | + | - | + | + | - | - | - |
| pCMVScript-Luciferase | - | - | - | - | - | - | - | + | - |
| P-value (ANOVA) | \*\*\*\* | NS | NS | NS | NS | NS | \* | R | \*\*\*\* |
B
| DMSO 10% | + | - | - | - | - | - | - | - | - |
| --- | --- | --- | --- | --- | --- | --- | --- | --- | --- |
| C3P3-G1 | - | + | - | + | + | + | + | - | - |
| C3P3-G1 enzymatically-dead | - | - | + | - | - | - | - | - | - |
| C3P3-G2 | - | - | - | - | + | - | + | - | - |
| C3P3-G2 enzymatically-dead | - | - | - | - | - | + | - | - | - |
| C3P3-Luciferase | - | + | + | - | + | + | - | - | - |
| pCMVScript-Luciferase | - | - | - | - | - | - | - | + | - |
| P-value (ANOVA) | \*\*\*\* | NS | NS | NS | NS | NS | NS | R | \*\*\*\* |
Supplementary Figure 8 C-D

## Slide 17
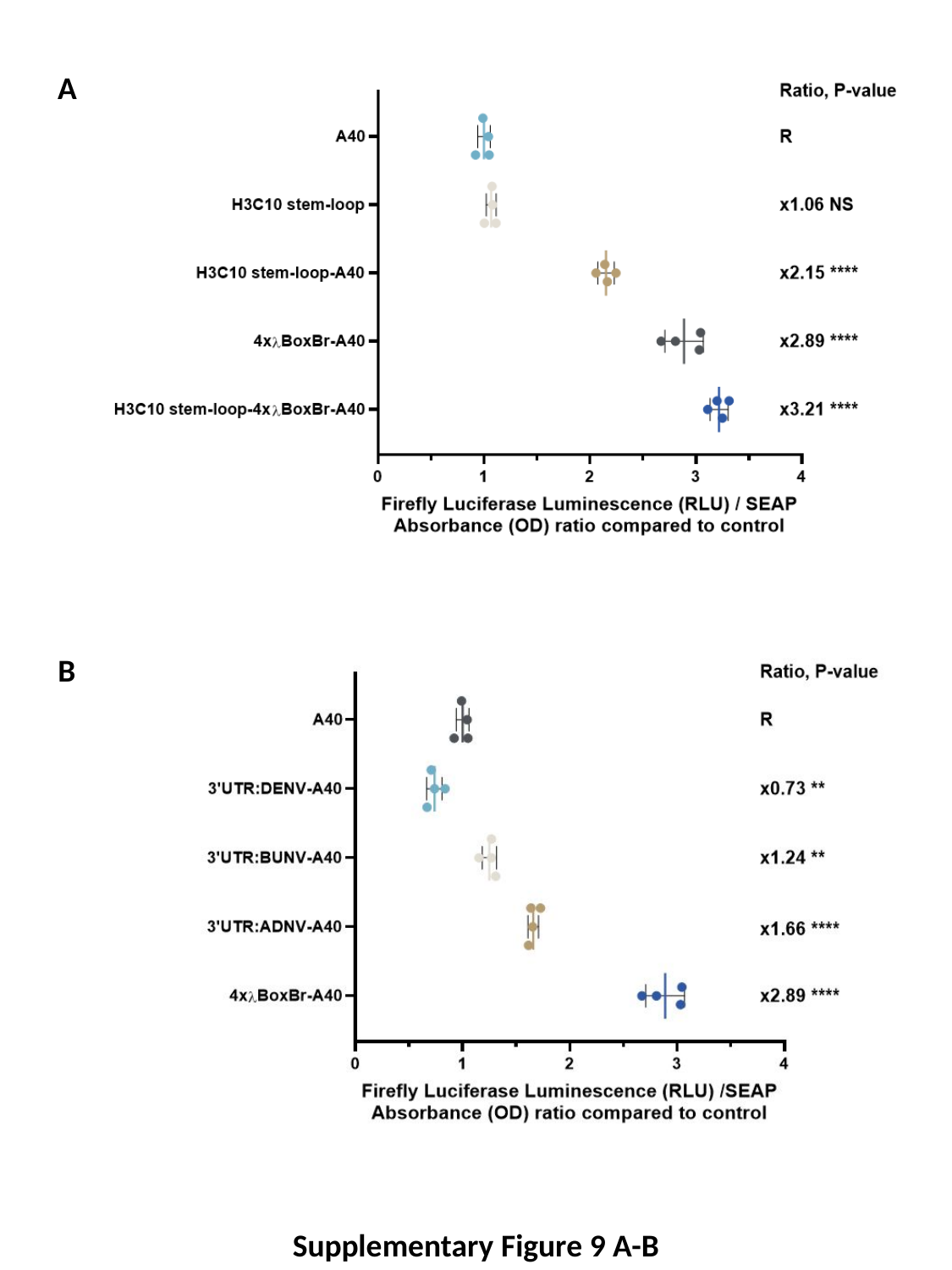

A
B
Supplementary Figure 9 A-B

## Slide 18
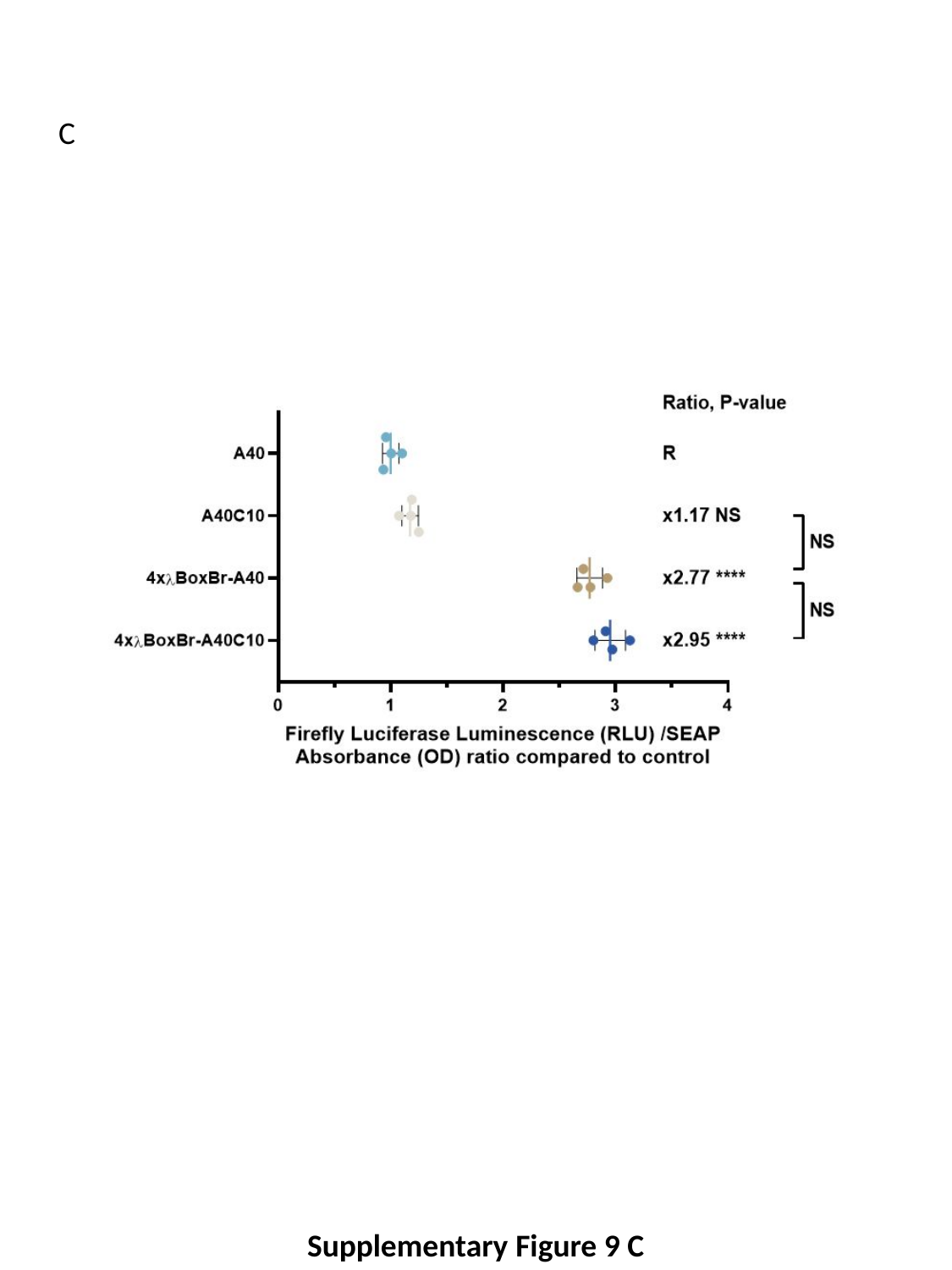

C
Supplementary Figure 9 C

## Slide 19
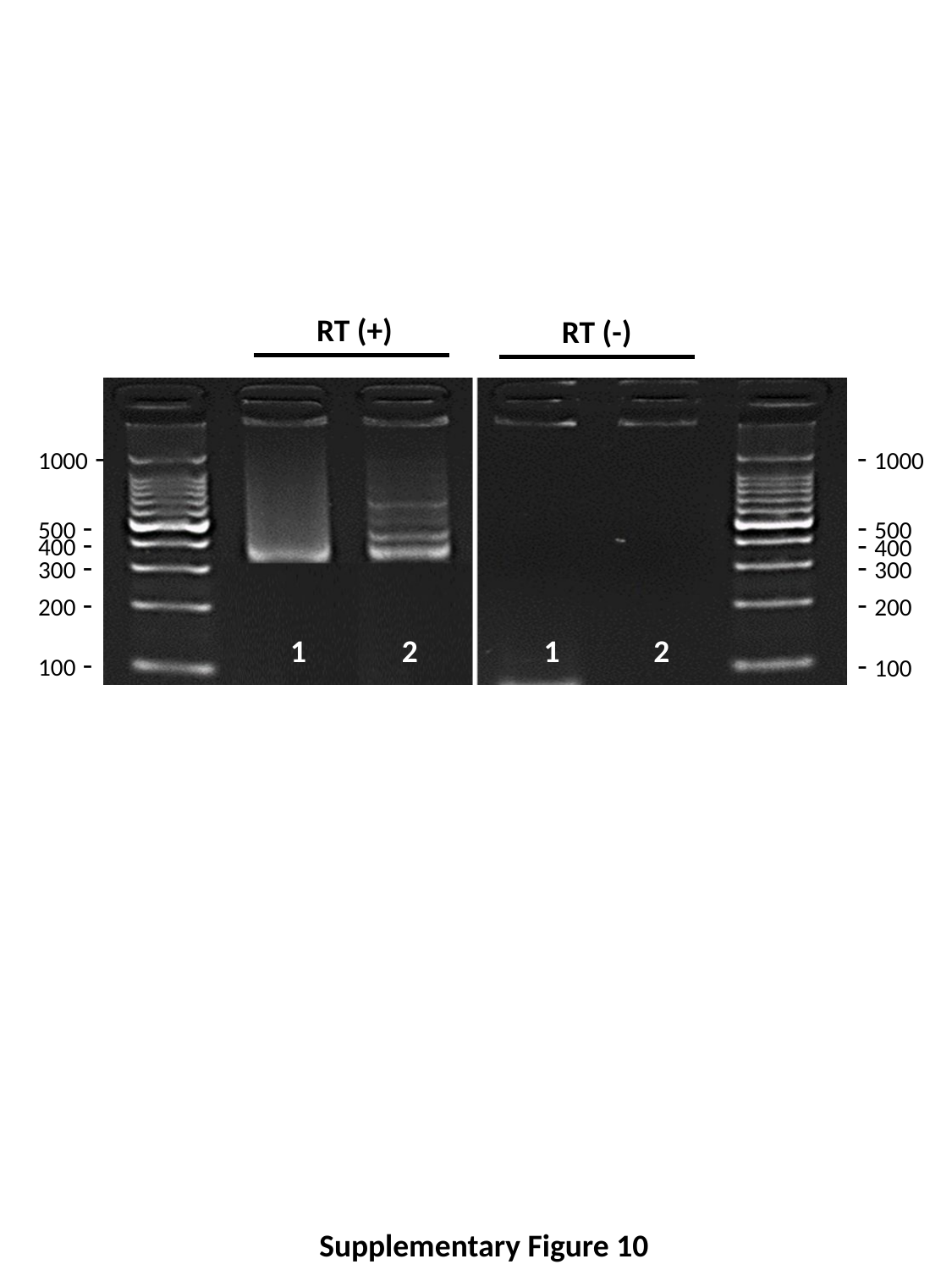

RT (+)
RT (-)
1000 -
500 -
400 -
300 -
200 -
100 -
- 1000
- 500
- 400
- 300
- 200
- 100
1
2
1
2
Supplementary Figure 10

## Slide 20
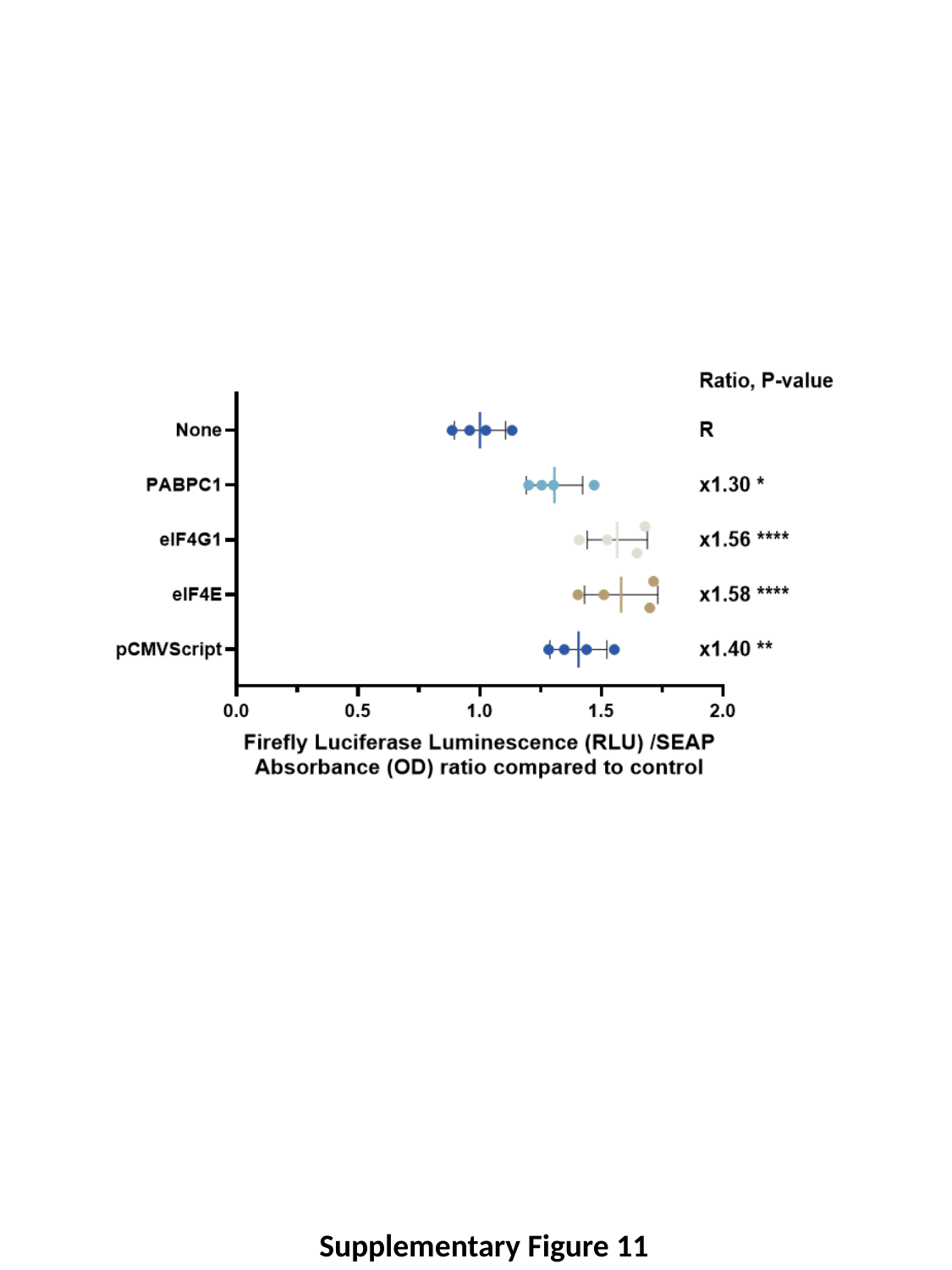

Supplementary Figure 11
