# Supplementary material for: Rational design of an artificial tethered enzyme for non-templated post-transcriptional mRNA polyadenylation by the second generation of the C3P3 system
Source: Sci Rep. 2024 Mar 2;14:5156. doi: 10.1038/s41598-024-55947-0 (PMC10908868; doi:10.1038/s41598-024-55947-0)
Supplement: Supplementary file 2 — Supplementary Information 2. [file 41598_2024_55947_MOESM2_ESM.docx]

***Supplementary Material and Data for:***

**TITLE: Rational design of an artificial tethered enzyme for non-templated post-transcriptional mRNA polyadenylation** **by the second generation of the C3P3 system**

RUNNING TITLE: Non-templated polyadenylation enzyme for the second generation of the C3P3 system

Classification: biological sciences, System Biology

Keywords: C3P3, mRNA, polyadenylation, poly(A) polymerase, artificial expression system

Authors: Marine Le Boulch, M.S.^1^, Eric Jacquet, Ph.D.^2^, Naïma Nhiri, Ph.D.^2^, Maya Shmulevitz, Ph.D.^3^, Philippe H. Jaïs, M.D. Ph.D.^1 *^

^1^ Eukarÿs SAS, Pépinière Genopole, 4 rue Pierre Fontaine, 91000 Evry-Courcouronnes, France,

^2^ Institut de Chimie des Substances Naturelles, CNRS UPR2301, Université Paris-Saclay, Avenue de la Terrasse, 91198 Gif-sur-Yvette, France,

^3^ Medical Microbiology and Immunology, Li Ka Shing Institute of Virology, University of Alberta, 6-142J Katz Group Centre for Pharmacy & Health Research, 114 Street NW, Edmonton, Alberta, T6G 2E1, Canada.

^*^ To whom correspondence should be addressed.

Address for correspondence

Philippe H. Jaïs

Eukarÿs SAS

Genopole Entreprises Campus 3

4 rue Pierre Fontaine

91000 Evry-Courcouronnes, France

## Supplementary Material and Data 1. Sequences of siRNA targeting human cyclin B and human p34^cdc2^ (pools of four siRNA).

| **Target gene** | **Sequences (pool), sense strand** |
| --- | --- |
| human cyclin B (CCNB1) | CAACAUUACCUGUCAUAUA  CCAAAUACCUGAUGGAACU  GAAAUGUACCCUCCAGAAA  ACUGUAGGGUAGCGGAAAA |
| human p34^cdc2^ (CDK1) | GUACAGAUCUCCAGAAGUA  GAUCAACUCUUCAGGAUUU  GGUUAUAUCUCAUCUUUGA  GAACUUCGUCAUCCAAAUA |

## Supplementary Material and Data 2. Primers and TaqMan MGB probes for RT-qPCR.

| **Target mRNA** | **Forward primer (5'🡪3')** | **Reward primer (5'🡪3')** | **TaqMan MGB probe** |
| --- | --- | --- | --- |
| Luciferase | GTTCCATTCCATCACGGTTTTGG | AGCGCACTTTGAATCTTGTAATCCT | AAGGCTCCTCAGAAACA |
| C3P3-G1 | CTGCCTGCGGAAGAACCT | CAGGTCCTGCTCGACACT | TCCGCCTCCGCCTGAT |
| ACTB | Hs01060665_g1 (Taqman gene expression Assay, Life Technologies) | | |
| GAPDH | Hs02758991_g1 (Taqman gene expression Assay, Life Technologies) | | |

## Supplementary Material and Data 3. Normalization method for RT-qPCR

The copy number normalization technique used for the measures of translatability relied on the following steps. First of all, the purified RNAs were quantified by nanodrop and by Bioanalyzer, which made it possible to carry out the reverse transcription under similar conditions for all the samples. The GAPDH and ACTB mRNAs were qualified by RT-PCR simultaneously with the mRNA of interest. The data obtained by RT-qPCR for luciferase were thus corrected by the possible variations of these GAPDH and ACTB reference genes in each of the samples. Finally, the exact determination of the copy number is made by using a reference plasmid carrying the sequences of the amplicons quantified in RT-qPCR. This reference plasmid, whose concentration is known, is used to construct a range which makes it possible to correlate copy number and Ct, which is used to determine the copy number in the initial sample. We are therefore confident that this technique allows for robust quantification of mRNA.

## Supplementary Material and Data 4. Primers for Poly(A) Tailing Assay.

| **Firefly Luciferase-specific Primers** | **Sequence (5'🡪3')** | **PCR product length in the absence of poly(A) tail extension** |
| --- | --- | --- |
| Forward primer#1 (Figure 6B) | TATGAAGGGCCTTGAGCATCTG | 65 bp |
| Forward primer#2 (Supplementary Figure 9) | CGACCAAAGGTTCCTTTGTGG | 321 bp |
